# A synopsis of the genus *Ethmia* Hübner in Costa Rica: biology, distribution, and description of 22 new species (Lepidoptera, Gelechioidea, Depressariidae, Ethmiinae), with emphasis on the 42 species known from Área de Conservación Guanacaste

**DOI:** 10.3897/zookeys.461.8377

**Published:** 2014-12-09

**Authors:** Eugenie Phillips-Rodríguez, Jerry A. Powell, Winnie Hallwachs, Daniel H. Janzen

**Affiliations:** 1 Instituto Nacional de Biodiversidad (INBio), 22-3100 Santo Domingo de Heredia, Costa Rica; 2 Essig Museum of Entomology, University of California, Berkeley, CA 94720, USA; 3 Department of Biology, University of Pennsylvania, Philadelphia, PA 19104, USA

**Keywords:** Neotropical, Central America, Costa Rica, Área de Conservación Guanacaste, ACG, Lepidoptera, Gelechioidea, Depressariidae, Elachistidae, Ethmiinae, new species, food plants, caterpillars, distribution, morphology, DNA barcoding

## Abstract

We discuss 45 Costa Rican species of *Ethmia* Hübner, 1819, including 23 previously described: *Ethmia delliella* (Fernald), *Ethmia bittenella* (Busck), *Ethmia festiva* Busck, *Ethmia scythropa* Walsingham, *Ethmia perpulchra* Walsingham, *Ethmia terpnota* Walsingham, *Ethmia elutella* Busck, *Ethmia janzeni* Powell, *Ethmia ungulatella* Busck, *Ethmia exornata* (Zeller), *Ethmia phylacis* Walsingham, *Ethmia mnesicosma* Meyrick, *Ethmia chemsaki* Powell, *Ethmia baliostola* Walsingham, *Ethmia duckworthi* Powell, *Ethmia sandra* Powell, *Ethmia nigritaenia* Powell, *Ethmia catapeltica* Meyrick, *Ethmia lichyi* Powell, *Ethmia transversella* Busck, *Ethmia similatella* Busck, *Ethmia hammella* Busck, *Ethmia linda* Busck, and 22 new species: *Ethmia blaineorum*, *Ethmia millerorum*, *Ethmia dianemillerae*, *Ethmia adrianforsythi*, *Ethmia stephenrumseyi*, *Ethmia berndkerni*, *Ethmia dimauraorum*, *Ethmia billalleni*, *Ethmia ehakernae*, *Ethmia helenmillerae*, *Ethmia johnpringlei*, *Ethmia laphamorum*, *Ethmia petersterlingi*, *Ethmia lesliesaulae*, *Ethmia turnerorum*, *Ethmia normgershenzi*, *Ethmia nicholsonorum*, *Ethmia hendersonorum*, *Ethmia randyjonesi*, *Ethmia randycurtisi*, *Ethmia miriamschulmanae* and *Ethmia tilneyorum*. We illustrate all species and their male and female genitalia, along with distribution maps of Costa Rican localities. Immature stages are illustrated for 11 species, and food plants are listed when known. Gesneriaceae is added as a new food plant family record for *Ethmia*. CO1 nucleotide sequences (“DNA barcodes”) were obtained for 41 of the species.

## Introduction

The genus *Ethmia* Hübner, 1819 is included in the subfamily Ethmiinae, a subfamily composed of moderate-sized moths with a worldwide distribution (Powell 1973, 1985; Powell and Opler 2009). Recent molecular and morphological analyses place Ethmiinae within Depressariidae (Heikkilä et al 2013), though this subfamily has been previously treated as Elachistidae and so labeled in literature between 1999 and 2013.

Becker (1984) recorded 96 species of Neotropical *Ethmia*, distributed from Argentina to USA (Arizona and from Florida to Texas) and the Caribbean. Powell’s (1973) synoptic treatment of *Ethmia* of the same area recorded ten Costa Rican species. The national-level collecting during the last three decades by the Costa Rican Instituto Nacional de Biodiversidad (INBio) and the Lepidoptera inventory of Área de Conservación Guanacaste (ACG) in northwestern Costa Rica (Janzen et al 2009, Janzen and Hallwachs 2013) have generated additional life history information and resulted in the discovery of 35 additional Costa Rican species of *Ethmia*. We also have added DNA barcoding data to this new set of information, demonstrating its utility in disentangling species complexes first detected by Powell (1973). However, the *Ethmia* collection records for Costa Rica other than ACG are woefully few and hence not representative of the actually in-country distributions. Prior to DNA barcoding, which began in 2004, a large number of specimens collected during routine light trapping were ignored because it was assumed they were common, widespread species.

Worldwide information on the biology of *Ethmia* documents Boraginaceae as its most frequently used food plant family (Powell 1973, 1985; Powell and Opler 2009). However, caterpillar rearing by Janzen and Hallwachs (2013) in Costa Rica has discovered Gesneriaceae as an additional previously unknown food plant family.

Of the 45 species of *Ethmia* found in Costa Rica, 22 are new. We illustrate all of the species and their male and female genitalia. CO1 nucleotide sequences (“DNA barcodes”) were obtained for 41 of the species and geographic distribution maps are included for all species. Caterpillar images are provided for 11 of the species.

## Methods

**Material examined.** We examined 3000 + specimens in the INBio collections, Essig Museum of the University of California, Berkeley, or the inventory of Lepidoptera of Área de Conservación Guanacaste (ACG) (http://janzen.bio.upenn.edu/caterpillars) (Janzen and Hallwachs 2013). All of the inventory specimens will be eventually deposited in the Essig Museum, INBio and the U.S. National Museum of Natural History (USNM), Smithsonian Institution. While this paper stresses the morphological differences among these species, all species barcoded here can be easily identified by their distinctive DNA barcodes as illustrated in the neighbor-joining (NJ) tree (Fig. 169 and Suppl. material 3).

**Morphology.** The specimens were sorted to morpho-species using wing pattern and general appearance; these units were confirmed, subdivided or combined after study of the genitalia. Dissection methodology follows that summarized in Brown and Powell (1991, 2000), except genitalia were transferred to 95% isopropyl alcohol (instead of xylene), and all parts were slide-mounted with euparal mounting medium. Forewing length is measured from center of the wing base to the apex. The range and number of specimens (n) measured is indicated where appropriate.

Images of genitalia were captured using a S2CTV Olympus Stereomicroscope with a JVC3-CCD video camera and enhanced using Auto Montage (Version 3.03.0103, 1997–2000) and Montage–Explorer (version 1.02.0348, 1998) software, both copyrighted ©SYNOPTICS LTD. Images of adults and caterpillars were captured using a digital camera and arranged in the figs for publication.

The descriptions of species are based on the series available rather than just the holotype. A short diagnosis is given for the described species and a more elaborate description can be found in Powell (1973). The following abbreviations are used: M= male; F= female; FW = forewing; HW = hind wing; mm = millimeters; × = times. In wing pattern descriptions the FW is divided into costal and posterior halves (from FW base to apex). Characters of genitalia used in the descriptions are illustrated in Figs 46, 52, 65, 91 and 109. Terminology for structures of the genitalia follows Klots (1970) and Powell (1973). Caterpillars are described (last instar larvae) using the following abbreviations: T = thoracic segments, A = abdominal segments; numbers following refer to the number of the segment (i.e. A3 = third abdominal segment).

The specimens examined are listed for each of the newly described species, and these are presented alphabetically by province and specific locality. The months of the year are cited in lower case Roman numerals. The following abbreviations are used in the material examined sections: N = north; S = south; E = east; W = west; Est. = Estación; ACG = Área de Conservación Guanacaste; P. N. = Parque Nacional; m = meters. Detailed information on specimens examined for all the species is presented in Suppl. material 1 (for DNA Barcoded specimens from Janzen and Hallwachs, and INBio) and Suppl. material 2 (Additional specimens, INBio). Maps were generated using iMAP with a Google Map (2009) as base.

**Rearing procedures.** Eighteen species of *Ethmia* were successfully reared from larvae collected on various food plants in the field. The rearing protocol for the ongoing survey of the Lepidoptera of Área de Conservación Guanacaste in northwestern Costa Rica is detailed in Janzen et al (2009). Each Janzen and Hallwachs reared voucher has a unique voucher code (e.g., 09-SRNP-12345), where the prefix is the last two digits of the year, the SRNP refers to the project “call letters” for Santa Rosa National Park (assigned in 1977) and the suffix is a unique number assigned within the year. Collateral information can be accessed at http://janzen.sas.upenn.edu (Janzen and Hallwachs 2013). Light-trapped specimens have six digits in the suffix of their xx-SRNP-xxxxxx voucher codes. Powell rearing lots have a unique “JAP” number based on the year and month (e.g., 85E40 = 1985, May, 40th lot number assigned). The alphanumeric interim names (e.g., *Apanteles* Rodriguez09) used for parasitoids and any other insects in the ACG inventory, are names applied to undescribed species, and are reference names linked to all collateral information, as explained in detail in Janzen et al (2009). Host plants were identified by the team of plant taxonomists of the National Herbarium of the National Museum of Natural History in Smithsonian, the plant taxonomists of Costa Rica’s Instituto Nacional de Biodiversidad (INBio), and Daniel H Janzen from his 50 years of studying the plants of Costa Rica with tight emphasis on those of ACG; the names are up-to-date with the INBio checklist of the plants of Costa Rica and the web site at http://www.tropicos.org.

We have images for 11 species of caterpillars (Figs 136–146) and food plant records (Table 1) are based on wild-caught caterpillars. The species obtained in ACG by rearing or obtained only from lights are shown in Table 2.

**Table 1.** *Ethmia* from Costa Rica. Food plant summary. Number (n) of rearing records for each plant species and family.

Species	Plant family	Food plant species
*Ethmia baliostola*	Boraginaceae (253)	*Bourreria oxyphylla* (34), *Bourreria costaricensis* (219)
*Ethmia berndkerni*	Boraginaceae (12)	*Bourreria costaricensis* (12)
*Ethmia catapeltica*	Boraginaceae (272)	*Cordia alliodora* (272)
*Ethmia delliella*	Boraginaceae (2)	*Cordia alliodora* (2)
*Ethmia dianemillerae*	Boraginaceae (1)	*Cordia alliodora* (1)
*Ethmia lesliesaulae*	Gesneriaceae (68)	*Drymonia macrophylla* (23), *Drymonia serrulata* (41), *Drymonia warszewicziana* (2), *Drymonia alloplectoides* (2)
*Ethmia lichyi*	Boraginaceae (206)	*Cordia bicolor* (91), *Cordia collococca* (40), *Cordia eriostigma* (7), *Cordia panamensis* (29), *Cordia porcata* (39)
*Ethmia millerorum*	Boraginaceae (39)	*Bourreria costaricensis* (39)
*Ethmia miriamschulmanae*	Boraginaceae (2)	*Varronia guanacastensis* (2)
*Ethmia mnesicosma*	Boraginaceae (290)	*Cordia alliodora* (290)
*Ethmia nicholsonorum*	Boraginaceae (11)	*Cordia panamensis* (11)
*Ethmia nigritaenia*	Boraginaceae (1)	*Cordia gerascanthus* (1)
*Ethmia normgershenzi*	Gesneriaceae (39)	*Drymonia alloplectoides* (26), *Drymonia macrophylla* (4), *Drymonia serrulata* (9)
*Ethmia petersterlingi*	Boraginaceae (5)	*Cordia alliodora* (5)
*Ethmia scythropa*	Boraginaceae (263)	*Bourreria oxyphylla* (180), *Bourreria costaricensis* (83)
*Ethmia similatella*	Boraginaceae (1)	*Varronia guanacastensis* (1)
*Ethmia tilneyorum*	Boraginaceae (4)	*Cordia gerascanthus* (4)
*Ethmia turnerorum*	Boraginaceae (67)	*Cordia panamensis* (67)

**Table 2.** *Ethmia* species found in Área de Conservación Guanacaste (ACG), type of forest. Reared (**) or light-trapped specimens (*).

Species	Forest type	Reared /Light
*Ethmia adrianforsythi*	Rain Forest	(*)
*Ethmia baliostola*	Rain Forest	(**) (*)
*Ethmia berndkerni*	Rain Forest	(**) (*)
*Ethmia billalleni*	Rain Forest	(*)
*Ethmia bittenella*	Dry Forest	(*)
*Ethmia blaineorum*	Dry Forest	(*)
*Ethmia catapeltica*	Rain Forest	(**) (*)
*Ethmia chemsaki*	Dry Forest	(*)
*Ethmia delliella*	Dry Forest	(**) (*)
*Ethmia dianemillerae*	Rain Forest	(**) (*)
*Ethmia dimauraorum*	Rain Forest	(*)
*Ethmia duckworthi*	Rain Forest	(*)
*Ethmia ehakernae*	Rain Forest	(*)
*Ethmia elutella*	Dry Forest	(*)
*Ethmia exornata*	Rain Forest/Dry Forest	(*)
*Ethmia festiva*	Dry Forest	(*)
*Ethmia hammella*	Rain Forest	(*)
*Ethmia helenmillerae*	Dry Forest	(*)
*Ethmia janzeni*	Rain Forest/Dry Forest	(*)
*Ethmia johnpringlei*	Dry Forest	(*)
*Ethmia laphamorum*	Dry Forest	(*)
*Ethmia lesliesaulae*	Rain Forest	(**) (*)
*Ethmia lichyi*	Rain Forest	(**) (*)
*Ethmia linda*	Rain Forest	(*)
*Ethmia millerorum*	Rain Forest	(**) (*)
*Ethmia miriamschulmanae*	Rain Forest/Dry Forest	(**) (*)
*Ethmia mnesicosma*	Rain Forest/Dry Forest	(**) (*)
*Ethmia nicholsonorum*	Rain Forest	(**)
*Ethmia nigritaenia*	Dry Forest	(**) (*)
*Ethmia normgershenzi*	Rain Forest	(**) (*)
*Ethmia perpulchra*	Rain Forest	(*)
*Ethmia petersterlingi*	Rain Forest	(**) (*)
*Ethmia phylacis*	Dry Forest	(*)
*Ethmia randyjonesi*	Rain Forest	(*)
*Ethmia scythropa*	Rain Forest	(**) (*)
*Ethmia similatella*	Dry Forest	(**) (*)
*Ethmia stephenrumseyi*	Rain Forest	(*)
*Ethmia terpnota*	Rain Forest	(*)
*Ethmia tylneyorum*	Dry Forest	(**) (*)
*Ethmia transversella*	Rain Forest	(*)
*Ethmia turnerorum*	Rain Forest/Dry Forest	(**) (*)
*Ethmia ungulatella*	Rain Forest/Dry Forest	(*)

**Table 3.** Ethmia from Costa Rica. CO1 Barcode Gap analysis. Distances (%). Maximum intra-specific variation (Max Intra-Sp) and Nearest Neighbor (NN) distance.

Species	Max Intra-Sp	Nearest Species	Distance to NN
*Ethmia adrianforsythi*	0	*Ethmia mnesicosma*	6.38
*Ethmia baliostola*	1.12	*Ethmia laphamorum*	7.71
*Ethmia berndkerni*	0.77	*Ethmia johnpringlei*	5.24
*Ethmia billalleni*	0.31	*Ethmia duckworthi*	3.59
*Ethmia bittenella*	0.66	*Ethmia delliella*	6.13
*Ethmia blaineorum*	0.62	*Ethmia festiva*	5.07
*Ethmia catapeltica*	0.31	*Ethmia petersterlingi*	3.97
*Ethmia chemsaki*	0.17	*Ethmia stephenrumseyi*	6.52
*Ethmia delliella*	1.65	*Ethmia bittenella*	6.13
*Ethmia dianemillerae*	0.15	*Ethmia exornata*	6.67
*Ethmia dimauraorum*	0.62	*Ethmia berndkerni*	8.95
*Ethmia duckworthi*	0.18	*Ethmia billalleni*	3.59
*Ethmia ehakernae*	0.15	*Ethmia billalleni*	5.85
*Ethmia elutella*	0.31	*Ethmia janzeni*	2.82
*Ethmia exornata*	0.62	*Ethmia dianemillerae*	6.67
*Ethmia festiva*	0.92	*Ethmia blaineorum*	5.07
*Ethmia hammella*	0.62	*Ethmia helenmillerae*	2.43
*Ethmia helenmillerae*	0.48	*Ethmia hammella*	2.43
*Ethmia hendersonorum*	0.15	*Ethmia lichyi*	4.36
*Ethmia janzeni*	0.93	*Ethmia elutella*	2.82
*Ethmia johnpringlei*	0.15	*Ethmia berndkerni*	5.24
*Ethmia laphamorum*	0.77	*Ethmia nigritaenia*	5.81
*Ethmia lesliesaulae*	0	*Ethmia turnerorum*	4.67
*Ethmia lichyi*	0.49	*Ethmia hendersonorum*	4.36
*Ethmia linda*	0.15	*Ethmia hammella*	5.72
*Ethmia millerorum*	0.77	*Ethmia terpnota*	1.75
*Ethmia miriamschulmanae*	0.46	*Ethmia similatella*	5.22
*Ethmia mnesicosma*	1.24	*Ethmia adrianforsythi*	6.38
*Ethmia nicholsonorum*	0	*Ethmia lesliesaulae*	8.51
*Ethmia nigritaenia*	0.46	*Ethmia laphamorum*	5.81
*Ethmia normgershenzi*	0	*Ethmia tumerorum*	5.37
*Ethmia petersterlingi*	0.15	*Ethmia catapeltica*	3.97
*Ethmia phylacis*	0.31	*Ethmia mnesicosma*	8.36
*Ethmia randyjonesi*	0.62	*Ethmia similatella*	6.56
*Ethmia scythropa*	0.94	*Ethmia ungulatella*	7.09
*Ethmia similatella*	0	*Ethmia miriamschulmanae*	5.22
*Ethmia stephenrumseyi*	0	*Ethmia chemsaki*	6.52
*Ethmia terpnota*	0.52	*Ethmia millerorum*	1.75
*Ethmia transversella*	1.4	*Ethmia randyjonesi*	6.74
*Ethmia turnerorum*	0.16	*Ethmia lesliesaulae*	4.67
*Ethmia ungulatella*	0.62	*Ethmia janzeni*	6.71

**DNA barcoding.** Tissue samples (a single leg per specimen) were sent to the Canadian Center for DNA barcoding at the Ontario Biodiversity Institute (http://ibol.org) at the University of Guelph for DNA barcoding and the results are compared with neighbor-joining trees using Kimura 2-parameter distances (methods in Ratnasingham and Hebert 2007). The neighbor-joining tree produced by +1100 *Ethmia* CO1 sequences (Suppl. material 3) is represented by a simplified diagram depicting species complexes (Fig. 169). The genetic distances between barcoding sequences of CO1 for 41 species (Table 3) were extracted from BOLD systems website: Management & Analysis-Barcode Gap (http://www.boldsystems.org). See Suppl. material 1 for GenBank (www.ncbi.nlm.nih.gov/Genbank) or BOLD (www.barcodinglife.org) accession numbers.

**Depositories**

BMNH The Natural History Museum, London, England

EME Essig Museum of Entomology, University of California, Berkeley, USA

MNHU Museum für Naturkunde, Berlin, Germany

INBio Instituto Nacional de Biodiversidad, Heredia, Costa Rica

USNM National Museum of Natural History, Smithsonian Institution, Washington, DC, USA

Aiming for simplicity and a better comprehension of this species-rich and morphologically complex group of moths, we divided this study of Costa Rican *Ethmia* into two Sections, following Powell’s treatment of the genus (1973). Section I is characterized by species having a well-developed uncus and gnathos in the male genitalia, and Section II is characterized by species with the uncus and gnathos reduced or absent. This division, though not done for this purpose, also mirrors the general color patterns of the adult moths. Within each Section, the species are treated under species-groups. Each species-group is an assemblage of congeneric species with morphological similarities. The order in which species are treated follows Powell’s 1973 arrangement for the described species; the new species follow their most similar described species, as based in morphological evidence.

### List of *Ethmia* species found in Costa Rica

***Ethmia* SECTION I**

**Kirbyi species group**

*Ethmia delliella* (Fernald, 1891)

*Ethmia bittenella* (Busck, 1906)

**Cypraella species group**

*Ethmia festiva* Busck, 1914

*Ethmia blaineorum* Phillips, sp. n.

*Ethmia scythropa* Walsingham, 1912

*Ethmia perpulchra* Walsingham, 1912

*Ethmia terpnota* Walsingham, 1912

*Ethmia millerorum* Phillips, sp. n.

*Ethmia elutella* Busck, 1914

*Ethmia janzeni* Powell, 1973

*Ethmia ungulatella* Busck, 1914

**Exornata species group**

*Ethmia exornata* (Zeller, 1877)

*Ethmia dianemillerae* Phillips, sp. n.

*Ethmia adrianforsythi* Phillips, sp. n.

*Ethmia phylacis* Walsingham, 1912

*Ethmia mnesicosma*, Meyrick, 1924

**Notatella species group**

*Ethmia chemsaki* Powell, 1973

*Ethmia stephenrumseyi* Phillips, sp. n.

***Ethmia* SECTION II**

**Baliostola species group**

*Ethmia baliostola* Walsingham, 1912

**Confusella species group**

*Ethmia berndkerni* Phillips, sp. n.

*Ethmia dimauraorum* Phillips, sp. n.

*Ethmia duckworthi* Powell, 1973

*Ethmia billalleni* Phillips, sp. n.

*Ethmia ehakernae* Phillips, sp. n.

*Ethmia sandra* Powell, 1973

*Ethmia helenmillerae* Phillips, sp. n.

*Ethmia johnpringlei* Phillips, sp. n.

**Longimaculella species group**

*Ethmia nigritaenia* Powell, 1973

*Ethmia laphamorum* Phillips, sp. n.

*Ethmia catapeltica* Meyrick, 1924

*Ethmia petersterlingi* Phillips, sp. n.

*Ethmia lesliesaulae* Phillips, sp. n.

*Ethmia turnerorum* Phillips, sp. n.

*Ethmia normgershenzi* Phillips, sp. n.

*Ethmia nicholsonorum* Phillips, sp. n.

*Ethmia lichyi* Powell, 1973

*Ethmia hendersonorum* Phillips, sp. n.

*Ethmia transversella* Busck, 1914

*Ethmia randyjonesi* Phillips, sp. n.

*Ethmia randycurtisi* Phillips, sp. n.

**Trifurcella species group**

*Ethmia miriamschulmanae* Phillips, sp. n. *Ethmia similatella* Busck, 1920

*Ethmia tilneyorum* Phillips, sp. n.

**Hammella species group**

*Ethmia hammella* Busck, 1910

**Joviella species group**

*Ethmia linda* Busck, 1914

## Results

### Ethmia: Section I

In this section we treat species of *Ethmia* that are characterized by a well-developed uncus and gnathos in the male genitalia (Figs 46–63) and short posterior apophyses in the female genitalia (Figs 91–108). The general FW pattern of Section I species is simple and includes colorful patches and transverse lines (Figs 1–18). There are 18 Costa Rican species (four previously undescribed) in this Section.

#### Kirbyi species-group

**Figures 1–11.**
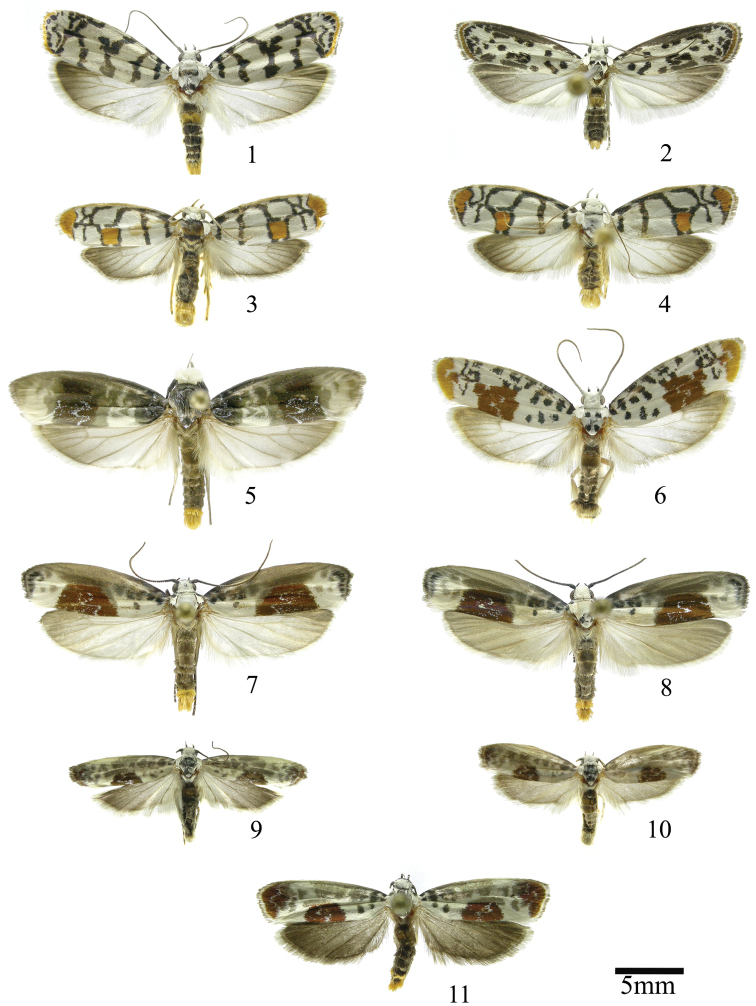
*Ethmia* from Costa Rica, adults: **1** *Ethmia delliella*, male, INB0003314923 **2** *Ethmia bittenella*, male, INBIOCRI001341767 **3** *Ethmia festiva*, male, 09-SRNP-107992 **4** *Ethmia blaineorum*, paratype, male, INBIOCRI000227642 **5** *Ethmia scythropa*, male, 06-SRNP-102759 **6** *Ethmia perpulchra*, male, INB0003146822 **7** *Ethmia terpnota*, male, 06-SRNP-102760 **8** *Ethmia millerorum*, holotype, male, 09-SRNP-36206 **9** *Ethmia elutella*, male, 11-SRNP-100961 **10** *Ethmia janzeni*, male, INB0003435319 **11** *Ethmia ungulatella*, male, 07-SRNP-106727.

This group is defined by the presence of the Sc-R crossvein in the HW and the dorsal scaling of the second abdominal segment ochreous (Figs 1–2). In Costa Rica two species are included.

##### Ethmia delliella

Taxon classificationAnimaliaLepidopteraDepressariidae

(Fernald)

Figures 1 , 46 , 91 , 136 , 147

Psecadia delliella Fernald, 1891: 29
Ethmia delliella Meyrick, 1914: 27; Powell 1973: 114.

###### Diagnosis.

This species is distinct from any other in Costa Rica by having the white FW crossed by black lines and a bright gold terminal margin.

###### Description.

Male: FW length 9.5–9.8 mm (n = 3). **Head:** Labial palpus short without reaching antennal bases. **Thorax:** Pronotal scaling white, two transverse black lines, one between the tegulae and one across scutellum. FW white, crossed by black lines and a bright golden terminal margin. HW ground color white, becoming brownish toward apex; costal area with an exposed, thin hair pencil. **Abdomen:** Dorsal scaling of second segment bright ochreous, scaling of remainder gray-brown with pale posterior and lateral; genital scaling ochreous. Genitalia (Fig. 46) with uncus broad, hood-like; basal processes elongate, 1.5× uncus length; spines of anterior portion of gnathos thin, distal part of valva with apical notch at 0.25× length of valva.

Female: FW length 10.9–11.3 mm (n = 3). **Head and thorax:** As described for male. HW unmodified. **Abdomen:** Genitalia (Fig. 91) with anterior apophyses slender; sterigma a bilobed fig; ductus bursae slightly sclerotized basally; signum a thin fold covering half of diameter of bursae with a row of short teeth.

###### Holotype.

Female: Texas, USA. [USNM, examined].

###### Distribution and biology.

*Ethmia delliella* has been collected from southern Texas and Mexico to El Salvador and Costa Rica (Powell 1973). In Costa Rica (Fig. 147) it is a common species in the dry forest of ACG and has also been collected at 1005 m in the Cordillera Volcánica de Guanacaste at the interface between ACG dry forest and cloud forest.

###### Food plants records.

*Ethmia delliella* was reared from Boraginaceae: *Cordia alliodora* (Ruiz & Pav.) Oken in Parque Nacional Santa Rosa, Guanacaste (Sector Santa Rosa, ACG). Powell collected caterpillars of this species there in June 1988; adults emerged in June and July one year later, from pupae held at Berkeley, California.

###### Immature stages

(Fig. 136). Dorsum: Head capsule and prothoracic shield orange with black dots, thorax and abdomen dorsum yellowish with black dots. Lateral: Blackish with white and yellow irregular marks.

###### Remarks.

Powell (1973) mentioned that specimens from the southern part of the range tend to be larger and possess darker HW color. The general morphology of adult and genitalia do not show major differences between specimens from Costa Rica and Texas.

##### Ethmia bittenella

Taxon classificationAnimaliaLepidopteraDepressariidae

(Busck)

Figures 2 , 47 , 92 , 147

Tamarrha bittenella Busck, 1906: 730.
Ethmia bittenella Meyrick, 1914: 28; Powell 1973: 119.

###### Diagnosis.

*Ethmia bittenella* is most similar to *Ethmia delliella* but can be distinguished by its smaller size, the FW pattern composed of groups of small dark dots, and the presence of a FW gray costal margin.

###### Description.

Male: FW length 7.8–8.3 mm (n = 4). **Head:** Scaling of front and crown smooth, white. **Thorax:** Pronotal scaling white; paired large spots, black, reflecting metallic blue, between tegulae and on scutellum. FW ground color white, markings blackish brown; a round spot on posterior half near base; gray costal margin. HW whitish basally, becoming pale brownish apically; costa dorsally with thick ochreous hair pencil from base to end of cell. **Abdomen:** Genitalia (Fig. 47) with uncus short, hood like; basal processes short, about 1.2× uncus length; spines of anterior portion of gnathos large, valva large in relation to tegumen, with apical notch 0.20× of valva.

Female: FW length 8.7–9.8 mm (n = 3). **Head and thorax:** As described for male, HW unmodified. **Abdomen:** Genitalia (Fig. 92) with sterigma wide surrounding ostium; ductus unsclerotized; signum as in *Ethmia delliella*.

###### Holotype.

Female: USA, Texas, Brownsville, in copula, 29 May 1904. USNM Type No. 9272 [USNM, examined].

###### Distribution and biology.

*Ethmia bittenella* has been collected from Texas to southern Mexico and in northwestern Costa Rica (Powell 1973). In Costa Rica (Fig. 147) it is a common species in the dry forest of ACG, and has been found from 100 to 600 m on both slopes of the Cordillera Volcánica de Guanacaste, the Cordillera Tilarán, and Península de Nicoya. The food plant and immature stages are unknown. However, barely legible labels on Texas specimens say “anacua” and “anagua”, which suggest that this species feeds on *Ehretia anacua* (Terán & Berland.) I.M. Johnst. (Boraginaceae) in Texas.

#### Cypraella species-group

This group is defined by the presence of a posterior blotch on the FW (Fig. 3–11). The valva of the male genitalia has a variable distal notch and the sterigma in the female genitalia is ornamented with lateral lobes. In Costa Rica this group includes nine species, two of them described as new here.

##### Ethmia festiva

Taxon classificationAnimaliaLepidopteraDepressariidae

Busck

Figures 3 , 48 , 93 , 148

Ethmia festiva Busck, 1914: 33; Powell 1973: 123.

###### Diagnosis.

*Ethmia festiva* can be discriminated from *Ethmia blaineorum*, its most similar species, by its smaller size and by its sacculus enlarged at 0.25× from the base of the valva.

###### Description.

Male: FW length 8.1-8.4 mm (n = 3). **Head:** Labial palpus long reaching crown, white, brownish exteriorly at II segment. **Thorax:** Whitish with black area at scutellum. FW ground color white, two brown lines from costa to posterior margin at base and antemedial area, one brown line at medial area reaching internal margin of a big ochreous square patch; apical and terminal area with a triangular ochreous patch; terminal area with a circle composed of five brownish radiating arms. HW ground color brownish becoming darker from 0.5× from base, fringe ochreous; costal area simple. **Abdomen:** Brownish dorsum, ventrally and genitalia scaling ochreous. Genitalia (Fig. 48) with uncus deeply notched, basal processes broad, short, not reaching gnathos, distal notch of valva broad, round, simple; gnathos small covered with spines. Sacculus enlarged at 0.25× from base of valva.

Female: FW length 8.9–9.8 mm (n = 3). **Head and thorax:** As described for male. **Abdomen:** Genitalia (Fig. 93) with sterigma bearing lateral lobes, not sclerotized in anterior margin; apophyses short, ductus base with a short diagonal row of small spines; signum a dentate fold.

###### Holotype.

Male: Panamá, Porto Bello, May 1912, A. Busck. USNM Type No. 16695 [USNM, examined].

###### Distribution and biology.

*Ethmia festiva* has been collected from southeastern Mexico to northwestern Colombia. In Costa Rica (Fig. 148), it has been found on both slopes of Cordillera Volcánica de Guanacaste from sea level to 1050 m. While sympatric with its sister species *Ethmia blaineorum* in ACG dry forests, *Ethmia festiva* extends its range onto the rain forested ACG Caribbean slope of northern Costa Rica. Food plant and immature stages are unknown.

##### Ethmia blaineorum

Taxon classificationAnimaliaLepidopteraDepressariidae

Phillips sp. n.

http://zoobank.org/B9E86917-344F-4CF7-8327-A137A0257289

Figures 4 , 49 , 94 , 148

###### Diagnosis.

*Ethmia blaineorum* is most similar to *Ethmia festiva* in forewing pattern and color, but *Ethmia blaineorum* is a slightly larger species. The male genitalia of *Ethmia blaineorum* is easily distinguished by the larger and more ornamented gnathos and longer basal processes. The female genitalia is distinguished by a more sclerotized anterior margin of the sterigma and by the shape of the signum: longer, curved and bearing short spines in *Ethmia blaineorum*.

###### Description.

Male: FW length 8.3-8.6 mm (n = 3). **Head:** Labial palpus whitish curved, elongate, reaching antenna base; front white brownish at base; crown white. **Thorax:** Dorsal scaling white, dark narrow band at base of tegula and anterior pronotum. FW ground color white; three distinct lines, brownish: first near base from costa to posterior margin, second at basal one fourth from below costa to posterior margin, and third at middle of wing, angling outward on costal half, reaching above a large, square, bright ochreous patch from above Cu fold to just above dorsum; apical and terminal area with a broad, triangular, bright ochreous patch; terminal area between latter and cell with an open circle composed by five brownish radiating arms. HW ground color whitish becoming brownish 0.5× from base, fringe ochreous, costal area simple. **Abdomen:** Dorsal scaling pale brownish; underside and genital scaling ochreous. Genitalia (Fig. 49) with uncus deeply notched, basal processes broad, short, surpassing base of gnathos; gnathos round, its diameter 0.3× the length of uncus from base; distal notch of valva broad, round, simple; sacculus simple, not enlarged.

Female: FW length 8.8-10.1 mm (n = 3). **Head and Thorax:** As described for male. **Abdomen:** Genitalia (Fig. 94) with sterigma bearing lateral lobes sclerotized in anterior margin; anterior apophyses short; ductus base simple; signum a long, curved dentate fold.

###### Holotype.

Male: 06-SRNP-104306, DNA Barcoded, **Costa Rica:** Guanacaste: ACG: Sector Santa Elena, La Angostura 300 m, 25.v.2006, F. Quesada & R. Franco. Deposited in INBio. **Paratypes: Costa Rica:** Guanacaste: ACG: Sector Santa Elena, La Angostura 300 m, 1M 3F 25.v.2006, 3F 25–26.v.2009, F. Quesada & R. Franco; Mirador Río Cuajiniquil 242 m, 1F, 14.vi.2007, F. Quesada & R. Franco; Manta Potrero Grande 20 m, 1F 22.v.2009, F. Quesada & H. Cambronero, 2F 23.v.2009, S. Ríos & R. Franco. Sector Santa Rosa, Luces 575 m, 1F 1.vi.2011, F. Quesada & R. Franco. (BMNH, INBio, EME, USNM).

###### Distribution and biology.

*Ethmia blaineorum* has been collected in Costa Rica (Fig. 148) in the Pacific side of Cordillera Volcánica de Guanacaste (ACG) from sea level to 600 m, just in the dry forest. Food plant and immature stages are unknown.

###### Etymology.

*Ethmia blaineorum* is named in honor of Joan and Anne Blaine of Kennett Square, Pennsylvania, for being early major donors for ACG rain forest purchase and for more than a decade of co-stimulating the entire Stroud family diaspora to be ACG supporters.

##### Ethmia scythropa

Taxon classificationAnimaliaLepidopteraDepressariidae

Walsingham

Figures 5 , 50 , 95 , 137 , 149

Ethmia scythropa Walsingham, 1912: 148; Powell 1973: 127.

###### Diagnosis.

*Ethmia scythropa* is easily distinguished from all other members of the genus in Central America by its erect, white scaling of the head and the presence of gray tufts of hair under the tegula in the male.

###### Description.

Male: FW length: 10.1–13.4 mm (n = 4). **Head:** Labial palpus elongate, proboscis and front smooth, pale grayish; crown and occipital margin with elongate, anteriorly directed, white tufts; antennal scape elongated. **Thorax:** Ground color gray, tegula with metallic black-blue and dense gray tufts under. FW ground color olivaceous gray, reflecting metallic; a large dorsal blotch dark gray reflecting metallic purplish at middle line extending to top of cell. HW ground color whitish becoming pale brownish apically, costal hair pencil dark ochreous. **Abdomen:** Brown, genital scaling bright ochreous. Genitalia (Fig. 50) with uncus barely notched; gnathos with broad, dentate posterior portion, basal processes short and thin, valva broadly emarginate distally.

Female: FW length 11.1–13.4 mm (n = 3). **Head and thorax:** As described for male except tegula without enlarged hair tufts and HW costal area simple. **Abdomen:** Genitalia (Fig. 95) with sterigma bearing rounded lateral lobes; ductus with a narrow sclerotized sleeve; signum a fold with irregular sized teeth.

###### Holotype.

Male: Costa Rica, “Banana River” [Río Banano near Limón, Limón Prov.] March 1907, W. Schaus. USNM Type No. 68206 [USNM, examined].

###### Distribution and biology.

*Ethmia scythropa* has been recorded from eastern Mexico (Jalisco and Veracruz) to Costa Rica and in Cuba and Jamaica (Powell 1973). In Costa Rica (Fig. 149) this species has been collected from 0 to 1150 m on the east side of Cordillera Volcánica de Guanacaste and Tilarán and in northern Caribbean lowlands. *Ethmia scythropa* occurs in ACG rain forest.

###### Food plant records.

*Ethmia scythropa* was reared from Boraginaceae: *Bourreria oxyphylla* Standl., *Bourreria costaricensis* (Standl.) A.H. Gentry.

###### Immature stages

(Fig. 137). Dorsum: Head capsule and prothoracic shield bright orange with black dots disposed in rows, T1-T3 and A-1 segments black with white spots, T2 enlarged, 4 middle segments orange with black and white spots, caudal segments black with white spots. The lateral color pattern is the same as the dorsal color pattern.

###### Parasitoids.

Hymenoptera: Braconidae: Microgastrinae: *Glyptapanteles* Whitfield58 (n = 3); Diptera: Tachinidae: *Hemisturmia* Wood02 (n = 1).

##### Ethmia perpulchra

Taxon classificationAnimaliaLepidopteraDepressariidae

Walsingham

Figures 6 , 51 , 96 , 149

Ethmia perpulchra Walsingham, 1912: 146; Powell 1973: 132.

###### Diagnosis.

Similar externally to species matching the Exornata species-group FW pattern, but coppery blotch restricted to posterior area of FW.

###### Description.

Male: FW length: 9.5–9.8 mm (n = 3). **Head:** Front smooth, dark brown reflecting metallic blue; crown white. **Thorax:** Dorsal scaling white; paired, conspicuous dark metallic blue spots at bases of tegulae, between tegulae and on scutellum. FW ground color white, a well-defined reddish coppery blotch in posterior half from before middle to end of cell, termen broadly golden ochreous. HW with costal hair pencil elongate, gray, enclosed in a subcostal fold. **Abdomen:** Brown, genital scaling bright ochreous. Genitalia (Fig. 51) with uncus broad; gnathos broad and dentate posteriorly; basal processes moderately broad and sclerotized; valva broad, with costa developed into a large notch.

Female: FW length 11.7–12.0 mm (n = 3). **Head and thorax:** As described for male. **Abdomen:** Genitalia (Fig. 96) with papillae anales sclerotized; anterior apophyses short; sterigma broad with elongate, pointed lateral lobes; ductus basally with a dentate patch; signum narrow, dentate.

###### Holotype.

Female: Mexico, Veracruz, Orizaba, [no date], W. Schaus. USNM Type No. 68205 [USNM, examined].

###### Distribution and biology.

*Ethmia perpulchra* has been collected from Mexico (Veracruz) to Guatemala (Cayuga), Honduras (Lancetilla, Tela) and Costa Rica. In Costa Rica (Fig. 149), it has been found on the Caribbean side of the Cordillera Volcánica de Guanacaste and Tilarán from 700 to 950 m. *Ethmia perpulchra* occurs in ACG rain forest. Food plants and immature stages are unknown.

##### Ethmia terpnota

Taxon classificationAnimaliaLepidopteraDepressariidae

Walsingham

Figures 7 , 52 , 97 , 150

Ethmia terpnota Walsingham, 1912: 147, pl. 5, fig. 11; Powell 1973: 130.

###### Diagnosis.

*Ethmia terpnota* is most similar to *Ethmia millerorum* and can be discriminated on the basis of the male and female genitalia. In the male the sacculus has a finger-like projection and the valva has a smaller distal notch.

###### Description.

Male: FW length 10.9–11.7 mm (n = 3). Genitalia (Fig. 52) with uncus deeply notched, gnathos dentated anteriorly in two groups, sacculus with finger-like projection, valva with small distal notch.

Female: FW length 11.7–12.4 mm (n = 3). Genitalia (Fig. 97) with lobes of sterigma small, bowl-like with fine spines inside; ductus bursae sclerotized at base; corpus bursae with a subtle constriction in middle; signum a single row, dentated.

###### Holotype.

Male: Costa Rica, Volcan de Irazú, 6000–7000 ft. [no date] [BMNH, examined].

###### Distribution and biology.

*Ethmia terpnota* has been collected in Costa Rica (Fig. 150) in middle elevations (650 to 1800 m) on both slopes of Cordillera Volcánica de Guanacaste, Tilarán, Cordillera Volcánica Central and Talamanca. It occurs in ACG rain forest. Food plant and immature stages are unknown.

##### Ethmia millerorum

Taxon classificationAnimaliaLepidopteraDepressariidae

Phillips sp. n.

http://zoobank.org/3AE53BE0-9FD1-4CBD-A88E-9331963839F4

Figures 8 , 53 , 98 , 138 , 150

###### Diagnosis.

This species is most similar to *Ethmia terpnota*. It can be distinguished mainly on the basis of the male and female genitalia. In the male, the sacculus is slightly elevated but without the finger-like projection present in *Ethmia terpnota*. The valva distal notch is large compared with that of *Ethmia terpnota*. The corpus bursae of the female is a simple rounded sac without the constriction present in *Ethmia terpnota*. The signum is dentate with teeth slightly thinner and longer than those of *Ethmia terpnota*.

###### Description.

Male: FW length 10.9–11.9 mm (n = 3). **Head:** Labial palpus whitish curved, reaching antenna base, II and III segment brownish exteriorly, proboscis and front brownish, crown white. **Thorax:** Dorsal scaling white, dark narrow band at base of tegula and anterior pronotum, lateral black-blue spots on scutellum. FW ground color white, a large quadrate dorsal purplish coppery blotch at posterior half from 0.5× from base nearly to tornus, extending anteriorly to middle of cell, costal area gray from base to apex; costal area at base with four blackish spots, one distinct spot at dorsum. HW ground color whitish darker towards margin; costa with long, pale ochreous hair pencil enclosed in subcostal pinch-fold. **Abdomen:** Dorsum and ventral scaling brown, genital scaling ochreous. Genitalia (Fig. 53) with uncus long, notched; gnathos with posterior teeth continuous; basal processes narrow and elongate; sacculus projected anteriorly.

Female: FW length 11.4–13.9 mm (n = 3). **Head and thorax:** As described for male, HW unmodified. **Abdomen:** Genitalia (Fig. 98) with corpus bursae a simple rounded sac; signum dentate with thin and long teeth.

###### Holotype.

Male: 09-SRNP-36206, DNA Barcoded, **Costa Rica:** Guanacaste: Área de Conservación Guanacaste: Sector Cacao, Sendero Arenales, 1080m, 1M 10 Jul 2009, Harry Ramírez. Reared from *Bourreria costaricensis* (Boraginaceae). Deposited in INBio**. Paratypes: Costa Rica:** Alajuela: ACG: Sector Rincón Rain Forest, Albergue Oscar, 719 m, 1M 1.iv.2011, H. Cambronero & S. Ríos. Guanacaste: ACG: Sector Cacao, Sendero Arenales, 1080 m, 1M 2F 12.vii.2009, Harry Ramírez; Sendero Derrumbe, 1220 m, 4M 1F 28.iv/3.v.2004, H. Ramírez, 1M 1F 25.iv/1.v. 2008, M. Pereira, 1F 10.vii.2009, H. Ramírez; Derrumbe, 1310 m, 1F 14.v.2010, F Quesada & S. Ríos; Estación Cacao 1150 m, 1M 02.i.2007, D. García, 1M 6.v.2007, M. Pereira, 1F 28.iv.2007, H. Ramírez, 1M 1F 3/5.v.2008, H. Ramírez, 1F 23vii. 2009, R. Franco & S. Ríos, 14.v.2010, F. Quesada & S, Ríos; Toma de agua, 1600 m, 1F 8.x.2010, S. Ríos & F. Quesada (BMNH, INBio, EME, USNM).

###### Distribution and biology.

*Ethmia millerorum* has been found in Costa Rica (Fig. 150) from 1150 to 1300 m in the Cordillera Volcánica de Guanacaste, in ACG rain forest.

###### Food plant records.

*Ethmia millerorum* has been reared from Boraginaceae: *Bourreria costaricensis*.

###### Immature stages

(Fig. 138). Dorsum: Head and segments T1-T3 bright orange with conspicuous black dots dorsum and lateral. Abdomen segments A1-A7 dorsum white with black dots, A7–A10 bright orange with black dots.

###### Etymology.

*Ethmia millerorum* is named in honor of Kenton and Sue Miller for their lifetime generation, coaching, cheerleading, and production of conservation of global biodiversity, beginning with the recommendation for the establishment and early growth of the seed of ACG as Parque Nacional Santa Rosa in 1971.

##### Ethmia elutella

Taxon classificationAnimaliaLepidopteraDepressariidae

Busck

Figures 9 , 54 , 99 , 151

Ethmia elutella Busck, 1914: 35; Powell 1973: 133.

###### Diagnosis.

*Ethmia elutella* is most similar to *Ethmia janzeni*, and can be distinguished by its dark gray HW costal hair pencil, which is ochreous in *Ethmia janzeni*.

###### Description.

Male: FW length 7.2–7.8 mm (n = 3). **Head:** Front brownish, crown whitish. **Thorax:** Collar and tegula white, notum bluish, lateral white. FW costal half grayish, the dorsal area with a large purplish blotch concolorous with termen. HW costal pinch-fold present; dark gray hair pencil present. **Abdomen:** Brownish, genital scaling whitish. Genitalia (Fig. 54) with uncus broad hood like, gnathos dentate, basal process broad, valva with small notch distally and apical and subapical small spines.

Female: FW length 7.9–8.2 mm (n = 3). **Head and thorax:** As described for male, HW unmodified. **Abdomen:** Genitalia (Fig. 99) with sterigma a narrow band; ostium enclosed by an assymetrically bilobed fig; signum a fold with dentate margin.

###### Holotype.

Female: Panamá, Porto Bello, March 1911, A. Busck [USNM, examined].

###### Distribution and biology.

*Ethmia elutella* has been reported from Panamá (Barro Colorado Island) to Venezuela (Rancho Grande, Aragua) and Trinidad (Powell 1973). In Costa Rica (Fig. 151) has been collected from 25 to 650 m in both slopes of the Cordillera Volcánica de Guanacaste, lowlands of Sarapiquí and lowlands of Central Pacific Costa Rica. It has been found in the dry forest of ACG. The food plant and immature stages are unknown.

##### Ethmia janzeni

Taxon classificationAnimaliaLepidopteraDepressariidae

Powell

Figures 10 , 55 , 100 , 151

Ethmia janzeni Powell, 1973: 134-135.

###### Diagnosis.

*Ethmia janzeni* is most similar to *Ethmia elutella*. It can be distinguished by the HW costal hair pencil, which is ochreous in *Ethmia janzeni* and gray in *Ethmia elutella*.

###### Description.

Male: FW length 7.1–7.8 mm (n = 3). **Head:** Front brownish, crown whitish. **Thorax:** Collar and tegula white, notum bluish, lateral white. FW costal half grayish, the dorsal area with a large purplish blotch concolorous with termen. HW costal pinch-fold, ochreous hair pencil. **Abdomen:** Brownish, genital scaling whitish. Genitalia (Fig. 55) with uncus broad hood like; gnathos dentate; basal process broad, valva without small notch distally and apical spine.

Female: FW length 7.9–8.2 mm (n = 3). **Head and thorax:** As described for male, HW unmodified. **Abdomen:** Genitalia (Fig. 100) with sterigma as a narrow band; signum a fold without dentate margin.

###### Holotype.

Male and **Allotype** female: Mexico, Temescal, Oaxaca, December 12 and 21, 1963, D. H. Janzen [EME, examined].

###### Distribution and biology.

*Ethmia janzeni* has been collected from Mexico to San Salvador (Powell 1973), and in northwestern Costa Rica (Fig. 151) it is found in both slopes of the Cordillera Volcánica de Guanacaste and Península de Nicoya from 20 to 830 m. It has been found in both ACG dry and rain forest. The food plant and immature stages are unknown.

##### Ethmia ungulatella

Taxon classificationAnimaliaLepidopteraDepressariidae

Busck

Figures 11 , 56 , 101 , 152

Ethmia ungulatella Busck, 1914: 34; Powell 1973: 139.

###### Diagnosis.

*Ethmia ungulatella* can be distinguished from *Ethmia elutella* and *Ethmia janzeni* by the ochreous genital scaling of *Ethmia ungulatella*.

###### Description.

Male: FW length 8.3–9.7 mm (n = 3). **Head:** Proboscis white, front dark gray, crown white. **Thorax:** White, tegula gray basally, pronotum with 2 pairs of large dark gray spots. FW ground color white, blue-gray round markings, a conspicuous pair at the base in the posterior half. A large rounded purplish coppery blotch at posterior half from 0.5× from base nearly to tornus. HW whitish, costal fold with a dense group of whitish scales. **Abdomen:** Brown, genital scaling ochreous. Genitalia (Fig. 56) with uncus broad hood-like; gnathos dentate from base; basal process broad; valva without small notch distally, concave.

Female: FW length 9.5–10.1 mm (n = 3). **Head and thorax:** As described for male, HW unmodified. **Abdomen:** Genitalia (Fig. 101) with sterigma bearing lateral lobes; signum, an elongate fold without dentate margin.

###### Holotype.

Female: Panamá, Cabima, May 1911, A. Busck. USNM Type No. 16696 [USNM, examined].

###### Distribution and biology.

*Ethmia ungulatella* has been collected from eastern Mexico (Tamaulipas) to Panamá. In Costa Rica (Fig. 152) it has been found in the lowlands of Pacific and Caribbean slopes ranging up to 600 m. *Ethmia ungulatella* is a common species in ACG dry and rain forest. The food plant and immature stages are unknown.

#### Exornata species-group

**Figures 12–18.**
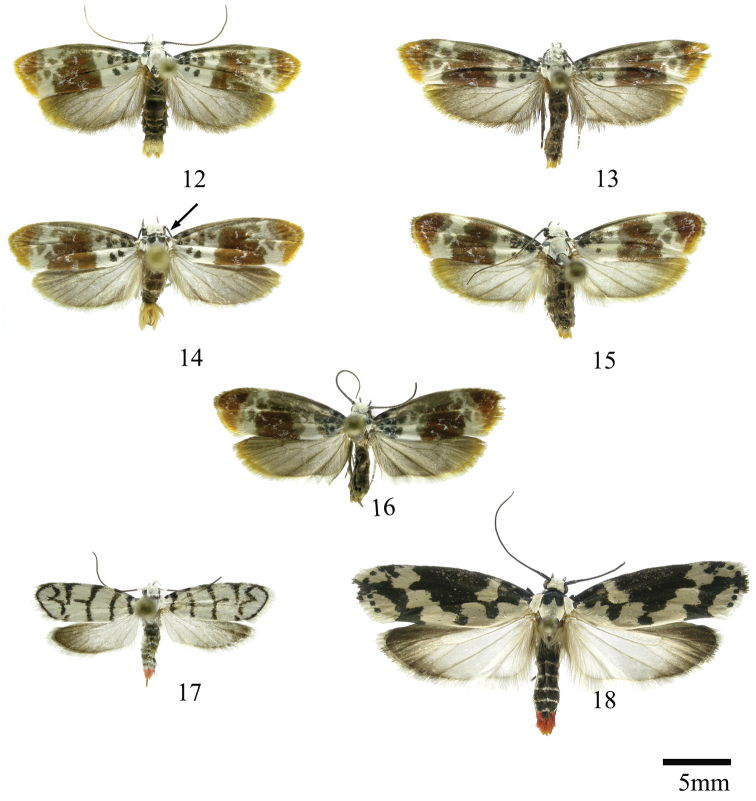
*Ethmia* from Costa Rica, adults: **12** *Ethmia exornata*, male, 08-SRNP-104410 **13** *Ethmia dianemillerae*, holotype, female, 06-SRNP-108063 **14** *Ethmia adrianforsythi*, paratype, male, INBIOCRI002138328 **15** *Ethmia phylacis*, female, 06-SRNP-104311 **16** *Ethmia mnesicosma*, male, 09-SRNP-105559 **17** *Ethmia chemsaki*, male, 06-SRNP-104292 **18** *Ethmia stephenrumseyi*, paratype, male, 10-SRNP-112265.

This is a group of species characterized by having a broad FW with a well-defined coppery blotch from posterior to near costal area, the terminal band is bright ochreous in all the species (Figs 12–16). The HW is simple and unmodified. In the abdomen the genital scaling ranges from pale to bright ochreous. The uncus is narrow, simple or with lateral arms, and the gnathos is dentate posteriorly (Figs 57–61). The anterior apophyses are short and the sterigma is simple or ornate with lateral lobes, the antrum is sclerotized in three of the species, with or without inner spurs and the signum is a narrow and dentate fold (Figs 102–106). Five species occur in Costa Rica, two described as new here.

#### Exornata species complex

Powell (1973) mentioned that the variation presented in the specimens he assigned to *Ethmia exornata* suggested that more than a single species might be involved. Through the DNA barcoding of specimens from Costa Rica, we were able to discriminate five species within this species complex: *Ethmia exornata*, *Ethmia phylacis*, *Ethmia mnesicosma* and two new species: *Ethmia dianemillerae* and *Ethmia adrianforsythi*. This species complex is depicted as one of the shaded branches in Fig. 169. Morphological traits confirm this splitting.

##### Ethmia exornata

Taxon classificationAnimaliaLepidopteraDepressariidae

(Zeller)

Figures 12 , 57 , 102 , 153

Psecadia exornata Zeller, 1877: 238 [in part]
Ethmia exornata Walsingham, 1897: 90; Powell 1973: 144.

###### Diagnosis.

*Ethmia exornata* is distinguished from *Ethmia dianemillerae*, its most similar species, by the two different sized spots at the base of FW, with the basal spot always smaller. *Ethmia exornata* possesses a much shorter basal process in male genitalia than that of *Ethmia dianemillerae*.

###### Description.

Male: FW length 9.2–10.2 mm (n = 3). **Head:** Labial palpus curved elongate exceeding base of antenna, smooth, white, base of second segment brownish, base of antenna white with dark spot; proboscis brownish, front and crown white. **Thorax:** Dorsal scaling white with dark gray markings narrow in the base of collar broad on base of tegula and a large spot on scutellum sometimes split in two longitudinally by white; underside light brown, thorax and abdomen white laterally. FW ground color white, several dark spots in costal half, two dark spots at base of posterior half, the one near base smaller than the other; a light brown band from base to 0.75× of FW, base of costa white, split by dark spot. A large reddish spot from gray band near costa to posterior, covering all area ante-medial to post-medial, a whitish zigzag line over it; termen reddish at apex, blending to golden above tornus; two dark spots in the white postmedium area beyond cell. HW costa area simple, ground color brownish with termen ochreous. **Abdomen:** Dorsal scaling brownish, white lateral, genital scaling mainly ochreous laterally, paler dorsal and ventral. Genitalia (Fig. 57) with uncus bearing lateral arms basally; gnathos bifurcated and with fine spines interior and posteriorly, base of bifurcation concave posteriorly; basal processes 0.9× length of uncus; valva apex pointed, notched at 0.6× from base length; sacculus short 0.33× length of valva.

Female: FW length 11–11.5 mm (n = 3). **Head and thorax:** As described for male. **Abdomen:** Genitalia (Fig. 102) with sterigma bearing lateral rounded lobes; ductus bursae sclerotized and slightly enlarged basally; signum a narrow fold with short teeth.

###### Holotype.

Male: Perú, Chanchamajo [MNHU].

###### Distribution and biology.

According to Powell (1973) and keeping the *Ethmia exornata* assemblage under a single name, the distribution ranges from northwestern Mexico to Brazil. Additional studies on *Ethmia exornata* from Mexico and South America are needed to assess with certainty the distribution of the species. In Costa Rica (Fig. 153), *Ethmia exornata* occurs on both Caribbean and Pacific slopes up to 1150 m. It has been found in ACG rain forest. The food plant and immature stages are unknown.

##### Ethmia dianemillerae

Taxon classificationAnimaliaLepidopteraDepressariidae

Phillips sp. n.

http://zoobank.org/8FD8759A-C366-4F78-8FBE-0D390B55E963

Figures 13 , 58 , 103 , 139 , 154

###### Diagnosis.

*Ethmia dianemillerae* is most similar to *Ethmia exornata* and can be distinguished externally by FW with two basal spots, which are similar in size, in contrast with *Ethmia exornata* where the internal dorsal spot is always smaller than the external. In the male genitalia in *Ethmia dianemillerae* the basal process is 1.3 the size of the uncus while in *Ethmia exornata* the basal process is 0.9 length of the uncus. In the females the base of the ductus bursae in *Ethmia exornata* is sclerotized and slightly augmented while in *Ethmia dianemillerae* it is simple.

###### Description.

Male: FW length: 10.3 mm (n = 1). **Head:** labial palpus curved elongate exceeding base of antenna, smooth scale white, base of second segment brownish. Base of antenna dark with white spot; proboscis brownish, front and crown white. **Thorax:** Dorsal scaling white with dark gray markings narrow in the base of collar, broad on base of tegula and a large spot on scutellum sometimes split in two longitudinally by white; underside light brown, thorax and abdomen white laterally. FW ground color white, several dark spots in costal half, two dark spots of similar size in base of posterior half, light brown band from base of costa to 0.75× of FW, base of costa dark split by white spot. A large reddish spot surrounded by brown from gray costal band to posterior margin, covering all area antemedial to postmedial, this blotch with zigzag whitish line over it; termen reddish, golden apically to above termen, two large spots in white subterminal area. HW ground color brownish with termen ochreus; costal area simple. **Abdomen:** Brownish, white lateral, genital scaling ochreous laterally, paler dorsal and ventral. Genitalia (Fig. 58) with uncus with lateral arms, gnathos bifurcated with stout spines and middle of bifurcation flat. Basal process 1.3× length of uncus; valva apex pointed, notched at 0.6× of valva length; sacculus short representing 0.45× of valva length.

Female: FW length 11.5–12.5 mm (n = 3). **Head and thorax:** As described for male. **Abdomen:** Genitalia (Fig. 103) with sterigma with lateral lobes triangular; ductus bursae slightly sclerotized basally, signum a narrow crease with short teeth. VIII segment with sclerotized rounded patches at dorsum.

###### Holotype.

Male: 06-SRNP-108063, DNA Barcoded, **Costa Rica:** Guanacaste: ACG: Sector del Oro, Lote Serrano, 585 m, 22.x.2006, H. Cambronero & R. Franco. Deposited in INBio. **Paratypes: Costa Rica:** Alajuela: ACG, Sector Rincón Rainforest, Estación Caribe, 391 m, 1F 13.vii.2007, S. Ríos & H. Cambronero; Sector San Cristóbal, Camino Brasilia, 500 m, 1F 3.ix.2008, Carolina Cano. Guanacaste: ACG, Sector del Oro, Lote Serrano, 585 m, 1F 22.x.2006, H. Cambronero & R. R.Franco. Limón: Pococí, Finca Bosque lluvioso 300 m, 1M 26.ix.2000, G. Rodríguez (INBio, EME, USNM).

###### Distribution and biology.

*Ethmia dianemillerae* has been collected in Costa Rica (Fig. 154) at middle elevations on the Caribbean slope of Cordillera Volcánica de Guanacaste, Cordillera Volcánica Central, and in the lowlands of Northern Caribbean. It has been found in ACG rain forest.

###### Food plant records.

*Ethmia dianemillerae* has been reared from larvae feeding on Boraginaceae: *Cordia alliodora* (Ruiz & Pav.) Oken.

###### Immature stages

(Fig. 139). Dorsum: Head capsule black with whitish band; prothoracic shield yellow with medium and small black dots. T2 white anteriorly with large black dots, yellow posteriorly; three evenly spaced dark spots from T3 to A3; A6, A7 and A9 white with two broad black bands. A1-A2, A4-A5 mostly yellow with white and black bands. Lateral: blackish with irregular yellow and white marks.

###### Etymology.

*Ethmia dianemillerae* is named in honor of Diane Miller of Charlottesville, Virginia, for her steering of the Blue Moon Fund in the footsteps of the W. Alton Jones Foundation, both of which allowed ACG and the Guanacaste Dry Forest Conservation Fund to acquire major blocks of Costa Rican rain forest for permanent conservation.

##### Ethmia adrianforsythi

Taxon classificationAnimaliaLepidopteraDepressariidae

Phillips sp. n.

http://zoobank.org/48A7BF78-6C0B-449B-8A76-3714E9E74A13

Figures 14 , 59 , 104 , 154

###### Diagnosis.

*Ethmia adrianforsythi* is most similar to *Ethmia exornata* and *Ethmia dianemillerae*. It can be easily distinguished by the presence of two anterior pronotal large dark spots adjoining bases of the tegula, a large spot on the scutellum sometimes split longitudinally by white, and the presence of just one spot in posterior half before the termen.

###### Description.

Male: FW length 10.3–11.2 mm (n = 3). **Head:** Labial palpus curved elongate exceeding base of antenna, smooth scale white, exterior of second and third segment brownish. Base of antenna dorsal white with brownish spots near flagellum; proboscis brownish, lateral rows of white scales basally, front and crown white. **Thorax:** Dorsal scaling white with dark gray markings narrow in the base of collar, broad on base of tegula, two large anterior pronotal spots adjoining bases of tegula and a large spot on scutellum sometimes split longitudinally by white; underside light brown, thorax and abdomen white laterally. FW ground color white, several dark spots in costal half, two dark spots of similar size in posterior half; a light brown band from base of costa to 0.75× of FW; base of costa dark split by white spot; a large reddish spot surrounded by brown from costal band to dorsal, covering all area antemedial to postmedial, this blotch with zigzag whitish line over it; terminal area reddish, blending to golden at apex; one large spot beyond cell. HW ground color brownish with termen pale ochreous; costal area simple. **Abdomen:** Dorsal scaling brownish, white lateral, genital scaling pale ochreous. Genitalia (Fig. 59) uncus trifurcated, arms same size and thin; gnathos elongated, posterior end reaching half of central uncus arm, posterior with fine spines; basal process short; valva anterior margin deeply concave starting at one third from apex; sacculus narrow 0.6× of valva length.

Female: FW length 11.5–12.5 mm (n = 3). **Head and thorax:** As described for male. **Abdomen:** Genitalia (Fig. 104) with sterigma simple unmodified; base of ductus without sleeve; signum a narrow and small fold with fine teeth.

###### Holotype.

Male: 10-SRNP-105021, DNA Barcoded, **Costa Rica:** Guanacaste, Rincón Rain Forest, Albergue Oscar, 725 m, 11.ii.2010, H. Cambronero & S. Ríos. Deposited in INBio. **Paratypes: Costa Rica:** Alajuela: ACG, Sector Rain Forest, Albergue Oscar, 725 m, 1M 11.ii.2010, H. Cambronero & S. Ríos; Sector Rain Forest, Manta Hugo, 491 m, 1M 13.iii.2009, F. Quesada & S. Ríos. Guanacaste: ACG, Sector Pitilla, 675 m, 1F 4.iv.2011, H. Cambronero & S. Ríos. Heredia: C.V.C, Sarapiquí, 11Km SE La Virgen, 500 m, 1M 19/21.ii.2003, D. Brenes. (BMNH, INBio, EME, USNM).

###### Distribution and biology.

*Ethmia adrianforsythi* has been collected in Costa Rica (Fig. 154) at middle elevations on Caribbean slope of Cordillera Volcánica de Guanacaste, and in the lowlands of Sarapiquí.

Food plant and immature stages are unknown. In ACG it has been found in the rain forest.

###### Etymology.

*Ethmia adrianforsythi* is named in honor of Adrian Forsyth of Washington, DC and the Blue Moon Fund of Charlottesville, Virginia for his continuous support of tropical biodiversity conservation from Monteverde to the Osa Peninsula to ACG, and for caring about dung beetles (Scarabaeidae).

##### Ethmia phylacis

Taxon classificationAnimaliaLepidopteraDepressariidae

Walsingham

Figures 15 , 60 , 105 , 155

Ethmia phylacis Walsingham, 1912: 147; Powell 1973: 146.

###### Diagnosis.

This species is easily confused with *Ethmia exornata* or *Ethmia dianemillerae* but is distinguished by having a single dark dot, rather than two, in the dorsal area near the base of the FW.

###### Description.

Male: FW length 8.7 to 9.5 mm (n = 3). **Head:** Labial palpus moderately elongate, curved, exceeding base of antenna; white, first and basal half of second segment slightly dark exteriorly; proboscis dark brown, front and crown white. **Thorax:** Dorsal scaling white, base of collar narrowly, tegula broadly, and scutellum dark brown. FW broad, ground color white, costal area 0.20× from base spotted dark brown; a single small spot of same color in posterior half; a large, median dorsal purplish mark extending through cell nearly to costa, apical area concolorous bronzy-purple; termen golden ochreous from tornus to above middle, blending with apical purplish. HW ground color whitish basally, becoming brownish before margins, ochreous at distal margins including fringe; costal area simple. **Abdomen:** Dorsal scaling dark brown; underside whitish with a median, longitudinal dark band; genital scaling pale ochreous. Genitalia (Fig. 60) uncus simple, basal processes with broad lateral flanges; gnathos heavily dentate, valva deeply notched at apex of sacculus.

Female: FW length 9.6–10.6 mm (n = 4). **Head and thorax:** As described for male. **Abdomen:** Genitalia (Fig. 105) with sterigma bearing lateral depressed lobes, antrum enlarged sclerotized with many inwardly directed spurs; ductus membranous; signum a narrow fold with numerous teeth.

###### Holotype.

Male: Mexico, stated as “Durango: Presidio” but should be Presidio de Mazatlan, Sinaloa (no date) [see Powell 1973] [BMNH, examined].

###### Distribution and biology.

According to Powell (1973) this species has been collected from southern Sonora and Sinaloa on the west coast to Veracruz and Yucatan on the east coast in Mexico. In Costa Rica *Ethmia phylacis* has been found in ACG dry forest at 300 m on the Pacific slope of the Cordillera Volcánica de Guanacaste (Fig. 155). The food plant and immature stages are unknown.

##### Ethmia mnesicosma

Taxon classificationAnimaliaLepidopteraDepressariidae

Meyrick

Figures 16 , 61 , 106 , 140 , 155

Ethmia mnesicosma Meyrick, 1924: 119; Powell 1973: 148.

###### Diagnosis.

*Ethmia mnesicosma* has a FW pattern similar to that of *Ethmia exornata*, *Ethmia dianemillerae*, *Ethmia adrianforsythi* and *Ethmia phylacis* but specimens are smaller and have the basal blue dark spotting extended over the posterior half of the base of the FW.

###### Description.

Male FW length: 7.5–8.9 (n = 4). **Head:** Labial palpus curved barely reaching base of antenna, smooth scale white, first and second segment tingled with brownish, third segment white. Base of antenna dorsum black with white spots near head. Proboscis brownish, head with white long scales. **Thorax:** Whitish with dark gray markings narrow in the base of collar, broad on base of tegula, a large spot on scutellum sometimes split in two longitudinally by white; underside light brown, white laterally. FW ground color white, several dark spots in costal and posterior half; a light brown narrow band starting 0.25× from base of costa to 0.70× of FW, base of costa white split by dark spot; a large reddish spot surrounded by brown from middle of cell to dorsal, from antemedial to postmedial line; termen reddish finishing golden, two big spots on costal and posterior half preceding termen. HW costal area simple, ground color light brownish with termen pale ochreus. **Abdomen:** Dorsal scaling brownish, white lateral, genital scaling pale ochreous. Genitalia (Fig. 61) with uncus simple, without lateral arms; gnathos large with posterior large spines; basal processes broad; anterior margin of valva nearly entire, sacculus not separated by a deep notch.

Female: FW length 8.9–9.2 mm (n = 3). **Head and thorax:** As described for male, except HW darker. **Abdomen:** Genitalia (Fig. 106) with sterigma slightly sclerotized; antrum enlarged sclerotized, with many inwardly directed spurs; signum a deep fold with a row of teeth.

**Lectotype.** Female: Costa Rica, San José, lectotype by Clarke (1965) [BMNH, examined].

###### Distribution and biology.

*Ethmia mnesicosma* has been collected from both coasts in southern Mexico, to northern Venezuela, Trinidad and in southern Brazil. In Costa Rica (Fig. 155) *Ethmia mnesicosma* has been found on both slopes of Cordillera de Guanacaste at middle elevations and on the Península de Nicoya. It has been found in ACG dry and rain forest.

###### Food plant records.

*Ethmia mnesicosma* has been reared from larvae feeding on Boraginaceae: *Cordia alliodora*.

###### Immature stages

(Fig. 139). Dorsum: Head capsule and prothoracic shield black with yellowish dots, pattern of regular blocks of yellow and white squared markings alternating every 3 to 4 segments.

**Parasitoids.** Diptera: Tachinidae: *Hemisturmia* Wood02 (n = 25), *Frontiniella* Wood01 (n = 24).

#### Notatella species-group

This is a group of species characterized by having a FW with dark metallic markings and genital scaling yellow-orange to bright red (Figs 17–18). The uncus is hoodlike membranous and the gnathos elongated and dentate posteriorly; valva with a strong sclerotized sacculus with inner projections. The anterior apophyses are broad. Two species occur in Costa Rica, one described as new here.

##### Ethmia chemsaki

Taxon classificationAnimaliaLepidopteraDepressariidae

Powell

Figures 17 , 62 , 107 , 156

Ethmia chemsaki Powell, 1959: 148.

###### Diagnosis.

*Ethmia chemsaki* is easily distinguished from other members of the genus by white forewings marked by distinct blue-black lines that are slender, more so than in related species, and by a bright red genital scaling.

###### Description.

Male: FW length 8.4–8.9 mm (n = 2). **Head:** Labial palpus very elongate, white; proboscis, front and crown white. **Thorax:** White, collar dark blue, scutellum bluish. FW ground color white, markings narrow black, three bands from costa to posterior margin: Near base, at 0.25× from base, and at middle of wing. HW ground color whitish becoming brown at apex. **Abdomen:** Brown with posterior margin of segments whitish, genital scaling red. Genitalia (Fig. 62) with uncus very broad, hoodlike; gnathos narrow, extending nearly the length of uncus; posterior margin of sacculus produced into a sclerotized projection.

Female: FW length 9.2–10.3 mm (n = 3). **Head and thorax:** As described for male. **Abdomen:** Genitalia (Fig. 107) with VIII segment heavily sclerotized with anterior apophyses broad and short; sterigma elongates anteriorly, with sclerotized lateral margins; signum a small sclerotized patch.

###### Holotype.

Male: Mexico, 34 miles south of Atlixco, Puebla, June 27, 1957, J. A. Chemsak [EME, examined].

###### Distribution and biology.

*Ethmia chemsaki* has been reported from Puebla, Mexico to northern Costa Rica where it has been collected on the Pacific slope of Cordillera Volcánica de Guanacaste from 20 to 300 m (Fig. 156) in ACG dry forest. The food plant and immatures are unknown.

##### Ethmia stephenrumseyi

Taxon classificationAnimaliaLepidopteraDepressariidae

Phillips sp. n.

http://zoobank.org/4D61F46D-9DA2-4B5A-8338-C7A29C1CFB90

Figures 18 , 63 , 108 , 156

Ethmia wellingi Powell 1973: 158 (in part).

###### Diagnosis.

*Ethmia stephenrumseyi* is most similar to *Ethmia wellingi* Powell, a species described from Mexico, and can be distinguished easily by the signum in the female genitalia, and by the bright red, rather than ochreous-reddish genital scaling in *Ethmia wellingi*.

###### Description.

Male: FW length 12.1–15.8 mm (n = 3). **Head:** Labial palpus curved exceeding base of antenna, whitish with brownish markings on II and III segment. Antennal scape elongated, 1.2× length of inter-antennal space; proboscis brownish at base, front and crown white. **Thorax:** White, collar and base of tegula black, two big dark spots adjoining collar extending into scutellum. FW ground color white, black markings well defined: At costal half, an elongated blotch from base to 0.75× extending narrowly into posterior half, this large blotch interrupted by a white patch at 0.25× from base; terminal area with a short oblique band before apex from costa to R2, and a broad oblique irregular band from middle of FW reaching posterior margin. HW ground color whitish becoming dark gray towards apex, a pinch fold between SC and R vein with grayish hair pencil. **Abdomen:** Dorsal scaling blackish, segments with posterior margin whitish; genital scaling reddish. Genitalia (Fig. 63) with uncus hoodlike, gnathos narrow elongate with apical lateral small spines; valva apex with an apical membranous lobe, a big sclerotized structure at apex; sacculus with a broad finger like projection on posterior margin at 0.7× from base.

Female: FW length 13.4–16.3 mm (n = 3). **Head and thorax:** As described by male except for unmodified HW. **Abdomen:** Genitalia (Fig. 108) with sterigma wide, sclerotized; antrum sclerotized; signum a large fold, 0.4× diameter of bursae at center, covered by small teeth and a row of long and conical teeth.

###### Holotype.

Male: 09-SRNP-106458, DNA Barcoded, **Costa Rica:** Guanacaste, Sector Cacao, Estación Cacao 1150 m, 21.vii.2009, R. Franco & S. Ríos. Deposited in INBio. **Paratypes: Costa Rica:** Alajuela: Arenal, San Ramón Est. Biol. Villa Blanca 1115 m, 1F 23.v.2009. E. Rojas; Área de Conservación Guanacaste, Sector Rain Forest, Albergue Oscar 719 m, 1F 1.iii.2011, H. Cambronero & F. Quesada. Cartago: Cordillera Volcánica Central, M.N. Guayabo 1100 m, 1F 7.v.2007, M. Moraga. Guanacaste: Sector Cacao, Estación Cacao 1150 m, 1F 21.vii.2009, R. Franco & S. Ríos; Laboratorio 1150 m, 3F 7.viii.2010, H. Cambronero & S. Ríos, 1M 4F 8.viii.2010, H. Cambronero & R. Franco; Derrumbe 1310 m, 2F 7.x.2010, S. Ríos & H. Cambronero, 1F 7.xi.2010, R. Franco & F. Quesada; Cima 1450 m, 7F 7.xii.2010, F. Quesada & S. Ríos; Toma de Agua 1160 m, 5F 8.ix.2010, 2F 8.x.2010, S. Ríos & R. Franco; Sector Pitilla, Estación Pitilla 675 m, 1F 2.iv.2011, H. Cambronero & S. Ríos (BMNH, INBio, EME, USNM).

###### Distribution and biology.

In Costa Rica (Fig. 156) *Ethmia stephenrumseyi* has been collected throughout the country from 660 to 1250 m. It is found in ACG rain forest. The food plant and immatures are unknown.

###### Etymology.

*Ethmia stephenrumseyi* is named in honor of Stephen Rumsey of Permian Global for his global actions to use forest conservation and recuperation to reverse climate alteration by humans and simultaneously increase survival of wild biodiversity, and his generous support of ACG as a concept and example of conservation through biodiversity development.

### Ethmia: Section II

In this section we treat *Ethmia* species that are characterized by the absence of a well-developed uncus and gnathos in male genitalia (Figs 64–90) and with long posterior apophyses in female genitalia (Figs 109–135). The general FW pattern of the species treated in Section II is characteristically a white or gray ground color with irregular and indistinct dark brown-gray elongated markings (Figs 19–45). There are 27 species in Costa Rica, 17 of which were previously undescribed.

#### Baliostola species-group

This group is characterized by FW pattern consisting of longitudinal dark streaks. HW of male with a costal brush and Sc-R pinch-fold. The abdomen has ochreous genital scaling; the uncus is short, hoodlike and the gnathos is absent. Papillae anales are membranous and posterior apophyses elongate; the antrum is enlarged with sclerotized band; the ductus bursae is sclerotized basally and the signum dentate. In Costa Rica this group includes one species.

##### Ethmia baliostola

Taxon classificationAnimaliaLepidopteraDepressariidae

Walsingham

**Figures 19–27.**
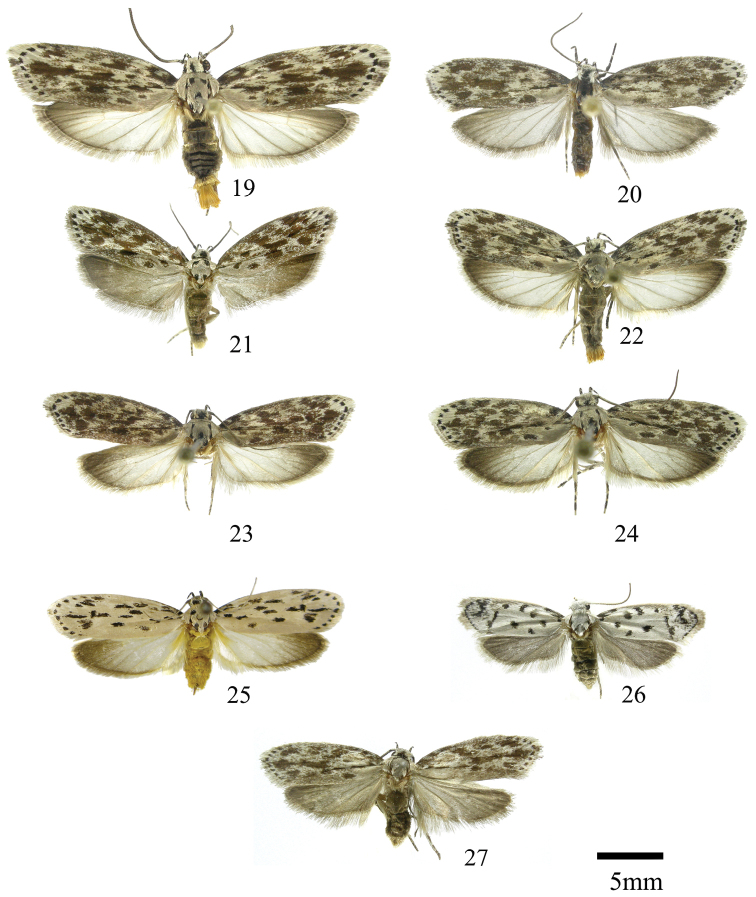
*Ethmia* from Costa Rica, adults: **19** *Ethmia baliostola*, male 11-SRNP-41492 **20** *Ethmia berndkerni*, paratype, female, 11-SRNP-102112 **21** *Ethmia dimauraorum*, paratype, male, INB0003146844 **22** *Ethmia duckworthi*, male, 09-SRNP-107349 **23** *Ethmia billalleni*, paratype, male, 10-SRNP-101550 **24** *Ethmia ehakernae*, holotype, male, 10-SRNP-102921 **25** *Ethmia sandra*, female (paratype from El Salvador, EME) **26** *Ethmia helenmillerae*, paratype, male, 11-SRNP-103927 **27** *Ethmia johnpringlei*, paratype, male, INBIOCRI00027525.

Figures 19 , 64 , 109 , 141 , 154

Ethmia baliostola Walsingham, 1912: 144.
Ethmia baliostoma Busck 1914: 54 [spelling error].

###### Diagnosis.

*Ethmia baliostola* is similar to several other gray-streaked species in Costa Rica but is distinguished externally by its large size. *Ethmia lichyi* is the only other gray species of comparable size, and can be distinguished by the number of spots on the dorsal thorax (8 in *Ethmia baliostola*, and 6 in *Ethmia lichyi*). The presence of a whitish double costal brush on the hindwing in *Ethmia baliostola* distinguishes it from other similar species.

###### Description.

Male: FW length 11.3–13.6 mm (n = 5). **Head:** Labial palpus elongated surpassing base of antenna, white scaling with blackish rings in segments II and III; proboscis, frons and crown whitish. **Thorax:** Dorsal scaling whitish, pronotum with paired blackish spots near collar, under tegula, at apices of tegula and at sides of scutellum, a single spot at middorsum. FW ground color light brown with irregular dark brown elongated markings; base of FW at posterior half without such markings; terminal line composed of ten blackish dots from before costa to tornus. HW ground color whitish becoming brownish at margin; costal brush divided at base. **Abdomen:** Dorsal scaling brown, ventral light brown. Genitalia (Fig. 64) with uncus not well defined, hoodlike, membranous; gnathos rudimentary; apex of valva produced into a lobe with a “plume” exteriorly, distal end of sacculus with row of spines of different lengths, sacculus with a protruded spine at 0.5× from base.

Female: FW length 13.6–14.3 mm (n = 4). **Head and thorax:** As described for male, except by HW not modified and the yellow genital scaling reduced. **Abdomen:** Genitalia (Fig. 109) with posterior apophyses long, sterigma simple; ductus with a long, strongly sclerotized antrum; signum a four pointed star covered with minute spines and a row of larger teeth in the longitudinal axis.

###### Holotype.

Male: Costa Rica, Banana River, March 1906, USNM Type No. 68203 [USNM, examined].

###### Distribution and biology.

This species has been recorded from southern Mexico to northwestern South America (coast of Colombia). In Costa Rica (Fig. 154) *Ethmia baliostola* has been found in the lowlands of the Caribbean (Tortuguero, Sarapiquí), on both slopes of Cordillera de Guanacaste and Cordillera de Tilarán from 600 to 1000 m and Península de Osa, from 0 to 900 m elevation. It occurs in ACG rain forest.

###### Food plant records.

*Ethmia baliostola* has been reared from larvae feeding on Boraginaceae: *Bourreria oxyphylla*, *Bourreria costaricensis*.

###### Immature stages

(Fig. 141). Dorsum: Head black, thoracic shield black-reddish anteriorly, T2 black, T3 white, A1, A2, A4, A7, A9 black with white spots evenly spaced; A3, A5, A8 white, A6 black, A10 black with middle white dot. Lateral: black with white short streaks in each segment.

###### Parasitoids.

Diptera: Tachinidae: *Hemisturmia* Janzen03 (n = 4).

#### Confusella species-group

This group is characterized by FW pattern consisting of longitudinal gray or brown/black streaks. The HW of the male is unmodified or with a hair pencil enclosed in costal fold. Scaling of the abdomen is undifferentiated, completely ochreous gray with genital scaling weakly ochreous. The uncus is membranous or absent and the gnathos absent. Valva has dense setation on inner side and cucullus “plume” is present. Papillae anales are membranous, the posterior apophyses elongate; the antrum is enlarged, usually with sclerotized band; the signum is a dentate bar, that could be reduced or lacking. In Costa Rica this group includes eight species, six described as new here.

##### Ethmia berndkerni

Taxon classificationAnimaliaLepidopteraDepressariidae

Phillips sp. n.

http://zoobank.org/505ABA35-D21F-4866-A687-D90114F2E059

Figures 20 , 65 , 110 , 158

###### Diagnosis.

*Ethmia berndkerni* is externally very similar to *Ethmia duckworthi*, and *Ethmia ehakernae*. It is distinguished by the unique shape of the basal processes and the disposition of spines on the apex of valva in male genitalia and by the smaller and unornamented signum in the female genitalia.

###### Description.

Male: FW length 7.5–9.8 mm (n = 5). **Head:** Labial palpus elongated surpassing base of antenna, black with white bands at apical half of II segment and at middle and apical of III segment; proboscis, front and crown whitish with scatter black scaling, occipital black tuft at mid-dorsum. **Thorax:** Dorsal scaling gray, collar white, narrow black line at base of tegula, paired blackish spots close to apices of tegula, dark area in scutellum divided by white, one small spot at middle thorax; underside whitish, foreleg and midleg whitish with black rings at tibia, hind leg light brown with whitish rings near tarsus. FW ground color light brown with indistinct dark brown/black elongated markings, a defined big spot at costa before apex; posterior half base without such markings; three big and distinct dark spots at posterior half 0.3× from base, one at medial area, and one above tornus; terminal line composed of eight blackish dots from before costa to tornus. HW ground color light brown becoming darker at apex; costa with short whitish modified scales on 0.5× from base, not a defined brush. **Abdomen:** Dorsal and ventral scaling light brown, genitalia scaling pale yellow to whitish. Genitalia (Fig. 65) with uncus and gnathos absent; apex of valva produced into a lobe with a “plume” with dense setation, base of this lobe with shorter setae transversely directed to setae; four long spines below apex, one pair inwardly directed, the other parallel to valve axis; a short sacculus projection below spines; basal process shaped like a sickle.

Female: FW length 8.8–10.8 mm (n = 6). **Head and thorax:** As described for male, except for unmodified HW. **Abdomen:** Genitalia (Fig. 110) with posterior apophyses long; sterigma sclerotized bilobed; antrum sclerotized and elongated, ductus bursae wide near antrum, becoming narrow anteriorly; signum small unornamented located at posterior half of corpus bursae.

###### Holotype.

Male: 11-SRNP-101561, DNA Barcoded, **Costa Rica:** Guanacaste: Sector Pitilla, Estación Pitilla, 675 m 1.iv.2011, S. Ríos & H. Cambronero. Deposited in INBio. **Paratypes: Costa Rica:** Alajuela: P.N. Volcán Tenorio, Albergue Heliconias, 700–800 m 1M 1.ii.2000, 1F 20.x.2000, 1F 7.x.2000, 4M 1F 21.ii.2001 Gladys Rodríguez. Cartago: Monumento Nal Guayabo, 1100 m, 1M 1.vii.1994, G. Fonseca. Guanacaste: Tierras Morenas, ZP Tenorio, Alto Masis 900 m, 2M 1F 1.viii.1996, 3M 1F 1.xi.1996, G. Rodríguez. 4km E Casetilla Rincón Nat. Pk. 750 m 1F 6.vi.1981, 1M 27.xii.1981 D.H. Janzen. Estación Pitilla, 9km S Sta Cecilia 700 m, 1M 20.xi.1987, 3M 1F 10/18.v.1988, 1F 1.6.1988, D.H Janzen & W. Hallwachs, 1F 10.ix.1990, 1F 1.iv.1991, 1F 1.vi.1991, 1M 1F 19.v.1993, Petrona Ríos, 1F 1.vii.1991, 1F 1.iv.1991, 1F 19.v.1993, 1M 3F 1.v.1991, 1M 27.vii.1992, 1F 19.vi.1993, 1F 1.xi.1990, C. Moraga; 1F 1.v.1989 GNP BIodiv. Inv. Sector del Oro, Sendero Manta 610 m, 1F 16.xi.2009 R.Franco & F.Quesada. Sector Pitilla, Estación Pitilla 675m, 1F 01.iv.2011 S.Ríos & H.Cambronero, 5F 2.iv.2011 H.Cambronero & S.Ríos; Sendero Memo 774 m 1F 3.iv.2011 H. Cambronero & S. Ríos. Limón: Tortuguero, Sector Cerro Cocori, Finca de E. Rojas, 150 m 1F 1.viii.1993, E. Rojas. Puntarenas: Cerro de Oro, 200m, 1F 26/30.v.1995, E. Phillips; Bosque Esquinas, 10m 2F 1.iv.1994, M. Segura, 2F 1.v.1994, J. Quesada. Corcovado Nat. Pk. 10 m, 5/14.viii.1978, D. H. Janzen. Estación Piedras Blancas, 0–100 m, 2F 12.v–16.vi.2002, M. Moraga, 1F 17.iv.2001, J. Jimenez, 1M 17.iv.2001, B. Espinoza. Los Charcos, 1 km E Banegas, 50 m 1F 6.x.2010, E. Phillips. Estación Esquinas 200 m 1F 1.iv.1993, 2M 1F 1.ix.1993, J. Quesada, 2F 1.x.1993, 1F 1.xii.1993, 1M 1.ii.1993, 2M 1.x.1993, 3M 2F 1.xi.1993, M. Segura. Fila Esquinas, 35 km S Palmar Norte, 2M 2F 7.i.1983 Janzen& Hallwachs. Rancho Quemado, 200 m 1F, 1.i.1991, 2M 2F 1.viii.1991, 2M 1.xi.1991, 4F 4M 1.xii.1991, 3M 1.ii.1992, 1M 1.iii.1992, 1M 21.iii.1992, 1F 1.vi.1992, Freddy Quesada. Río Rincón Albergue Cerro de Oro, 150 m 1F 26.v.1995, Angela Maroto (BMNH, INBio, EME, USNM).

###### Distribution and biology.

*Ethmia berndkerni* has been found in Costa Rica (Fig. 158) in the foothills of the Cordillera de Guanacaste, from 150-800 m, north Caribbean lowlands at 200 m, in middle elevations in Cordillera Volcánica Central (1000–1500 m) and Península de Osa (200 m). It occurs in ACG rain forest.

###### Food plant records.

*Ethmia berndkerni* has been reared from larvae feeding on Boraginaceae: *Bourreria costaricensis*.

###### Etymology.

*Ethmia berndkerni* is named in honor of Bernd Kern of Vaesterhaninge, Sweden for being one of the two motors, fuel and drivers of Childrens Rainforest Sweden for three decades of life blood, bone and muscle for the Eternal Childrens’ Rainforest of Monteverde, Costa Rica, and for two decades of support of ACG rain forest land purchase for permanent wildland conservation.

#### Duckworthi species complex

Through the DNA barcoding of specimens from Costa Rica we were able to initially distinguish four species within this complex: *Ethmia duckworthi* Powell (sensu stricto), and three new species: *Ethmia ehakernae*, *Ethmia billalleni* and *Ethmia dimauraorum*. This species complex is depicted as one of the shaded branches in Fig. 169. Morphology of female genitalia confirmed the species status.

##### Ethmia dimauraorum

Taxon classificationAnimaliaLepidopteraDepressariidae

Phillips sp. n.

http://zoobank.org/3131396D-F8E1-4D1C-B490-0A6CB389C252

Figures 21 , 66 , 111 , 158

###### Diagnosis.

This species is easily distinguished externally by the presence of two distinct black spots at base of FW posterior half and the presence of two anterior pronotal black spots. In male genitalia there is a characteristic row of five large flat spines on valva anterior margin.

###### Description.

Male: FW length 8.1–9.1 mm (n = 3). **Head:** Labial palpus elongated surpassing base of antenna, segment I black, segment II and III whitish with black bands apical; proboscis brownish with dispersed white scaling, front and crown whitish with scattered black scaling, occipital black tuft at mid-dorsum. **Thorax:** Dorsal scaling whitish, collar whitish, two anterior pronotal dark spots, large spot on scutellum sometimes divided by white; underside whitish, forelegs and middle legs whitish with black rings at tibia, hindlegs light brown with whitish rings near tarsus. FW ground color whitish mostly covered with irregular dark brown elongated markings except by apex. Two distinct dark brown dots on at base of FW posterior half. Terminal line composed of eight blackish dots from before costa to tornus. HW ground color brown, darker just at apex; costa with short whitish modified scales 0.3× from base, not a defined brush. **Abdomen:** Dorsal and ventral scaling light brown, genitalia scaling pale yellow. Genitalia (Fig. 66) with uncus and gnathos absent, apex of valva produced into a “plume” with elongate base; six long and flat spines below apex disposed in a row; basal process narrow and slightly curved outwards.

Female: FW length 8.3–9.1 mm (n = 6). **Head and thorax:** As described for male. **Abdomen:** Genitalia (Fig. 111) with papilla anales elongated; posterior apophyses long; sterigma sclerotized invaginated; ductus bursae sclerotized at base; signum a small scobinated patch, located at posterior half of corpus bursae.

###### Holotype.

Male: 10-SRNP-112782, DNA Barcoded, **Costa Rica:** Guanacaste: ACG: Sector Cacao, Toma de agua, 1160 m, 8.x.2010, S. Ríos & F. Quesada. Deposited in INBio. **Paratypes: Costa Rica:** Alajuela: ACG: Albergue Oscar, Manta Trocha 719 m, 1F 25.iii.2009, R. Franco & F. Quesada. Guanacaste: ACG: Sector Cacao, Derrumbe 1310 m, 4F 13.v.2010. F. Quesada & R. Franco, 1F 30.viii.2010, F. Quesada & S. Ríos, 3F 14.v.2010, R. Franco & H. Cambronero; Toma de agua, 1160 m, 1F 8.x.2010, S. Ríos & F. Quesada (BMNH, INBio, EME, USNM).

###### Distribution and biology.

*Ethmia dimauraorum* has been found in Costa Rica (Fig. 158) from 700 to 1300 m on the Pacific slope of Cordillera Volcánica de Guanacaste, at 750 m in the Cordillera Volcánica Central and at 1000 m in the Caribbean side of Cordillera de Tilarán. It occurs in ACG rain forest. The food plant and immature stages are unknown.

###### Etymology.

*Ethmia dimauraorum* is named in honor of Paul and Karen Dimaura of Boston, Massachusetts, who have generously allowed the University of Pennsylvania to support Daniel Janzen and Winnie Hallwachs full-time in their efforts to facilitate the germination and growth of ACG, INBio, and the Guanacaste Dry Forest Conservation Fund.

##### Ethmia duckworthi

Taxon classificationAnimaliaLepidopteraDepressariidae

Powell

Figures 22 , 67 , 112 , 159

Ethmia duckworthi Powell 1973: 165.

###### Diagnosis.

*Ethmia duckworthi* is most similar to *Ethmia ehakernae* and *Ethmia billalleni* and can be distinguished reliably by the large and ornamented signum in the female genitalia.

###### Description.

Male: FW length 11.9–12.8 mm (n = 3). **Head:** Labial palpus elongate, exceeding base of antenna, white with black bands. **Thorax:** Dorsal scaling gray, pronotum with three lateral pairs of large black spots: near tegula at collar, at tegula apices and at sides of scutellum, a smaller median spot preceding scutellum. FW ground color whitish, covered by irregular, dark brownish spots and longitudinal streaks, posterior area before middle and terminal area paler, the latter crossed just below apex by an elongate blotch. Two spots on paler area at base of posterior half, inner one very small, outer one distinct. HW ground color whitish basally, becoming dark brown at apical area and along costal margin; costal area simple, a pinch-fold between Sc and R, without hair pencil. **Abdomen:** Dorsal scaling brownish gray, ventral whitish, genital pale ochreous. Genitalia (Fig. 67) with uncus absent, gnathos absent, basal processes wide sinuous reaching anterior part of fultura; valva with curved spines at apex of valva.

Female: FW length 12.7 to 13.4 mm (n = 3). **Head and thorax:** As described for male. **Abdomen:** Genitalia (Fig. 112) with anterior and posterior apophyses long; sterigma narrow, slightly sclerotized; antrum sclerotized at base with anterior lateral enlargement, and three to four sclerotized patches; signum wide and long reaching 0.5 diameter of corpus bursae with large teeth disposed in rows.

###### Holotype.

Male: Panamá, Barro Colorado Island, 1–9 May 1964, W. D. & S. S. Duckworth. USNM Genitalia Slide No. 89952 [USNM, examined].

###### Distribution and biology.

This species is known from Panamá and Costa Rica. In Costa Rica (Fig. 159) *Ethmia duckworthi* occurs in both Pacific and Caribbean sides from 900 to 1450 m. It occurs in ACG rain forest. The food plant and immature stages are unknown.

##### Ethmia billalleni

Taxon classificationAnimaliaLepidopteraDepressariidae

Phillips sp. n.

http://zoobank.org/25F3F67A-0B37-41CB-8412-CAC550D39DC3

Figures 23 , 68 , 113 , 159

###### Diagnosis.

*Ethmia billalleni* its most similar externally to *Ethmia ehakernae* and *Ethmia duckworthi* and can be distinguished by its narrower FW and absence of sclerotized patches in the lateral antrum enlargement in female genitalia.

###### Description

Male: FW length 9.4–10.3 mm (n = 2). **Head:** Labial palpus curved, long, exceeding base of antenna, white with irregular black banding subapical at II segment, basal and apical at III segment; proboscis whitish, frons and crown mainly black with scatter white scaling; occipital black tuft at mid-dorsum, with lateral white tufts. **Thorax:** Dorsal scaling whitish, pronotum with three lateral pairs of large black spots: near tegula at collar, at tegula apices and at sides of scutellum, a median spot preceding scutellum; base of tegula with narrow dark band, collar whitish. FW ground color whitish, covered by irregular, dark brownish longitudinal streaks and spots except by dorsal area before middle. Area bellow apex paler crossed by an elongate blotch. HW ground color whitish basally, becoming light brown at apical area and along dorsal margin; costal area simple, without hair pencil. **Abdomen:** Dorsal scaling light brown, ventral whitish, genital scaling pale ochreous. Genitalia (Fig. 68): Uncus and gnathos absent, basal processes thin, long reaching anterior part of fultura; valva with straight spines at apex of valva.

Female: FW length 11.3 to 11.8 mm (n = 2). **Head and thorax:** As described for male. **Abdomen:** Genitalia (Fig. 113) with posterior apophyses long; sterigma narrow, slightly sclerotized; antrum sclerotized at base with lateral enlargement; signum narrow and short reaching 0.3× diameter of corpus bursae, with medium size teeth uniformly distributed in two groups.

###### Holotype.

Male: 10-SRNP-101359, DNA Barcoded. **Costa Rica:** Alajuela: ACG: Albergue Oscar, Tunel, 708 m, 1M 13.i.2010, F. Quesada & R. Franco. Deposited in INBio. **Paratypes: Costa Rica:** Alajuela: ACG: Albergue Oscar, Casa, 725 m, 1M 15.ii.2010 R. Franco & H.Cambronero; Termales 694 m, 1F 12.vi.2010, R. Franco & F. Quesada. Tunel, 708 m, 1F 13.i.2010, F. Quesada & R. Franco; La Paz Waterfall gardens, Río La Paz 1500 m, 1F 7.viii.2007, E. Phillips, B. Espinoza, A. Barrientos. Guanacaste: ACG: Sector Cacao, Derrumbe 1310 m, 1F 13.v.2010, F. Quesada & S. Ríos, Estación Cacao, 1150 m, 1F 21.vii.2009, R. Franco & S. Ríos (INBio, EME, USNM).

###### Distribution and biology.

*Ethmia billalleni* has been found in Costa Rica (Fig. 159) in middle elevations of Cordillera Volcánica de Guanacaste in ACG rain forest. The food plant and immature stages are unknown.

###### Etymology.

*Ethmia billalleni* is named in honor of Bill Allen of St. Louis, Missouri, for his accurate, detailed and continual documentation and on-site reporting on the germination and growth of ACG for almost three decades.

##### Ethmia ehakernae

Taxon classificationAnimaliaLepidopteraDepressariidae

Phillips sp. n.

http://zoobank.org/5DA9FAD9-BA0A-47CF-BA52-B095FC665F32

Figures 24 , 69 , 114 , 159

###### Diagnosis.

*Ethmia ehakernae* its most similar externally to *Ethmia billalleni* and *Ethmia duckworthi* and can be distinguished by its small, unornamented signum.

###### Description.

Male: FW length 9.1–9.8 mm (n = 3). **Head:** Labial palpus curved, long, exceeding base of antenna, white with irregular black bands basal and subapical at II and III segment; proboscis whitish, frons and crown whitish, occipital black tuft at mid-dorsum with lateral white tufts. **Thorax:** Dorsal scaling whitish, pronotum with three lateral pairs of black spots: near tegula at collar, at tegula apices and at sides of scutellum, a smaller median spot preceding scutellum; underside white, middle and hind coxa mostly white, tibia with black bands. FW ground color whitish, covered by irregular, dark brownish longitudinal streaks and spots, posterior half before middle and terminal area paler, the latter crossed just below apex by an elongate blotch. Two spots near base of posterior half, inner one very small, outer one distinct. HW ground color whitish basally, becoming brownish at apical area; costal area simple, without hair pencil. **Abdomen:** Dorsal and ventral scaling whitish, genital pale ochreous. Genitalia (Fig. 69) with uncus and gnathos absent; basal processes wide, sinuous reaching anterior margin of fultura; valva with straight spines at apex, posterior margin straight.

Female: FW length 10.2–10.8 mm (n = 3). **Head and thorax:** As described for male. **Abdomen:** Genitalia (Fig. 114) with anterior and posterior apophyses long; sterigma narrow, slightly sclerotized; antrum sclerotized at base with anterior lateral enlargement and a small sclerotized patch; signum a small and unornamented fold located close to ductus bursae junction.

###### Holotype.

Male: 10-SRNP-102921, DNA Barcoded, **Costa Rica:** Alajuela: ACG: Albergue Oscar, Casa, 725 m, 15.i.2010, R. Franco & F. Quesada. Deposited in INBio. **Paratypes: Costa Rica:** Alajuela: ACG: Albergue Oscar, Casa, 725 m, 1F 14.i.2010, F. Quesada & S. Ríos, 1F 15.i.2010 R. Franco & F. Quesada. Guanacaste: ACG: 4 km E. Casetilla Rincón Nat. Pk., 700 m 1F 25.i.1982, 1M 14.ii.1983, Janzen & Hallwachs. Rincón Nat Pk Mirador 900 m, 1M 29.iii.1984, Janzen & Hallwachs. Puntarenas: ACA: Monteverde 1300 m, 1M 20.vii.1982, Janzen & Hallwachs (INBio, EME, USNM).

###### Distribution and biology.

*Ethmia ehakernae* has been collected in Costa Rica (Fig. 159) from 700 to 1300 m in the Pacific side of Cordillera de Guanacaste and Cordillera de Tilarán. It occurs in ACG rain forest. The food plant and immature stages are unknown.

###### Etymology.

*Ethmia ehakernae* is named in honor of Eha Kern of Vaesterhaninge, Sweden, for being one of the two motors, fuel and drivers of Childrens Rainforest Sweden for three decades of life blood, bone and muscle for the Eternal Childrens’ Rainforest of Monteverde, Costa Rica, and for two decades of support of ACG rain forest land purchase for permanent wildland conservation.

##### Ethmia sandra

Taxon classificationAnimaliaLepidopteraDepressariidae

Powell

Figures 25 , 70 , 115 , 160

Ethmia sandra Powell, 1973: 166.

###### Diagnosis.

This species can be easily identified externally by the scaling of its abdomen, which is entirely ochreous, the FW pattern composed of a few short black dashes over a whitish background, and the spines on the valva being evenly spaced in male genitalia.

###### Description.

Male: FW length 10–11.4 mm (n = 2). **Head:** Labial palpus long, strongly curved; proboscis whitish, front and crown grayish, with a large black, median spot posteriorly. **Thorax:** Pale brownish; seven black spots, three pairs at sides of notum and scutellum and a small median spot preceding scutellum. FW ground color whitish with longitudinally elongated spots. HW ground color whitish, costal fold present with a white hair brush. **Abdomen:** Dorsal ochreous, venter paler; first tergum with a patch of specialized scales. Genitalia (Fig. 70) with anterior margin of valva with three evenly spaced long spines at apex.

Female: FW length 12.7 mm. **Head and thorax:** As described by male, HW unmodified. **Abdomen:** Genitalia (Fig. 115) with sterigma broad; antrum with sclerotized band; signum large, dentate.

###### Holotype.

Male: El Salvador, 13 km N San Salvador, 4 February 1965, S. S. & W. D. Duckworth [USNM, examined].

###### Distribution and biology.

*Ethmia sandra* has been recorded from Mexico to El Salvador and Costa Rica. In Costa Rica (Fig. 160) has been collected in rain forests near Turrialba, Cartago. The food plant and immature stages are unknown.

###### Remarks.

The abdomens of female specimens captured at lights are covered with pollen, suggesting that oviposition occurs into flowers of a plant that blooms in the dry season (Powell 1973).

##### Ethmia helenmillerae

Taxon classificationAnimaliaLepidopteraDepressariidae

Phillips sp. n.

http://zoobank.org/7665E892-5D57-4A61-A0DF-D3BB7BEBA101

Figures 26 , 71 , 116 , 160

###### Diagnosis.

*Ethmia helenmillerae* is distinguished by its smaller size and white forewings with spaced black markings, dark brown HW, valva in male genitalia with 2 long spines inwardly directed, and female genitalia lacking a signum.

###### Description.

Male: FW length 6.3–6.8 mm (n = 3). **Head:** Labial palpus elongated exceeding base of antenna, whitish; proboscis, front and crown white. **Thorax:** Tegula and collar white, pronotum white with four dark brown spots, one pair under tegula apices, the other at scutellum. FW ground color white, 6 defined dots distributed on 2/3 from base: three at costal area approaching costa and three at posterior half, triangulated; terminal area with a line from almost center of the wing to tornum, a wider and curved line from costa just before apex to middle of wing; terminal line black, fringe whitish. HW ground color dark brown, costal fringe pale ochreous. **Abdomen:** Dark brown, genital scaling pale. Genitalia (Fig. 71) with uncus and gnathos absent, apex of valva produced into a lobe with a “plume” with dense setation, base of plume with shorter setae transversely directed to setae at plume; a group of seven long spines bellow apex, two inwardly directed; a short pointed sacculus projection below spines.

Female: FW length 6.5–7.5 mm (n = 2). **Head and thorax:** As described for male. **Abdomen:** Genitalia (Fig. 116) with sterigma a simple band lightly sclerotized; antrum laterally enlarged; corpus bursae a simple sac with no signum.

###### Holotype.

Male: 11-SRNP-103265, DNA Barcoded, **Costa Rica:** Alajuela: ACG: Albergue Oscar, Casa, 725 m, 15.i.2010, R. Franco & F. Quesada. Deposited in INBio. **Paratypes: Costa Rica:** Guanacaste: Sector Maritza, Casa Rafa 579 m, 1F 1.vi.2011, R. Franco & S. Ríos; Sector Santa Elena, La Angostura, 2F 25.v.2009, F. Quesada & R. Franco, Manta Potrero Grande 20 m, 1F 22.v.2009, F. Quesada & H. Cambronero, 1F 24.v.2009, F. Quesada & R. Franco, 4F 21/22.viii.2009, F. Quesada & R. Franco; Sector Santa Rosa, Luces 575 m, 3F 30.v/1.vi.2011, R. Franco & S. Ríos, 2F 31.v.2011, H. Cambronero & S. Ríos, 5F 1.vi.2011, H. Cambronero & R. Franco, Mirador Río Cuajiniquil, 242 m, 1M 3F 23.vi.2009, R. Franco & F. Quesada, 1F 23.vi.2009, H. Cambronero & R. Franco (BMNH, INBio, EME, USNM).

###### Distribution and biology.

*Ethmia helenmillerae* has been collected in ACG dry forest on the Pacific slope of Cordillera Volcánica de Guanacaste from 0 to 579 m. The food plant and immature stages are unknown

###### Etymology.

*Ethmia helenmillerae* is named in honor of Helen Miller of Arlington, Virginia, an early major donor for ACG rain forest purchase, and her production of Scott Miller, Smithsonian Institution, Washington, DC, a faithful supporter of ACG inventory for biodiversity development.

##### Ethmia johnpringlei

Taxon classificationAnimaliaLepidopteraDepressariidae

Phillips sp. n.

http://zoobank.org/247A9873-10E1-478A-8D89-2FE77E58D686

Figures 27 , 72 , 117 , 160

###### Diagnosis.

This species can be distinguished by its smaller size and the presence of three curved large distal setae on the valva of male genitalia.

###### Description.

Male: FW length 7.1–7.8 mm (n = 3). **Head:** Labial palpus elongated surpassing base of antenna, grayish with black rings in II segment middle and subapical, a wide black ring subapical in III segment; front and crown grayish, occipital black tuft at mid-dorsum. **Thorax:** Grayish. FW ground color grayish, with a series of elongated dark markings, the most conspicuous an oblique line originating at costa before middle reaching a longitudinal line that goes from middle to near termen; three irregular black dots on posterior half: one at 0.20× from base, one close to midline and third near tornus. HW ground color white, costa simple. **Abdomen:** Brownish, genital scaling pale ochreous. Genitalia (Fig. 72) with uncus lacking; valva emarginate bellow apex, with apical “plume”, and distally three curved, strong, large setae.

Female: FW length 7.9–8.3 mm (n = 3). **Head and thorax:** As described for male. **Abdomen:** Genitalia (Fig. 117) with sterigma simple; base of ductus sclerotized; antrum membranous; signum a short fold with irregular margin.

###### Holotype.

Male: 11-SRNP-104923, DNA Barcoded, **Costa Rica:** Guanacaste: ACG, Sector Maritza, Manta Mecate 587 m, 1.x.2011, R. Franco & S. Ríos. Deposited in INBio. **Paratypes: Costa Rica:** Guanacaste: ACG: Sector Maritza, Manta Mecate 587 m, 1.x.2011, R. Franco & S. Ríos. Est. Maritza, Lado Oeste del Volcán Orosi, 600 m, 2M 13.v.1988 Janzen and Hallwachs, 10M 11F 1.viii.1990, II curso Parataxónomos, 17M 27F 1.vii.1990, I Curso Microlepidoptera. 4 km W. Sta. Cecilia, 300 m 1M 17.iv.1983, Janzen & Hallwachs (BMNH, INBio, EME, USNM).

###### Distribution and biology.

*Ethmia johnpringlei* has been collected in northern Costa Rica on the western sides of Cordillera Volcánica de Guanacaste, from 300 to 600 m in the intersection between ACG dry forest and rain forest. The food plant and immature stages are unknown.

###### Etymology.

*Ethmia johnpringlei* is named in honor of John Pringle of Stanford University, the father of Rob Pringle of Princeton University, and a member of the very small and select group of academic faculty who have supported ACG land purchase for permanent conservation.

#### Longimaculella species-group

The FW pattern in this group consists of longitudinal blackish streaks. HW of the male has a costal brush. The genital scaling is ochreous, the uncus is membranous and the gnathos is absent; valva with or without cucullus “plume”. Papillae anales are membranous, setate and posterior apophyses are elongate; the antrum is enlarged with sclerotized band and the signum is notched. In Costa Rica this group includes 13 species, nine described as new here.

##### Ethmia nigritaenia

Taxon classificationAnimaliaLepidopteraDepressariidae

Powell

**Figures 28–40.**
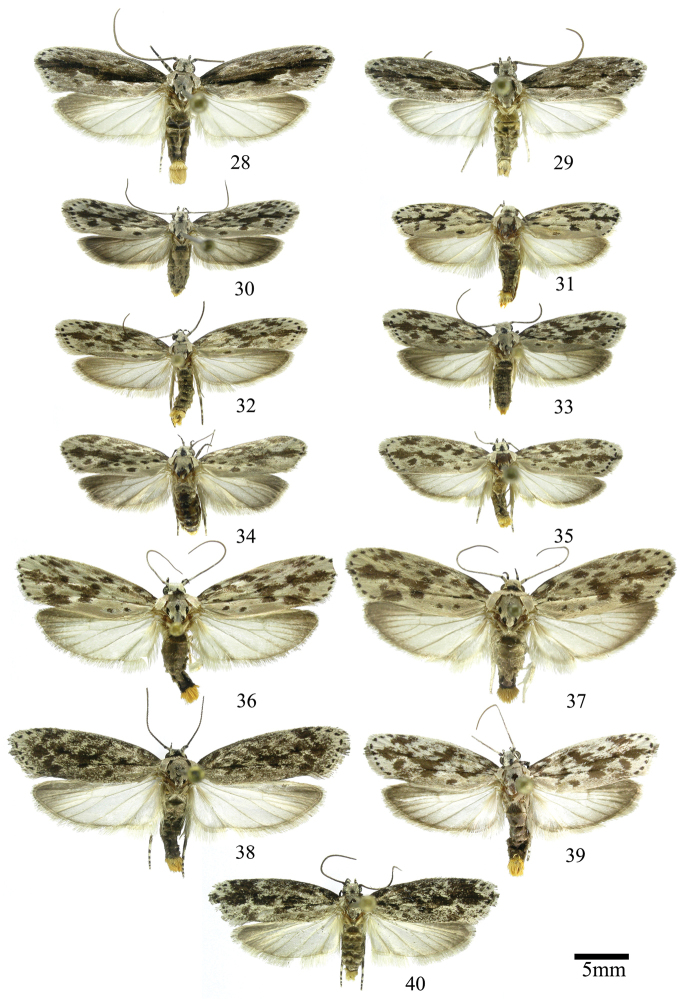
*Ethmia* from Costa Rica, adults: **28** *Ethmia nigritaenia*, male, 06-SRNP-105902 **29** *Ethmia laphamorum*, paratype, male, 09-SRNP-104303 **30** *Ethmia catapeltica*, female, 10-SRNP-42303 **31** *Ethmia petersterlingi*, holotype, male, 09-SRNP-109881 **32** *Ethmia lesliesaulae*, holotype, male, 11-SRNP-1973 **33** *Ethmia turnerorum*, paratype, female, 10-SRNP-57341 **34** *Ethmia normgershenzi*, holotype, female, 11-SRNP-30760 **35** *Ethmia nicholsonorum*, holotype, male, 09-SRNP-1051 **36** *Ethmia lichyi*, male, INBIOCRI002063133 **37** *Ethmia hendersonorum*, paratype, male, INB0004222481 **38** *Ethmia transversella*, male, INB0004263677 **39** *Ethmia randyjonesi*, paratype, male, 07-SRNP-108189 **40** *Ethmia randycurtisi*, holotype, male, INB0003101969.

Figures 28 , 73 , 118 , 161

Ethmia nigritaenia Powell, 1973: 172.

###### Diagnosis.

*Ethmia nigritaenia* is characterized by a black stripe through the middle of the FW from base to apex and can be distinguished from *Ethmia laphamorum*, its most similar species, by its larger size and by the valva with a conspicuous apical plume and a large distal spine in male genitalia.

###### Description.

Male: FW length 12.8–14.5 mm (n = 3). **Head:** Labial palpus curved, long, exceeding base of antenna, proboscis, front and crown white, occipital tufts dark brown at mid-dorsum. **Thorax:** Pronotum whitish with seven small black spots. FW whitish with indistinct light brown markings except by a median black streak from base to apex. HW whitish; broad costal fold reaching end of cell, enclosing a thick brush of elongate hair scales from base. **Abdomen:** Dark brown, genitalia scaling pale ochreous. Genitalia (Fig. 73) with uncus elongate, moderately sclerotized; valva apex with broad “plume”; sacculus with long wide projection just bellow apex.

Female: FW length 14.5–15.3 mm (n = 3). **Head and thorax:** As described for male, except HW unmodified. **Abdomen:** Genitalia (Fig. 118) with sterigma simple; base of ductus with long sclerotized sleeve; signum a large fold, emarginated at one side.

###### Holotype.

Male: Mexico, Chichen Itza, Yucatan, Feb., 1956, E. C. Welling [EME, examined].

###### Distribution and biology.

*Ethmia nigritaenia* distribution ranges from southern Mexico to northwestern Costa Rica. In Costa Rica (Fig. 161) it has been collected on the Pacific side of Cordillera Volcánica de Guanacaste from 20 to 570 m in ACG dry forest.

###### Food plants records.

*Ethmia nigritaenia* has been reared from larvae feeding on Boraginaceae: *Cordia gerascanthus* L.

##### Ethmia laphamorum

Taxon classificationAnimaliaLepidopteraDepressariidae

Phillips sp. n.

http://zoobank.org/CEA37E1E-1285-4AA0-8FD2-CBBE00802C58

Figures 29 , 74 , 119 , 161

###### Diagnosis.

*Ethmia laphamorum* can be distinguished from *Ethmia nigritaenia*, its most similar species, by the smaller size and the less contrasting line through the middle of the FW from base to apex. In the male genitalia *Ethmia laphamorum* presents a very reduced “plume” projection at the apex of the valva and the distal spines are absent. In the female genitalia, the sclerotized sleeve at the base of ductus is shorter than that of *Ethmia nigritaenia*.

###### Description.

Male: FW length 10.8–12 mm (n = 3). **Head:** Labial palpus curved, long, exceeding base of antenna; proboscis and front white with lateral brownish markings, crown light brown, occipital tuft dark brown at mid-dorsum. **Thorax:** Pronotum light brown with black spots marginal. FW brownish with irregular darker markings; posterior area with an elongated dark mark at 0.3× from base; an irregular black band from base to apex. HW whitish; costal fold to 0.7× from base, enclosing a whitish brush of elongate hair scales from base. **Abdomen:** Pale ochreous dorsal and ventral, bright ochreous tergum I and II, genitalia scaling concolorous. Genitalia (Fig. 74) with uncus sclerotized; valva apex with a very reduced “plume”, distal spines absent; a large finger like projection at sacculus, below apex.

Female: FW length 11.1–12.7 mm (n = 3). **Head and thorax:** As described for male, except HW unmodified. **Abdomen:** Genitalia (Fig. 119) with sclerotized sleeve at base of ductus bursae; signum with posterior margin serrated.

###### Holotype.

Male: 09-SRNP-104663, DNA Barcoded, **Costa Rica:** Guanacaste, Sector Santa Elena La Angostura 300 m, 25.v.2009, F. Quesada & R. Franco. Deposited in INBio. **Paratypes: Costa Rica:** Guanacaste: Sector Maritza, Casa Rafa 579 m, 2M 1F 4/5.vi.2011, H. Cambronero & R. Franco; Sector Murciélago, Mirador Murciélago 87 m, 2M 6F 22.xi.2011, H. Cambronero & R. Franco, 5F 23.xi.2011, H. Cambronero & S. Ríos, Mirador Nance 133 m, 2M 1F 23/24.xi.2011, H. Cambronero & S. Ríos, 3F 23.xi.2011, S. Ríos & R. Franco; Sector Santa Elena, La Angostura 300 m, 3M 7F 25/26,v,2009, F. Quesada & R. Franco, Manta Potrero Grande 20 m, 3F 22.v.2009, F. Quesada & H. Cambronero. Sector Santa Rosa, Luces 575 m, 3M 6F 31.v/1.vi. 2011, H. Cambronero & S. Ríos, Sendero los Patos 3M 3F 9.xii.2007, F. Quesada & R. Franco (BMNH, INBio, EME, USNM).

###### Distribution and biology.

In Costa Rica (Fig. 161), *Ethmia laphamorum* has been collected on both slopes of Cordillera Volcánica de Guanacaste and Península de Nicoya, from 20 to 600 mts. It occurs in ACG dry forest.

The food plant and immature stages are unknown.

###### Etymology.

*Ethmia laphamorum* is named in honor of Nick and Gardiner Lapham of Rappahannock, Virginia for their many years of support of ACG rain forest land purchase for permanent wildland conservation.

#### Catapeltica species complex

Powell (1973) mentioned that the variation presented in the specimens that he assigned to *Ethmia catapeltica* Meyrick suggested that more than a single species might be involved. Through the DNA barcoding of specimens from Costa Rica we were able to discriminate six species within this species complex: *Ethmia catapeltica* Meyrick (sensu stricto), and five new species: *Ethmia petersterlingi*, *Ethmia lesliesaulae*, *Ethmia turnerorum*, *Ethmia normgershenzi* and *Ethmia nicholsonorum*. This species complex is depicted as one of the shaded branches in Fig. 169. Morphology and additional information on immature stages and food plants confirmed the species status. There are conspicuous differences in larval coloration pattern (Figs 142–145).

##### Ethmia catapeltica

Taxon classificationAnimaliaLepidopteraDepressariidae

Meyrick

Figures 30 , 75 , 120 , 142 , 162

Ethmia catapeltica Meyrick, 1924: 119; Powell 1973: 174 (in part).

###### Diagnosis.

*Ethmia catapeltica* can be distinguished externally from its most similar species, *Ethmia petersterlingi*, by its general darker appearance, the presence of three distinct dark spots at base of FW posterior half, basal processes slightly wider anteriorly and sacculus slightly emarginated distally in the male genitalia.

###### Description.

Male: FW length 10.3–15 mm (n = 4). **Head:** Labial palpus elongated, exceeding base of antenna. Proboscis, front and crown whitish, occipital tuft black at mid-dorsum. **Thorax:** Ground color grayish white, four large black spots near apices of tegula and at sides of scutellum. FW ground color whitish, markings blackish, three distinct spots at posterior half: basal, middle and above torsum; an oblique dark blotch from before middle in costa connecting with an elongated black mark from middle to termen. HW ground color grayish darker at margins, a narrow costal fold with blackish long fringe at ventral, with a thin white pencil. **Abdomen:** Dorsal pale brownish, genital scaling pale. Genitalia (Fig. 75) with uncus membranous; basal processes slightly broadened apically; distal margin of valva with a small projection, adjunct to a group of three to four flat spines; distal sacculus margin slightly emarginated.

Female: FW length 13.1–15.5 mm (n = 3). **Head and thorax:** As described for male except HW unmodified. **Abdomen:** Genitalia (Fig. 120) with sterigma narrow; antrum sclerotized; signum barely notched.

###### Holotype.

Male: Costa Rica, San José [BMNH, examined].

###### Distribution and biology.

According to Powell (1973) and to keep the *Ethmia catapeltica* assemblage under a single name, the distribution ranges from Veracruz, Mexico to Bolivia. Additional studies on *Ethmia catapeltica* from Mexico and South America are needed to assess with certainty the distribution of the species. In Costa Rica (Fig. 162) *Ethmia catapeltica* (sensu stricto) occurs in the Caribbean and Pacific slopes, at both sides of Cordillera Volcánica de Guanacaste and Tilarán at middle elevations, at Sarapiquí lowlands, Central Pacific and southern Caribbean. In ACG, this is a rain forest species.

###### Food plant records.

*Ethmia catapeltica* has been reared from larvae feeding on Boraginaceae: *Cordia alliodora*.

###### Immature stages

(Fig. 142). Dorsum: Head black, thoracic shield ochreous, T2 black, T3 white, A1 and A7 white with irregular dark band, A2 white with short lateral black line, A3-A5 black, A6 black whitish posteriorly, A8-A9 black, A10 whitish with few small dots at dorsum. Lateral: Segments A3-A6 with white patches. Fully-grown larval coloration is quite similar to that of *Ethmia lesliesaulae* (Fig. 143).

###### Parasitoids.

Hymenoptera: Ichneumonidae: Lycorininae, *Lycorina luzae* Gauld (n = 10).

##### Ethmia petersterlingi

Taxon classificationAnimaliaLepidopteraDepressariidae

Phillips sp. n.

http://zoobank.org/873963D8-F9FE-4651-8908-09CAC73F804E

Figures 31 , 76 , 121 , 162

###### Diagnosis.

This species is most similar to *Ethmia catapeltica* and can be discriminated by thin FW markings, and sacculus without emargination distally in the male genitalia. The female genitalia with signum deeply notched.

###### Description.

Male: FW length 9–9.4 mm (n = 3). **Head:** Labial palpus elongated, exceeding base of antenna; proboscis brown, front and crown whitish, occipital tuft black at mid-dorsum. **Thorax:** Ground color whitish, four large black spots near apices of tegula and at sides of scutellum. FW ground color whitish, markings blackish, one distinct spot at base. An oblique, thin, dark mark from before mid costa connecting with a longitudinal blotch from middle to termen. HW ground color whitish, darker at margins, narrow costal fold with blackish long fringe at ventral, with a thin white pencil. **Abdomen:** Dorsal and ventral pale brownish, genital scaling pale. Genitalia (Fig. 76) with uncus membranous; basal processes narrow; distal margin of valva with a small projection, adjunct to a group of three or four flat spines.

Female: FW length 9.3–9.5 mm (n = 3). **Head and thorax:** As described for male except HW unmodified. **Abdomen:** Genitalia (Fig. 121) with sterigma narrow; antrum sclerotized; signum notched.

###### Holotype.

Male: 09-SRNP-109881, DNA Barcoded, **Costa Rica:** Guanacaste: ACG: Mundo Nuevo, Manta Canon 700 m, 18.x.2009 H. Cambronero & F. Quesada. Deposited in INBio. **Paratypes: Costa Rica:** Guanacaste: ACG: Sector Mundo Nuevo, Pozo #3, 634 m, 1M 16.x.2009, H. Cambronero & F. Quesada, Punta Plancha 420 m, 1F 16.vi.2010, M. Pereira; Sector Pailas, Manta Río Blanco 790 m, 1F 10.viii.2010, H. Cambronero & S. Ríos; Sector Horizontes, Bajo Sombra 181 m, 1M 24.x.2011, H. Cambronero & S. Ríos (INBio, EME, USNM).

###### Distribution and biology.

This species has been collected in Costa Rica (Fig. 162) on both sides of Cordillera Volcánica de Guanacaste (180–790 m) in ACG rain forest.

###### Food plant records.

*Ethmia petersterlingi* has been reared from larvae feeding on Boraginaceae: *Cordia alliodora*.

###### Etymology.

*Ethmia petersterlingi* is named in honor of Peter Sterling, professor emeritus of the University of Pennsylvania and resident of Panamá, for his coaching and encouragement of the early days of ACG growth, and for his support of the blossoming of school children biodiversity education in Panamá.

##### Ethmia lesliesaulae

Taxon classificationAnimaliaLepidopteraDepressariidae

Phillips sp. n.

http://zoobank.org/86A16448-C449-4660-B769-3122B50D0D5D

Figures 32 , 77 , 122 , 143 , 163

###### Diagnosis.

*Ethmia lesliesaulae* can be distinguished from *Ethmia turnerorum*, its most similar species, by a heavier marked, pre-medial diagonal band in the FW. In the male genitalia there is a small projection at 0.3× of “plume” base, and distal margin of sacculus slightly emarginated; signum without a notch in the female genitalia.

###### Description.

Male: FW length 8.4–10.3 mm (n = 3). **Head:** Labial palpus elongated, reaching collar anteriorly, white with dark rings medial and subapical in II segment, basal and subapical in III segment; proboscis, front and crown white, occipital tuft black at mid-dorsum. **Thorax:** Ground color whitish, four large black spots near apices of tegula and at sides of scutellum. FW ground color whitish, markings blackish, two distinct spots at base, inner one smaller; an oblique dark blotch from before middle in costa connecting with an elongated mark dark from middle to termen. HW ground color whitish, darker at margins, costal fold narrow with blackish long fringe and thin white pencil. **Abdomen:** Dorsal pale brownish, genital scaling pale ochreous. Genitalia (Fig. 77) uncus membranous; basal processes narrow, broadened basally; distal margin of valva with four flat spines; small projection posteriorly at 0.3× of “plume” base; sacculus narrow, emarginated at 0.6× from base.

Female: FW length 8.9–10.4 mm (n = 3). **Head and thorax:** As described for male except HW unmodified. **Abdomen:** Genitalia (Fig. 122) with sterigma narrow; antrum sclerotized; posterior base of ductus enlarged; signum without a notch.

###### Holotype.

Male: 11-SRNP-1973, DNA Barcoded, **Costa Rica:** Guanacaste, Área de Conservación Guanacaste, Sector San Cristobal, Tajo Angeles 540 m, 13.v.2011, Elda Araya. Reared on *Drymonia macrophilla* (Gesneriaceae) (Janzen and Hallwachs 2013). Deposited in INBio. **Paratypes: Costa Rica:** Guanacaste, Sector del Oro, Quebrada Lajosa 400m, 1M 9.xii.2004, Lucia Ríos, Sendero Puertas 400 m, 1 F 4.i.2010, L. Ríos; Sector Pitilla, Pasmompa 440 m, 1M 1F 7/19.xii.2004, C. Moraga, 1M 3.viii.2005, L. Ríos; Sector San Cristobal, 527 m, 9.xi.2009, Gloria Sihezar (INBio, EME, USNM).

###### Distribution and biology.

This species has been collected in Costa Rica (Fig. 163) at both sides of Cordillera Volcánica de Guanacaste from 300 to 645 m, in ACG rain forest.

###### Food plant records.

*Ethmia lesliesaulae* has been reared from larvae feeding on Gesneriaceae: *Drymonia macrophylla* (Oerst.) H. E. Moore, *Drymonia serrulata* (Jacq.) Mart., *Drymonia warszewicziana* Hanst. and *Drymonia alloplectoides*.

###### Immature stages

(Fig. 143). Dorsum: Head black, thoracic shield ochreous, T2 black, T3 white with thin and irregular dark line, A1 white with short lateral black line, A2–A5 black, A6 black whitish posteriorly, A7 white, A8-A9 black, A10 whitish with few small dots at dorsum. Lateral: As in dorsum, except by segments A3-A6 with white patches.

**Parasitoids.** Hymenoptera: Braconidae: Microgastrinae (n = 1); Eulophidae: Eulophinae: *Elachertus* Hansson03 (n = 1).

###### Etymology.

*Ethmia lesliesaulae* is named in honor of Leslie Saul of San Francisco, California, one of the two motors and electricity that invented the Conservation Parking Meter, the traveling insect zoo, and www.SaveNature.org, and has hustled for two decades on behalf of ACG rain forest land purchase for permanent wildland conservation.

##### Ethmia turnerorum

Taxon classificationAnimaliaLepidopteraDepressariidae

Phillips sp. n.

http://zoobank.org/30BB010C-4C54-4EF0-A9C0-1FE51752D104

Figures 33 , 78 , 123 , 144

###### Diagnosis.

*Ethmia turnerorum* can be distinguished from *Ethmia lesliesaulae*, its most similar species, by a thinner marked pre-medial diagonal band in the FW. In the male genitalia, sacculus narrow, slightly elevated posteriorly at 0.5× from base; signum deeply notched in female genitalia.

###### Description.

Male: FW length 10.2–10.6 mm (n = 3). **Head:** Labial palpus elongated, reaching collar anteriorly, white with dark narrow rings basal, medial and subapical in II segment, basal and subapical in III segment; proboscis, front and crown white, occipital tuft black at mid-dorsum. **Thorax:** Ground color whitish, four large black spots near apices of tegula and at sides of scutellum. FW ground color whitish, markings blackish, two distinct spots at base. An oblique irregular dark blotch from before middle bellow costa connecting with an elongated mark dark from middle to termen. HW ground color whitish darker at margins, a narrow costal fold with blackish long fringe at ventral, with a thin white pencil. **Abdomen:** Dorsal pale brownish, genital scaling pale ochreous. Genitalia (Fig. 78) with uncus membranous; basal process narrow, broadened basally; distal margin of valva with five flat curved spines; sacculus narrow, slightly elevated posteriorly at 0.5× from base.

Female: FW length 10.3–10.7 mm (n = 3). **Head and thorax:** As described for male except HW unmodified. **Abdomen:** Genitalia (Fig. 123) with sterigma narrow; antrum sclerotized; posterior base of ductus with sclerotized patch; signum deeply notched.

###### Holotype.

Male: 05-SRNP-45922, DNA barcoded, **Costa Rica:** Guanacaste, ACG, Sector Cacao, Quebrada Otilio, 550m, 16.vii.2005, Dunia García. Reared from *Cordia panamensis* (Boraginaceae) (Janzen and Hallwachs 2013). Deposited in INBio. **Paratypes: Costa Rica:** Guanacaste: ACG: Sector Cacao: Quebrada Otilio 550 m, 1F 11/16.vii.2005, D. García; Sector Del Oro, Quebrada Salazar 560 m, 1M 1F 18.xii.2006, Roster Moraga; Sector Maritza, Maritza 548 m, 1F 29.ix.2011, H. Cambronero & S. Ríos; Sector Mundo Nuevo, Punta Plancha 420 m, 1F 15.xii.2010, José Cortéz; Sector Santa María, Santa María 832 m, 1M 11.v.2012, R. Franco & F. Quesada; Sector Santa Rosa, Luces 575 m, 1M 31.v.2011, H. Cambronero & S. Ríos, Mirador Río Cuajiniquil 242 m, 1F 24.vi.2009, H. Cambronero & R. Franco (INBio, EME, USNM).

###### Distribution and biology.

This species has been collected in Costa Rica (Fig. 163) at both sides of Cordillera Volcánica de Guanacaste from 242 to 832 m in the intersection of ACG dry forest and rain forest.

###### Food plant records.

*Ethmia turnerorum* has been reared from larvae feeding on Boraginaceae: *Cordia panamensis*.

###### Immature stages

(Fig. 144). Dorsum: Head black, thoracic shield ochreous, T2 black, T3-A1 white with irregular black band in middle of each segment, A2 black and white posteriorly, A3 black, A4 black whitish posteriorly, A5 black, white posteriorly, A6-A7 white with irregular black band in middle of each segment, A8 white, black posteriorly, A9 black, A10 whitish with small black dots. Small paired black dots visible on dorsum of white segments. Lateral: As in dorsum.

###### Etymology.

*Ethmia turnerorum* is named in honor of J. D. and Nancy Turner of Ardmore, Tennessee for being insanely in love with Riodinidae butterflies and for funding the BioLep building in the ACG Administration Área in Sector Santa Rosa, the building that is the action center for the inventory and DNA barcoding of the total Lepidoptera fauna of ACG.

##### Ethmia normgershenzi

Taxon classificationAnimaliaLepidopteraDepressariidae

Phillips sp. n.

http://zoobank.org/0D868B62-A0EA-4390-BD10-0BD50BA45809

Figures 34 , 79 , 124 , 145 , 164

###### Diagnosis.

*Ethmia normgershenzi* can be distinguished from last three species by the HW costal fold with whitish border. In male genitalia *Ethmia normgershenzi* can be distinguished by the spines disposed on a row in the distal margin of the valva.

###### Description.

Male: FW length 10.6–10.9 mm (n = 3). **Head:** Labial palpus elongated, reaching collar anteriorly, white with dark irregular rings basal, medial and subapical in II segment, basal and subapical in III segment; proboscis, front and crown whitish, occipital tuft black at mid-dorsum. **Thorax:** Ground color whitish, four large black spots near apices of tegula and at sides of scutellum. FW ground color whitish, markings blackish, two distinct spots at base, inner smaller; an oblique irregular dark blotch before middle directed to but unattached to an elongated mark line from middle to termen. HW ground color whitish darker at margins, a narrow costal fold with whitish border at ventral, with a thin white pencil. **Abdomen:** Dorsal pale brownish, genital scaling pale ochreous. Genitalia (Fig. 79) with uncus membranous, basal processes narrow broadened basally; distal margin of valva with five flat spines disposed on a row; sacculus narrow, margin emarginated at 0.7× from base.

Female: FW length 10.9–11 mm (n = 2). **Head and thorax:** As described for male, except HW unmodified. **Abdomen:** Genitalia (Fig. 124) with sterigma narrow; antrum sclerotized; base of ductus wide; signum barely notched.

###### Holotype.

Female: 11-SRNP-30760, DNA barcoded, **Costa Rica:** Guanacaste: ACG: Sector Pitilla, Estación Pitilla 540 m, 19.iii.2011, Manuel Ríos. Reared from *Drymonia alloplectoides* (Gesneriacea) (Janzen and Hallwachs 2013). Deposited in INBio. **Paratypes: Costa Rica:** Guanacaste: ACG: Sector Pitilla, Sendero Caribe, 660 m, 1F 6.vi.2012, Calixto Moraga, Sendero Rótulo 510 m, 1M 23.xi.2008, C. Moraga; Alajuela: ACG: Sector Rincón Rainforest, Estación Leiva 454 m, 1F 17.ix.2009, F. Quesada & R. Franco, Jacobo 461 m, 1F 4.iv.2012, Edwin Apu, Manta Hugo 491 m, 1M 16.iii.2009, H. Cambronero & F. Quesada (INBio, EME, USNM).

###### Distribution and biology.

This species has been collected in Costa Rica (Fig. 164) on the east side of Cordillera Volcánica de Guanacaste from 400 to 660 m in ACG rain forest.

###### Food plant records.

*Ethmia normgershenzi* has been reared from larvae feeding on Gesneriaceae: *Drymonia alloplectoides* Hanst, *Drymonia macrophylla*, *Drymonia serrulata*.

###### Immature stages

(Fig. 145). Dorsum: Head black, thoracic shield bright orange, T2 black, T3-A1 bright orange, A2-A5 black, A6-A7 orange A8-A9 black, A10 orange with few small dots at dorsum. Lateral: As in dorsum. The fully grown larvae differ strikingly from those of other *Ethmia* in the Catapeltica complex in color pattern, being black with bright orange bands in *Ethmia normgershenzi*.

###### Etymology.

*Ethmia normgershenzi* is named in honor of Norm Gershenz of San Francisco, California, one of the two motors and electricity that invented the Conservation Parking Meter, the traveling insect zoo, and www.SaveNature.org, and has hustled for two decades on behalf of ACG rain forest land purchase for permanent wildland conservation.

##### Ethmia nicholsonorum

Taxon classificationAnimaliaLepidopteraDepressariidae

Phillips sp. n.

http://zoobank.org/225EA69E-E350-4DC4-943B-43A041E157D5

Figures 35 , 80 , 125 , 164

###### Diagnosis.

*Ethmia nicholsonorum* can be distinguished from *Ethmia normgershenzi*, its most similar species, by a blade-like ovipositor formed by papillae anales flattened laterally in female genitalia.

###### Description.

Male: FW length 10.6–10.8 mm (n = 3). **Head:** Labial palpus elongated, reaching collar anteriorly, white with dark rings basal, medial in II segment, basal and apical in III segment. Proboscis, front and crown white, occipital tuft black at mid-dorsum. **Thorax:** Ground color whitish, four large black spots near apices of tegula and at sides of scutellum. FW ground color whitish, markings blackish, two distinct spots at base; two oblique irregular dark blotches at middle and before directed to but unattached to an elongated mark line from middle to termen. HW ground color whitish darker at margins, a narrow costal fold with blackish long fringe at ventral, with a thin white scale-pencil. **Abdomen:** Dorsal pale brownish, genital scaling pale ochreous. Genitalia (Fig. 80) with uncus membranous; basal processes narrow, broadened basally; distal margin of valva with four short spines enlarged at base; sacculus narrow, margin emarginated at 0.7× from base.

Female: FW length 10.9–11 mm (n = 2). **Head and thorax:** As described for male except HW unmodified. **Abdomen:** Genitalia (Fig. 125) with papillae anales elongated and pointed; sterigma narrow; antrum sclerotized; base of ductus wide with a long sclerotized band; signum barely notched.

###### Holotype.

Male: 09-SRNP-1051, DNA barcoded, **Costa Rica:** Aajuela: ACG: Sector San Cristobal, Puente Palma 460 m, 15.iv.2009, Elda Araya. Reared from *Cordia panamensis* (Boraginaceae) (Janzen and Hallwachs 2013). Deposited in INBio. **Paratypes: Costa Rica:** Alajuela: ACG: Sector San Cristobal, Puente Palma 460 m, 2M 2F 15–19.iv.2009, Elda Araya (INBio, EME, USNM).

###### Distribution and biology.

*Ethmia nicholsonorum* has just been obtained by rearing from rain forest in Sector San Cristobal of the ACG in northern Costa Rica at 460 m (Fig. 164).

###### Food plant records.

*Ethmia nicholsonorum* have been reared from larvae feeding on Boraginaceae: *Cordia panamensis*. Immature stages were not described or photographed.

###### Etymology.

*Ethmia nicholsonorum* is named in honor of Ford and Catherine Nicholson of Dellwood, Minnesota, for their timely support of ACG rain forest land purchase, of Costa Rica as a country, and of Carroll Henderson’s conservation activities on behalf of birds, the Minnesota Department of Natural Resources, and ACG.

##### Ethmia lichyi

Taxon classificationAnimaliaLepidopteraDepressariidae

Powell

Figures 36 , 81 , 126 , 146 , 165

Ethmia lichyi Powell, 1973: 160.

###### Diagnosis.

This species is most similar to *Ethmia hendersonorum*, and it can be discriminated externally by its smaller size, the FW white ground color and the antrum broadly sclerotized in the female genitalia.

###### Description.

Male: FW length 16.4–17.4 mm (n = 3). **Head:** Labial palpus elongate, reaching crown with brownish marks, proboscis, front and crown white, occipital tufts black at mid-dorsum. **Thorax:** Pale gray, pronotum with five blue-black spots on posterior half. FW ground color white, with a series of elongated dark marking and spots over costal half, the most conspicuous a wide line from middle of cell to termen below apex; base paler with two dark small spots on posterior half. HW ground color white becoming brownish at apex; costa with a double hair brush, white at base becoming blackish distally, posterior part a long white hair pencil enclosed in a pouchlike fold, between costal and subcostal veins. **Abdomen:** Dorsal scaling brown, ventral white; first segment laterally with elongate ochreous-white patch concealing an area of specialized scaling which forms a pouchlike fold; genital scaling ochreous. Genitalia (Fig. 81) with uncus and gnathos absent; basal processes wide; valva emarginated at 0.5× from base, a cluster of about 10 distal spines.

Female: FW length 16.8–19.1 mm (n = 3). **Head and thorax:** As described for male except by modification on HW. **Abdomen:** Pouchlike fold absent. Genitalia (Fig. 126) with a narrow sterigma; antrum widely sclerotized; signum deeply notched.

###### Holotype.

Male: Venezuela, Cuenca del Río Borborata (Sept. l948) [EME, examined].

###### Distribution and biology.

*Ethmia lichyi* is distributed from Guatemala to Venezuela and Brazil. In Costa Rica (Fig. 165) it has been collected on both slopes up to 1200 m. In ACG it has been collected in the rain forest.

###### Food plant records.

*Ethmia lichyi* has been reared from larvae feeding on Boraginaceae: *Cordia bicolor* A. DC., *Cordia collococca* L., *Cordia eriostigma* Pittier, *Cordia panamensis* L. Riley, *Cordia porcata* Nowicke.

###### Immature stages

(Fig. 146). Dorsum: Head black, thoracic shield bright orange, T2-A6 black, A7 with whitish band at dorsum, A9-10 whitish with small dark spots on it. Lateral: Black except by a small white spot on segment A1. Last instar larva distinct from that of all other *Ethmia* species known in Costa Rica.

###### Parasitoids.

Hymenoptera: Braconidae: Microgastrinae: *Apanteles* Rodriguez109 (n = 4).

##### Ethmia hendersonorum

Taxon classificationAnimaliaLepidopteraDepressariidae

Phillips sp. n.

http://zoobank.org/17DB826F-C167-4D8B-BFE8-498261A36841

Figures 37 , 82 , 127 , 165

###### Diagnosis.

This species is most similar to *Ethmia lichyi*, and it can be distinguished by its larger size and FW yellowish ground color and by the antrum being lightly sclerotized in the female genitalia.

###### Description.

Male: FW length 17.9–18.8 mm (n = 3). **Head:** Labial palpus elongate, reaching crown, yellowish, with brownish marks, third segment completely black; proboscis, front and crown yellowish; occipital tufts black at mid-dorsum. **Thorax:** Yellowish, pronotum with five blue-black spots on posterior half. FW ground color yellowish, with a series of elongated dark marking and spots over costal half, the most conspicuous a wide line from middle of cell to termen below apex, dorsal area paler with two dark spots. HW ground color whitish becoming brownish at apex; costa with a double hairbrush, white at base becoming blackish distally. **Abdomen:** Dorsal scaling brown, ventral yellowish; first segment laterally with elongate ochreous-white patch concealing an area of specialized scaling which forms a pouchlike fold; genital scaling ochreous. Genitalia (Fig. 82) with uncus and gnathos absent; basal processes wide; valva emarginated at 0.5× from base; a cluster of about 10 large spines distal.

Female: FW length 18.8–19.7 mm (n = 2). **Head and thorax:** As described for male, except modification on HW and abdomen. **Abdomen:** Genitalia (Fig. 127) with sterigma narrow; antrum with a narrow sclerotized band posteriorly; signum deeply notched.

###### Holotype.

Male: INB0004222359, DNA barcoded, **Costa Rica:** Limón: Área de Conservación La Amistad Caribe: Veragua Rain Forest, Campamento, 400 m, 19.vii.2009, R. Villalobos. Deposited in INBio. **Paratypes: Costa Rica:** Heredia: Cordillera Volcánica Central, Sarapiquí, 10 km SE LA Virgen, 2F 18/20.ii.2003, D. Brenes. Limón: Área de Conservación La Amistad Caribe: Veragua Rain Forest, Campamento, 400 m, 1M 1F 19.vii.2009, R. Villalobos (INBio, EME, USNM).

###### Distribution and biology.

*Ethmia hendersonorum* has been found in Costa Rica (Fig. 165) on the Caribbean slope at 400 m, but not yet in ACG. The food plants and immature stages are unknown.

###### Etymology.

*Ethmia hendersonorum* is named in honor of Carrol and Ethelle Henderson of Blaine, Minnesota, for their lifetime careers of non-game conservation in Minnesota and Costa Rica, and support for biodiversity-directed ecotourism of Costa Rica.

##### Ethmia transversella

Taxon classificationAnimaliaLepidopteraDepressariidae

Busck

Figures 38 , 83 , 128 , 165

Ethmia transversella Busck, 1914b: 53

###### Diagnosis.

*Ethmia transversella* can be distinguished by the valva of the male genitalia produced into a short “plume”.

###### Description.

Male: FW length 14.0–15.6 mm (n = 3). **Head:** Labial palpus moderately elongate, grayish, broad black bands basal and subapical of II and III segment. **Thorax:** Gray, collar darker narrowly, a pair of small blackish spots on middle of pronotum, four larger spots lateral. FW ground color gray, with blackish markings, the most evident a curved narrow blotch from middle of costa reaching a longitudinal blotch that goes from center to before termen; an elongated inwardly directed blotch at terminal area from distal end of longitudinal blotch to torsum. HW ground color whitish, brownish along costal on terminal margin; costal fold enclosing a whitish brush of hair scales. **Abdomen:** Gray, genital scaling ochreous. Genitalia (Fig. 83) with basal processes broad basally; valva emarginated before apex with a group of distal curved spines; valva apex produced into a short plume.

Female: FW length: 16.8–19.1 mm (n = 2). **Head and thorax:** As described by male, HW unmodified. **Abdomen:** Genitalia (Fig. 128) with antrum sclerotized; signum a large and well-defined notched keel.

###### Holotype.

Male: Costa Rica, Juan Viñas [no date] (W. Schaus) [USNM, examined].

###### Distribution and biology.

*Ethmia transversella* has been collected in Costa Rica (Fig. 165) throughout the country from 1200 to 1750 m. It occurs in ACG rain forest. The food plants and immature stages are unknown.

##### Ethmia randyjonesi

Taxon classificationAnimaliaLepidopteraDepressariidae

Phillips sp. n.

http://zoobank.org/9D17CDC5-1472-423A-B54D-24CA8B67F68D

Figures 39 , 84 , 129 , 166

###### Diagnosis.

*Ethmia randyjonesi* can be distinguished by valva produced anteriorly into a short broad membranous “plume” in male genitalia.

###### Description.

Male: FW length 15.9–16.4 mm (n = 3). **Head:** Labial palpus elongate, reaching crown, white, with black rings middle and subapical segment II, basal and apical segment III; proboscis, front and crown white; occipital tufts black at mid-dorsum. **Thorax:** Whitish, pronotum with five six-black spots on posterior half, two in the middle. FW ground color whitish, with a series of elongated dark markings, the most conspicuous: an oblique line arising at costa before middle reaching a longitudinal line that goes from middle to near termen; a curved line arising from costa at base reaching middle of FW at cell origin. HW ground color whitish becoming brownish at apex; costa with a double hairbrush, white at base becoming blackish distally. **Abdomen:** Dorsal scaling brown, ventral yellowish; genital scaling ochreous. Genitalia (Fig. 84) with basal processes wide, valva emarginated at 0.5× from base, a cluster of about 5 distal spines; valva produced into a short broad membranous “plume”.

Female: FW length 16.4–16.7 mm (n = 2). **Head and thorax:** As described for male except modification on HW. **Abdomen:** Genitalia as in (Fig. 129) with sterigma narrow; antrum with narrow sclerotized band posteriorly; signum deeply notched.

###### Holotype.

Male: 09-SRNP-110144, DNA Barcoded, **Costa Rica:** Guanacaste: Sector del Oro, Sendero Manta, 610 m, 15.xi.2009, F. Quesada & H Cambronero. Deposited in INBio. **Paratypes: Costa Rica:** Guanacaste: ACG, Sector Mundo Nuevo, Pozo #3, 634 m, 1F 18.x.2009, R. Franco & S. Ríos; Sector Cacao, Estación Gongora 557 m, 1F 29.v.2008, F. Quesada & R. Franco, 1F 10.viii.2007, S. Ríos & H. Cambronero, Laboratorio 1150 m, 1M 8.viii.2010, H. Cambronero & R. Franco, Roca Verde, 835 m 1F 12.viii.2007, F. Quesada & R. Franco; Sector del Oro, Serrano 585 m, 1M 1.viii.2008, R. Franco & H. Cambronero; Sector Maritza, Maritza 548 m, 1M 30.ix.2011, S. Ríos & H. Cambronero; Sector Pailas, Palmeras, 1368 m, 1F 10.vi.2010, S. Ríos & R. Franco, Manta Mona 1055 m, 1M 14.vi.2010, R. Franco & F. Quesada, Manta Rio Blanco 790 m, 1M 1F 9.x.2010, S. Ríos & R. Franco (BMNH, INBio, EME, USNM).

###### Distribution and biology.

*Ethmia randyjonesi* has been collected in Costa Rica (Fig. 166) from 500 to 1200 m in the ACG rain forest of Cordillera Volcánica de Guanacaste, Tilarán and Cordillera Volcánica Central. The food plants and immature stages are unknown.

###### Etymology.

*Ethmia randyjonesi* is named in honor of Randy Jones of Poland, Ohio for his support of the early germination and growth of INBio (Instituto Nacional de Biodiversidad) and of many years of the parataxonomists conducting the ACG biodiversity inventory.

##### Ethmia randycurtisi

Taxon classificationAnimaliaLepidopteraDepressariidae

Phillips sp. n.

http://zoobank.org/480086F8-6A2F-44D5-B3F5-2950C8BFCA68

Figures 40 , 85 , 130 , 166

###### Diagnosis.

This species can be distinguished by the long ochreous spines below apex of valva in male genitalia.

###### Description.

Male: FW length 13.3–13.4 mm (n = 2). **Head:** Labial palpus elongate, reaching crown, white, with black areas basal of segment II and segment III; proboscis front and crown whitish occipital tufts black at mid-dorsum, yellow lateral tufts. **Thorax:** Whitish, pronotum with 4 black spots on posterior half; collar and base of tegula black. FW ground color whitish with a series of elongated dark markings, the most conspicuous: a sinuate middle line from base almost to termen, expanding into a costal dark blotch reaching costa anteriorly and above tornus posteriourly; two dots at posterior half: one before middle and one bellow costal blotch. HW ground color whitish, pale brown towards margin; costal pinch fold present. **Abdomen:** Pale brownish, genital scaling pale ochreous. Genitalia (Fig. 85) with basal process broad; valva with apex produced into a short plume, a group of three to four long ochreous spines just below apex.

Female: FW length 13.3–15.5 mm (n = 2). **Head and thorax:** As described for male, HW unmodified. **Abdomen:** Genitalia (Fig. 130) with sterigma narrow; antrum with a narrow sclerotized band posteriorly; signum deeply notched.

###### Holotype.

Male: INBCRI0003101969, **Costa Rica:** Cartago, Paraíso, P.N. Tapantí, Send. La Pava 1400 m, 1.iv.1999, Roberto Delgado. Deposited in INBio. **Paratypes: Costa Rica:** Cartago: Paraíso, P.N. Tapantí Macizo de la Muerte, Administración 1200 m, 1M 1.xi.1999, R. Delgado; Sector Represa, Puente del Rio Porras, 300 m SE, 1660 m, 1F 1.ix.1999, 1F 1.viii. 2001, R. Delgado. Puntarenas: Monteverde, Est. La Casona 1520 m, 1M 1F 1.i.1994, N. Obando, 1F 3-17.ix. 1994, K. L. Martínez (INBio, EME, USNM).

###### Distribution and biology.

*Ethmia randycurtisi* has been collected in Costa Rica (Fig. 166) from 1200 to 1600 m in Cordillera Volcánica de Tilarán and Cordillera Volcánica Central. The food plants and immature stages are unknown.

###### Etymology.

*Ethmia randycurtisi* is named in honor of Randy Curtis of Arlington, Virginia and The Nature Conservancy, for his continuous encouragement, financial support, and advice on behalf of INBio’s germination in Santo Domingo de Heredia, the endowment establishment for ACG, and the ACG conservation activities of Daniel Janzen and Winnie Hallwachs.

#### Trifurcella species-group

The trifurcella species group (sensu Powell 1973) is characterized by a FW costal-posterior pattern (dark costal area and posterior pale area), an ochreous genital scaling and the valva with a cucullus “plume” and a distal bunch of strong seta. In Costa Rica three species are included in this group, two described as new here.

##### Ethmia miriamschulmanae

Taxon classificationAnimaliaLepidopteraDepressariidae

Phillips sp. n.

http://zoobank.org/E13E3441-B1F8-490E-ADE7-91B03BFE6CB2

**Figures 41–45.**
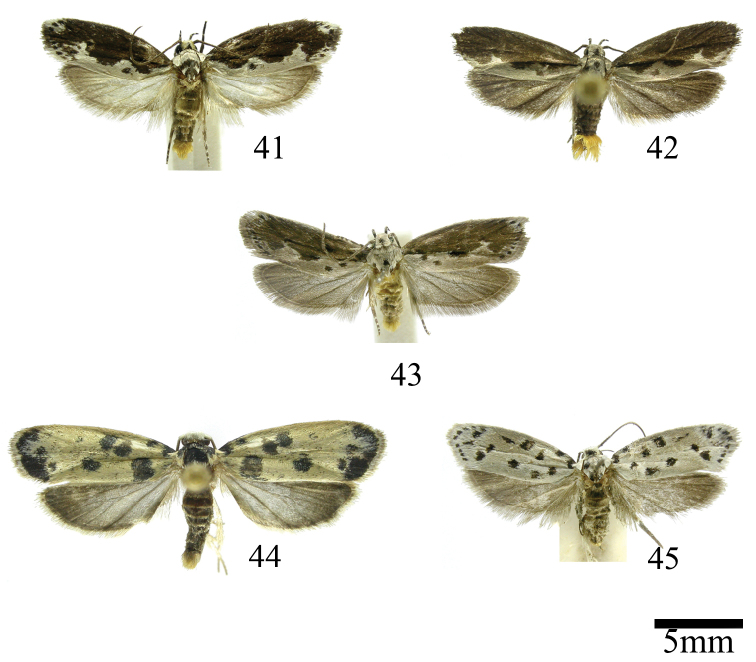
*Ethmia* from Costa Rica, adults: **41** *Ethmia miriamschulmanae*, paratype, male, INB0004336358 **42** *Ethmia similatella*, male, INBIOCRI001640251 **43** *Ethmia tilneyorum*, holotype, male, INB0004336361 **44** *Ethmia hammella*, male, 11-SRNP-101563 **45** *Ethmia linda*, female, INBIOCRI000680730.

Figures 41 , 86 , 131 , 167

###### Diagnosis.

This species can be discriminated easily from *Ethmia similatella* and *Ethmia tilneyorum* externally by the broad black band on the pronotum.

###### Description.

Male: FW length 10.3–10.5 mm (n = 3). **Head:** Labial palpus surpassing base of antenna, II segment brown basally, III segment brown basally and apically; proboscis and front brown before base of antenna, crown yellowish with middorsal occipital brown tuft. **Thorax:** White, base of tegula brown narrowly; pronotum with a middle wide brown band between tegula apices, scutellum with a brown spot. FW costal half dark brown from base to termen except apex which is white enclosing two dots on terminal line, longitudinal sinuated line separating dark costal half from paler posterior half, base with a brown spot at premedial line, torsum white. HW ground color whitish, getting dark towards apical margin, costal fold with double hair-brush present. **Abdomen:** Brown with ochreous genital scaling. Genitalia (Fig. 86) with uncus and gnathos absent; basal process broad, sinuated; valva with a pair of distal hooks, a cucullus “plume” present.

Female: FW length 10.5-10.7 mm (n = 3). **Head and thorax:** As described for male, HW unmodified. **Abdomen:** Genitalia (Fig. 131) with antrum sclerotized; signum with a small notched keel.

###### Holotype.

Male: 11-SRNP-103729, DNA Barcoded, **Costa Rica:** Guanacaste, Sector Santa Rosa, Luces 575 m, 1.vi.2011, H. Cambronero & R. Franco. Deposited in INBio. **Paratypes: Costa Rica:** Guanacaste: ACG: Sector Maritza, Casa Rafa 579 m, 1M 1.vi.2011, R. Franco & S. Ríos, 2F 4/5.vi.2011, H. Cambronero & R. Franco; Sector Mundo Nuevo, Manta Canon 700 m, 1M 1F 17.x.2009, F. Quesada & R. Franco; Sector Murciélago, Mirador Murciélago 87 m, 1M 1F 22/23.xi.2011, H. Cambronero & S. Ríos, Mirador Nance 133 m, 1M 1F 25/26.xi.2011, H. Cambronero & R. Franco; Sector Santa Elena, La Angostura 300 m, 2F 26.v.2009; Sector Santa Rosa, Luces 575 m, 1F 31.v.2011, H. Cambronero & R. Franco, Manta Naranjo 25 m, 2F 21/22.ix.2009, F. Quesada & R. Cambronero (BMNH, INBio, EME, USNM).

###### Distribution and biology.

*Ethmia miriamschulmanae* has been collected in Costa Rica (Fig. 167) in the dry forests of the Pacific side of Cordillera Volcánica de Guanacaste (ACG) and Península de Nicoya from 25 to 700 m.

###### Food plant records.

*Ethmia miriamschulmanae* has been reared from larvae feeding on Boraginaceae: *Varronia guanacastensis* (Standl.).

###### Etymology.

*Ethmia miriamschulmanae* is named in honor of Miriam Schulman of Los Angeles, California, for her early and continuous support of ACG germination and growth, for being a major friend of ACG and its philosophy, and for irrationally caring about caterpillars and spiders.

##### Ethmia similatella

Taxon classificationAnimaliaLepidopteraDepressariidae

Busck

Figures 42 , 87 , 132 , 167

Ethmia similatella Busck, 1920: 83; Powell 1973: 201.

###### Diagnosis.

*Ethmia similatella* is most similar to *Ethmia tilneyorum* and can be distinguished externally by FW with one dark spot on its base, and abdomen scaling darker.

###### Description.

Male: FW length 9.4–9.8 mm (n = 3). **Head:** Labial palpus elongate, exceeding base of antenna; proboscis white, front and crown whitish, an occipital black tuft at mid-dorsum. **Thorax:** Whitish with five medium black dots, a pair at tegula apices, a pair at scutellum and one at middle notum. FW ground color whitish, the longitudinal line dividing brown and whitish, sinuate. Costal half entirely brown, without apical white patch, posterior half with brown spot at 0.3× from base. HW ground color dark brown, costal fold present. **Abdomen:** Dark brown, ventral paler, genital scaling ochreous. Genitalia (Fig. 87) with basal processes elongate, valva with costal sclerotized “plume”, a bunch of apical setae.

Female: FW length 9.8–10.3 mm (n = 3). **Head and thorax:** As described by male, HW unmodified. **Abdomen:** Genitalia (Fig. 132) with sterigma narrow; antrum sclerotized; signum with a large deeply notched keel.

###### Holotype.

Male: Guatemala, Cayuga, [no date], W. Schaus [USNM, examined].

###### Distribution and biology.

*Ethmia similatella* has been recorded from western Mexico (Sinaloa) to Honduras, Guatemala, and Costa Rica (Powell 1973). In Costa Rica (Fig. 167) has been collected in the Pacific slope of Cordillera Volcánica de Guanacaste from 290 to 570 m, in ACG dry forest.

###### Food plant records.

*Ethmia similatella* has been reared from larvae feeding on Boraginaceae: *Varronia guanacastensis*.

##### Ethmia tilneyorum

Taxon classificationAnimaliaLepidopteraDepressariidae

Phillips sp. n.

http://zoobank.org/FE426B91-5713-437F-9F29-5372950EC48E

Figures 43 , 88 , 133 , 167

###### Diagnosis.

*Ethmia tilneyorum* is most similar to *Ethmia similatella* and can be distinguished externally by FW with two dark spots at base, and abdomen scaling paler.

###### Description.

Male: FW length 8.9–10.3 mm (n = 2). **Head:** Labial palpus whitish, brown medial in II segment, basal and subapical in III segment; proboscis brownish, front brown until base of antenna, crown whitish with an occipital brown tuft at mid-dorsum. **Thorax:** Collar and tegula whitish, pronotum with a middle small black spot. FW longitudinal line less sinuate at basal third; posterior half with two spots at base; apex whitish. HW brownish, costal fold present. **Abdomen:** Brownish, genital scaling pale ochreous. Genitalia (Fig. 88) with basal processes elongate; valva with a broad membranous “plume”, apex with a sclerotized projection with a group of small spines.

Female: FW length 8.7–10.4 mm (n = 2). **Head and thorax:** As described for male, HW unmodified. **Abdomen:** Genitalia (Fig. 133) with sterigma narrow; antrum sclerotized posteriorly; signum a sclerotized large patch, with anterior keel barely notched.

###### Holotype.

Male: INB0004336361, **Costa Rica:** Guanacaste, P. N. Santa Rosa, Pico Cerro Carbonal, 160 m, 15.vii.1992, R. Espinoza [hand-written label 92-SRNP-3488.3], reared from Boraginaceae: *Cordia gerascanthus*. Deposited in INBio. **Paratypes: Costa Rica:** Guanacaste, P. N. Santa Rosa, Pico Cerro Carbonal, 160 m, 1M 1F 15.vii.1992, R. Espinoza; Área de Conservación Tempisque, Sector Palo Verde 10m, 1M 4-10.iv.1995, Enia Navarro. (INBio, EME, USNM).

###### Distribution and biology.

*Ethmia tilneyorum* has been collected in Costa Rica (Fig. 167) on Pacific slope of Cordillera Volcánica de Guanacaste in ACG dry forest, and Península de Nicoya from 50 to 160 m.

###### Food plant records.

*Ethmia tilneyorum* has been reared from Boraginaceae: *Cordia gerascanthus*.

###### Immature stages

were not described or photographed.

**Parasitoids.** Diptera: Tachinidae: *Neaera* Wood01 (n = 2).

###### Etymology.

*Ethmia tilneyorum* is named in honor of Lou and Molly Tilney of the University of Pennsylvania at that time, for their extreme support of ACG land purchase and moral encouragement for ACG germination and early growth.

#### Hammella species-group

This group is characterized by a FW with a distinct pattern of blue spots and an unmodified HW in the male. The abdomen scaling is undifferentiated, the uncus is membranous and the gnathos absent; valva with cucullus “plume”. Papillae anales are membranous, posterior apophyses are short and anterior apophyses rudimentary; the antrum is weakly enlarged and the signum is a small sclerotized patch. In Costa Rica this group includes one species.

##### Ethmia hammella

Taxon classificationAnimaliaLepidopteraDepressariidae

Busck

Figures 44 , 89 , 134 , 168

Ethmia hammella Busck, 1910: 53; Powell 1973: 212.

###### Diagnosis.

*Ethmia hammella* is easily distinguished from other *Ethmia* in Costa Rica by the broad FW, which is pale yellowish with blue markings.

###### Description.

Male: FW length 9.0–10.5 mm (n = 3). **Head:** Labial palpus elongate, exceeding base of antenna; proboscis, front and crown beige, posterior margin of occipital tuft blue. **Thorax:** Dorsal scaling bluish with a beige posterior line on tegula. FW ground color pale yellow with distinct blue spots: three at costal area near medial line, one at postmedial line, one at base; terminal blotch blue. HW ground color pale brown, costa unmodified. **Abdomen:** Brownish, segments with caudal margin whitish, genital scaling whitish. Genitalia (Fig. 89) with basal processes broad, short; valva with a short preapical “plume” and group of 4 large curved setae distally.

Female: FW length 10.5–11.1 mm (n = 3). **Head and thorax:** As described for male. **Abdomen:** Genitalia (Fig. 134) with sterigma simple; ductus with basal sclerotized sleeve; antrum simple not enlarged; signum a small sclerotized patch.

###### Holotype.

Male: Costa Rica, Tuis, 5800’, [no date], W. Schaus [USNM, examined].

###### Distribution and biology.

*Ethmia hammella* has been collected in Costa Rica, Panamá and Colombia. In Costa Rica (Fig. 168), it has been found in the Caribbean lowlands and on both slopes of the Cordillera Volcánica de Guanacaste (ACG rain forest), Tilarán, Volcánica Central and Talamanca from 400 to 1600 m elevation.

#### Joviella species-group

This group is characterized by a FW pattern consisting of group of dots arranged in transverse lines. The male HW with a hair pencil enclosed in a Sc pinch-fold. The uncus and gnathos are absent and the valva has a cucullus “plume”. Papillae anales are membranous and posterior apophyses elongate; the antrum is enlarged and the signum simple. In Costa Rica this group includes one species.

##### Ethmia linda

Taxon classificationAnimaliaLepidopteraDepressariidae

Busck

Figures 45 , 90 , 135 , 168

Ethmia linda Busck, 1914b: 255; Powell 1973: 210.

###### Diagnosis.

*Ethmia linda* can be easily distinguished by its FW pattern, which is whitish with distinct brown spots.

###### Description.

Male: FW length 7.5–7.6 mm. (n = 3). **Head:** Labial palpus long, surpassing base of antenna, proboscis, front and crown white with a large, black middorsal spot on posterior half of head. **Thorax:** White, collar and tegula dark basally, four black spots on notum. FW ground color whitish, markings distinctive black round spots arranged in two oblique lines from costa to posterior margin; terminal area with two elongated spots at posterior half and a group of three irregular small dots before apex. HW brownish, costal fold broad. **Abdomen:** Whitish, genital scaling pale. Genitalia (Fig. 90) with basal processes narrow; valva produced into an apical “plume”, two large ochreous spines distally; sacculus emarginated.

Female: FW length 7.5–7.6 mm (n = 3). **Head and thorax:** As described for male, HW unmodified. **Abdomen:** Genitalia (Fig. 135) with sterigma simple, slightly sclerotized; base of ductus with a short, sclerotized sleeve; signum simple with one large and one smaller tooth.

###### Holotype.

Male: Venezuela, Caracas [no date] [USNM, examined].

###### Distribution and biology.

*Ethmia linda* has been recorded from Mexico to Venezuela. In Costa Rica (Fig. 168), has been collected on the Pacific slope of Cordillera Volcánica de Guanacaste at 600 m in ACG rain forest.

### Figures

**Figures 46–51.**
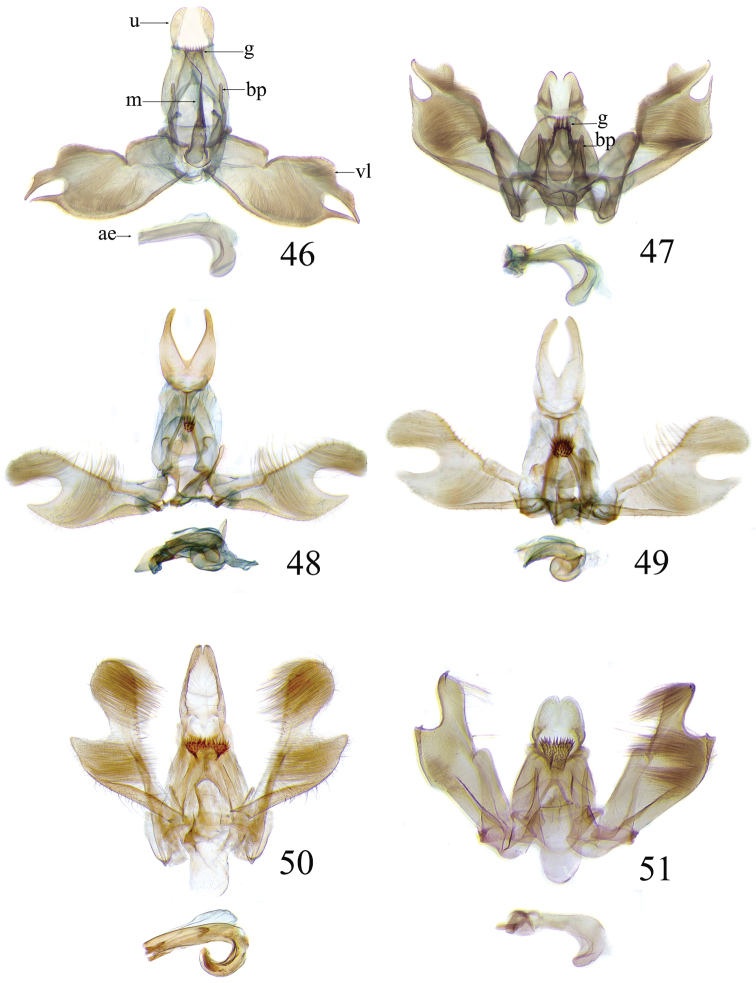
Male genitalia of *Ethmia*, aedeagus separated: **46** *Ethmia delliella*, INBIOCRI001341769, (u= uncus; g=gnathos; bp= basal process; m= manica; vl= valvae; ae= aedeagus) **47** *Ethmia bittenella*, INBIOCRI003118565 **48** *Ethmia festiva*, INBIOCRI001365810 **49** *Ethmia blaineorum*, paratype, INBIOCRI00227641 **50** *Ethmia scythropa* INBIOCRI001135224 **51** *Ethmia perpulchra*, INBIOCRI003146809.

**Figures 52–56.**
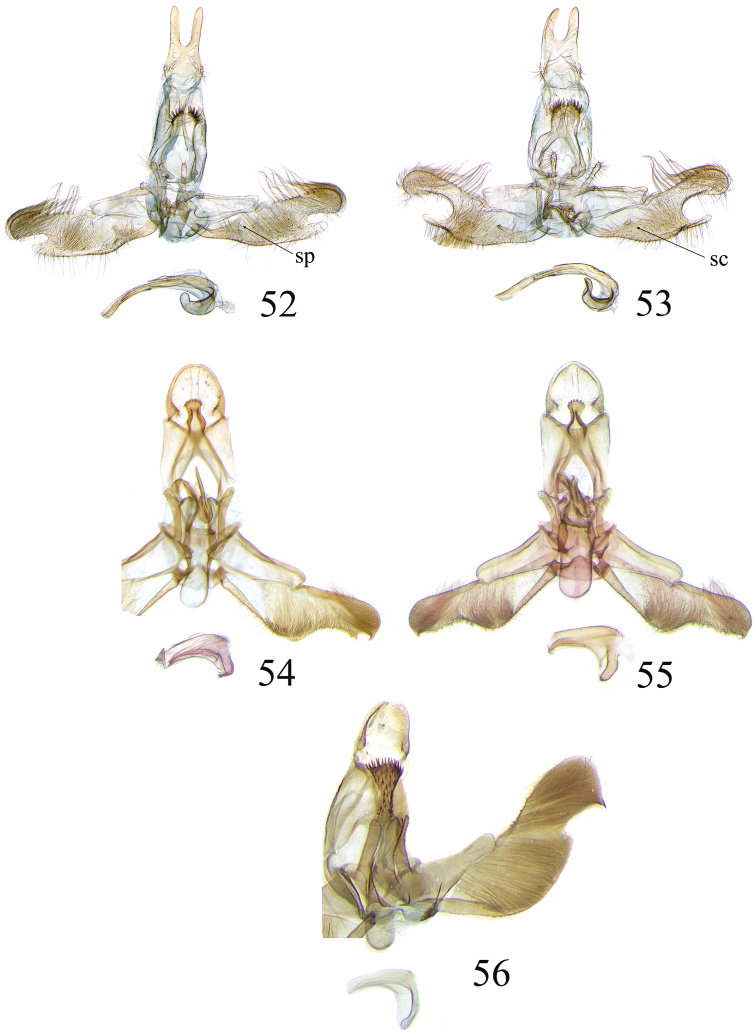
Male genitalia of *Ethmia*, aedeagus separated: **52** *Ethmia terpnota*, 10-SRNP-100341 (sp= saccular process) **53** *Ethmia millerorum*, paratype, 07-SRNP-35768, (s=sacculus) **54** *Ethmia elutella*, INBIOCRI001409679 **55** *Ethmia janzeni*, 10-SRNP-106466 **56** *Ethmia ungulatella*, INBIOCRI003521715.

**Figures 57–63.**
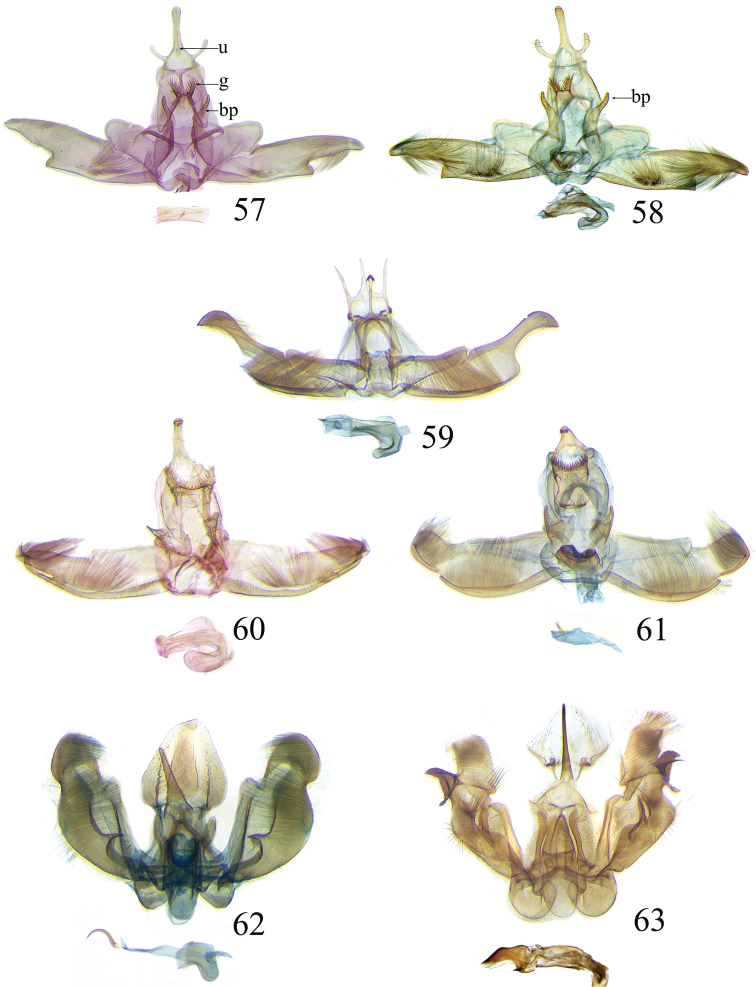
Male genitalia of *Ethmia*, aedeagus separated: **57** *Ethmia exornata*, 09-SRNP-106476 (u= uncus, g= gnathos, bp= basal process) **58** *Ethmia dianemillerae*, paratype, INB0003347736 (bp= basal process) **59** *Ethmia adrianforsythi*, paratype, INB0003230742 **60** *Ethmia phylacis*, 06-SRNP-104313 **61** *Ethmia mnesicosma*, 10-SRNP-115642 **62** *Ethmia chemsaki*, 09-SRNP-104698 **63** *Ethmia stephenrumseyi*, paratype, INBIOCRI002386616.

**Figures 64–69.**
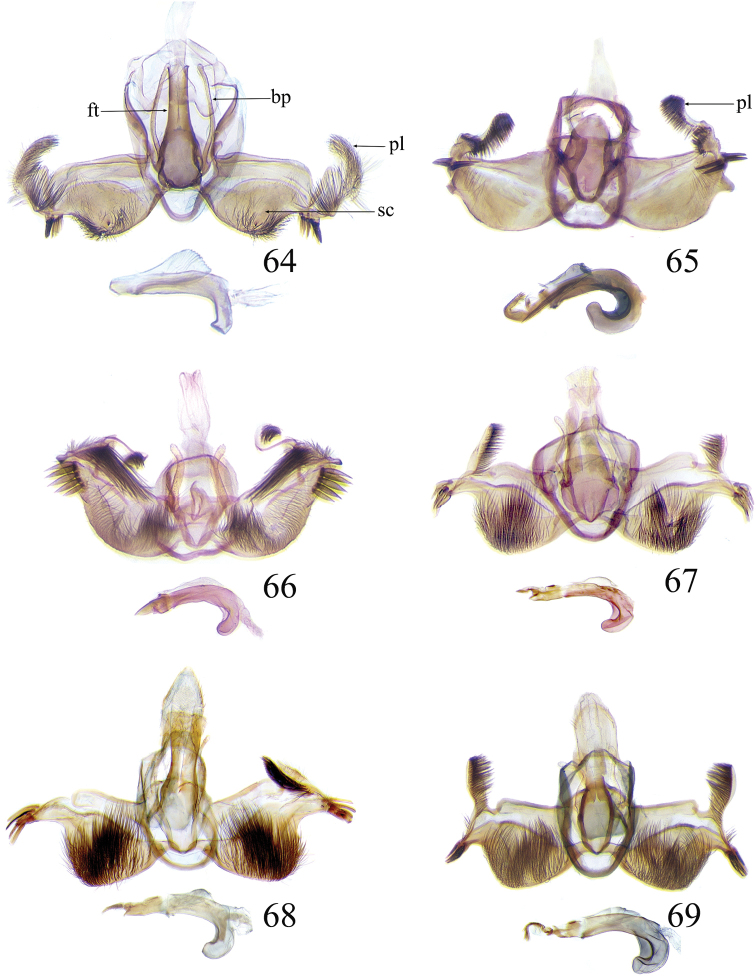
Male genitalia of *Ethmia*, aedeagus separated: **64** *Ethmia baliostola*, INBIOCRI001294907 (bp= basal process; ft= fultura; pl= plume; sc= sacculus) **65** *Ethmia berndkerni*, paratype, INB0001956655 (pl= plume) **66** *Ethmia dimauraorum*, paratype, INBIOCRI000356806 **67** *Ethmia duckworthi*, 09-SRNP-105091 **68** *Ethmia billalleni*, paratype, INBIOCRI001054805 **69** *Ethmia ehakernae*, paratype, JAP5546.

**Figures 70–74.**
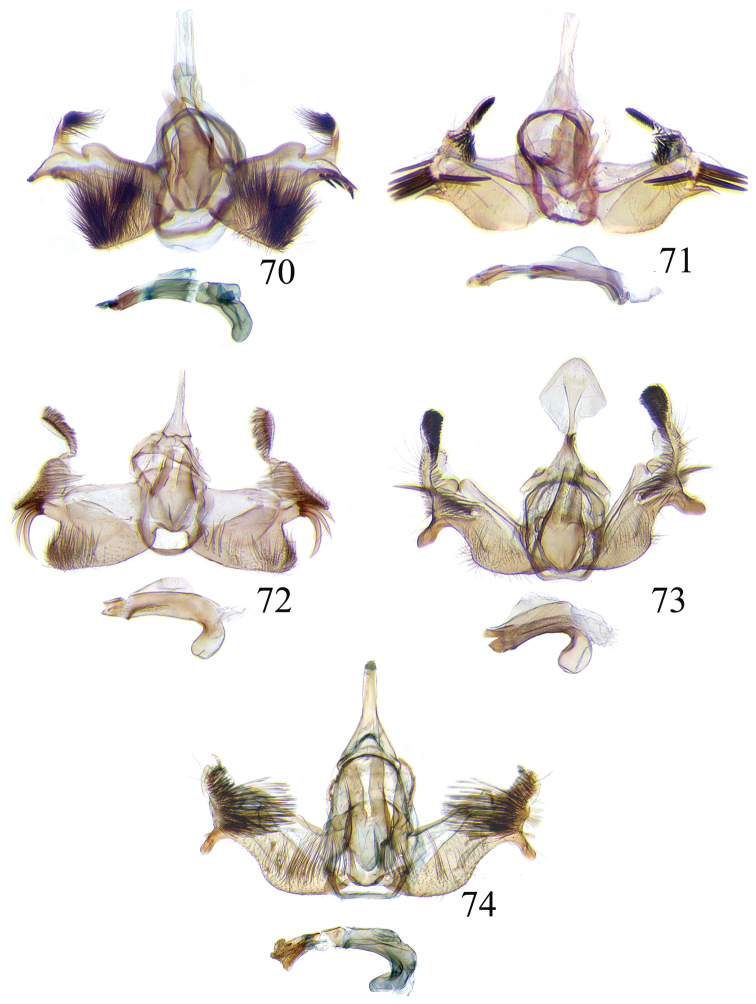
Male genitalia of *Ethmia*, aedeagus separated: **70** *Ethmia sandra*, EPR128 **71** *Ethmia helenmillerae*, INBIOCRI000227589 **72** *Ethmia johnpringlei*, paratype, INBIOCRI000180880 **73** *Ethmia nigritaenia*, INBIOCRI000080174 **74** *Ethmia laphamorum*, paratype, INB0004309613.

**Figures 75–80.**
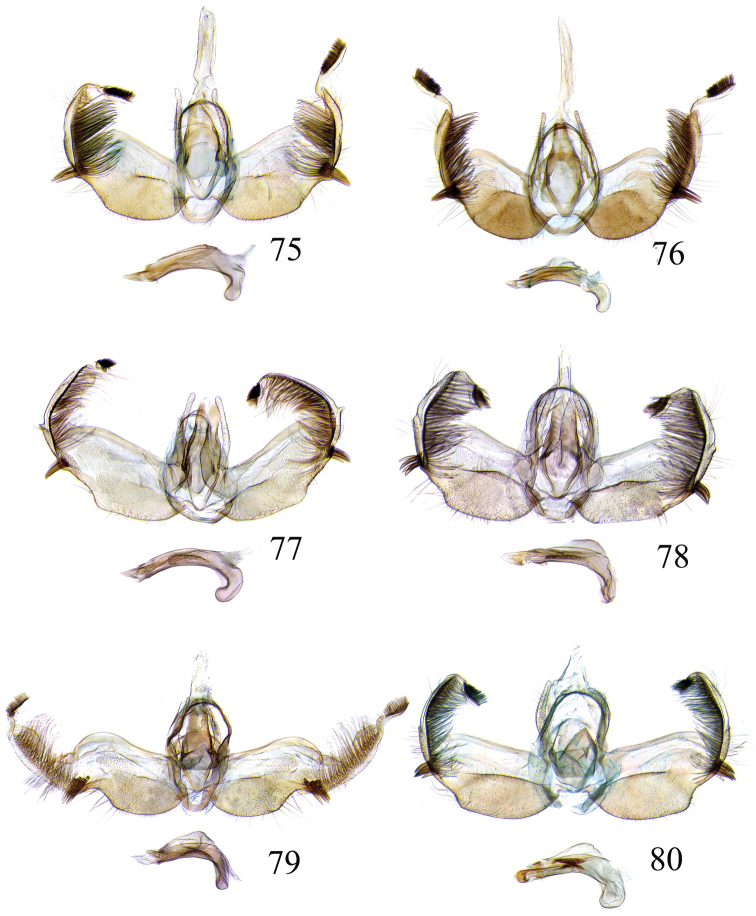
Male genitalia of *Ethmia*, aedeagus separated: **75** *Ethmia catapeltica*, 06-SRNP-41889 **76** *Ethmia petersterlingi*, paratype, 12-SRNP-102146 **77** *Ethmia lesliesaulae*, paratype, 05-SRNP-32421 **78** *Ethmia turnerorum*, paratype, 06-SRNP-23316 **79** *Ethmia normgershenzi*, paratype, 08-SRNP-32759 **80** *Ethmia nicholsonorum*, paratype, 09-SRNP-1050.

**Figures 81–85.**
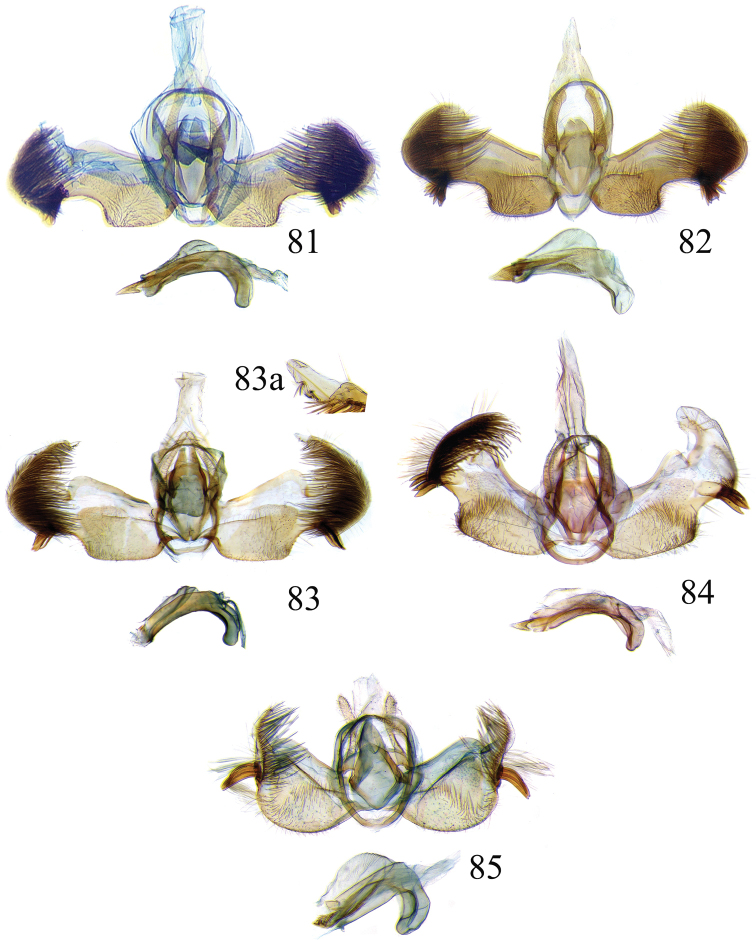
Male genitalia of *Ethmia*, aedeagus separated: **81** *Ethmia lichyi*, INBIOCRI000949149 **82** *Ethmia hendersonorum*, paratype, INB0004222481 **83** *Ethmia transversella*, INB0004310870: **83a** valva apex detail **84** *Ethmia randyjonesi*, 10-SRNP-114012 **85** *Ethmia randycurtisi*, paratype, INB0003141180.

**Figures 86–90.**
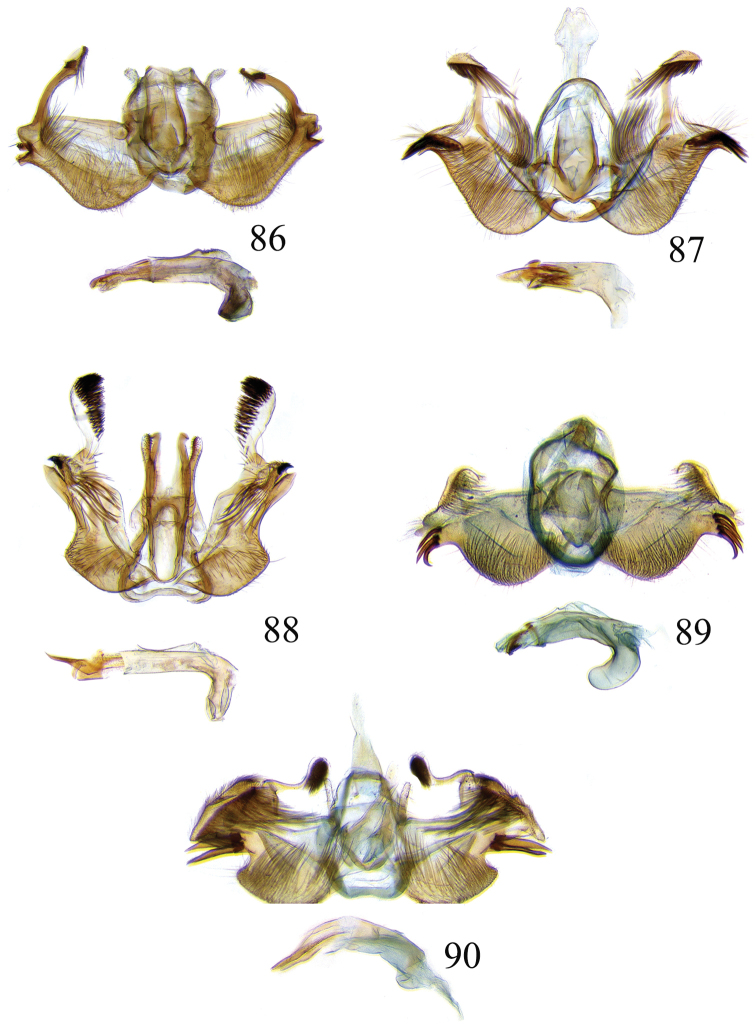
Male genitalia of *Ethmia*, aedeagus separated: **86** *Ethmia miriamschulmanae*, paratype, INBIOCRI000498355 **87** *Ethmia similatella*, INBIOCRI000392780 **88** *Ethmia tilneyorum*, paratype, INB0004336360 **89** *Ethmia hammella*, INBIOCRI000616532 **90** *Ethmia linda*, INBIOCRI000668043.

**Figures 91–96.**
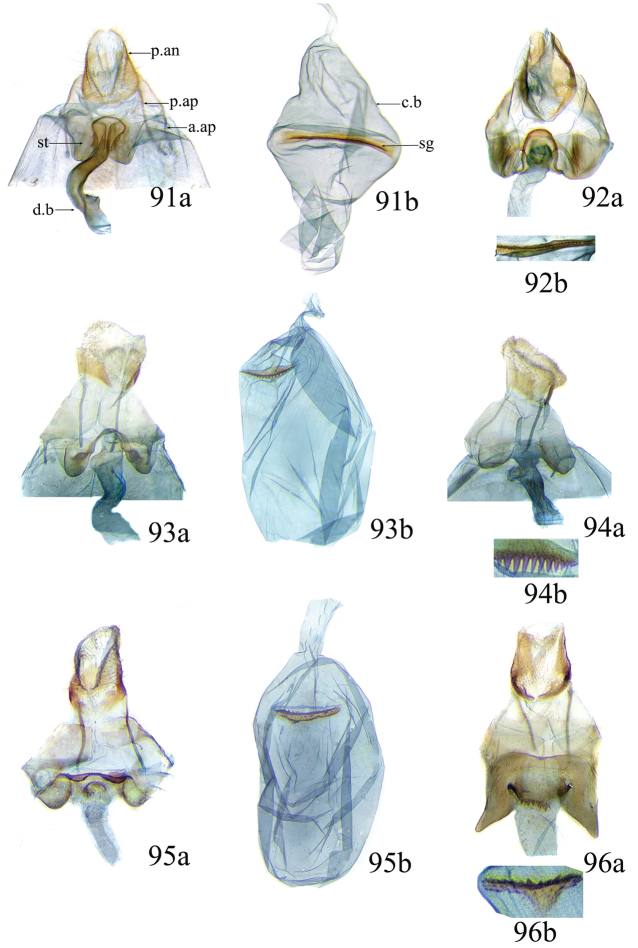
Female genitalia of *Ethmia*: **91** *Ethmia delliella*, INBIOCRI001341770: **91a** sterigma, segments VIII-X and base of ductus (p.an= papillae annales, p.ap= posterior apophyses, a.ap= anterior apohyses, st= sterigma, db= ductus bursae) **91b** corpus bursae (c.b= corpus bursae, sg= signum) **92** *Ethmia bittenella*, INBIOCRI002202414: **92a** sterigma, segments VIII-X and base of ductus **92b** signum detail **93** *Ethmia festiva*, INBIOCRI001258932: **93a** sterigma, segments VIII-X and base of ductus **93b** corpus bursae **94** *Ethmia blaineorum*, paratype, INBIOCRI00227633: **94a** sterigma, segments VIII-X and base of ductus **94b** signum detail **95** *Ethmia scythropa*, INBIOCRI000650740: **95a** sterigma, segments VIII-X and base of ductus **95b** corpus bursae **96** *Ethmia perpulchra*, INBIOCRI003146813: **96a** sterigma, segments VIII–X and base of ductus **96b** signum detail.

**Figures 97–101.**
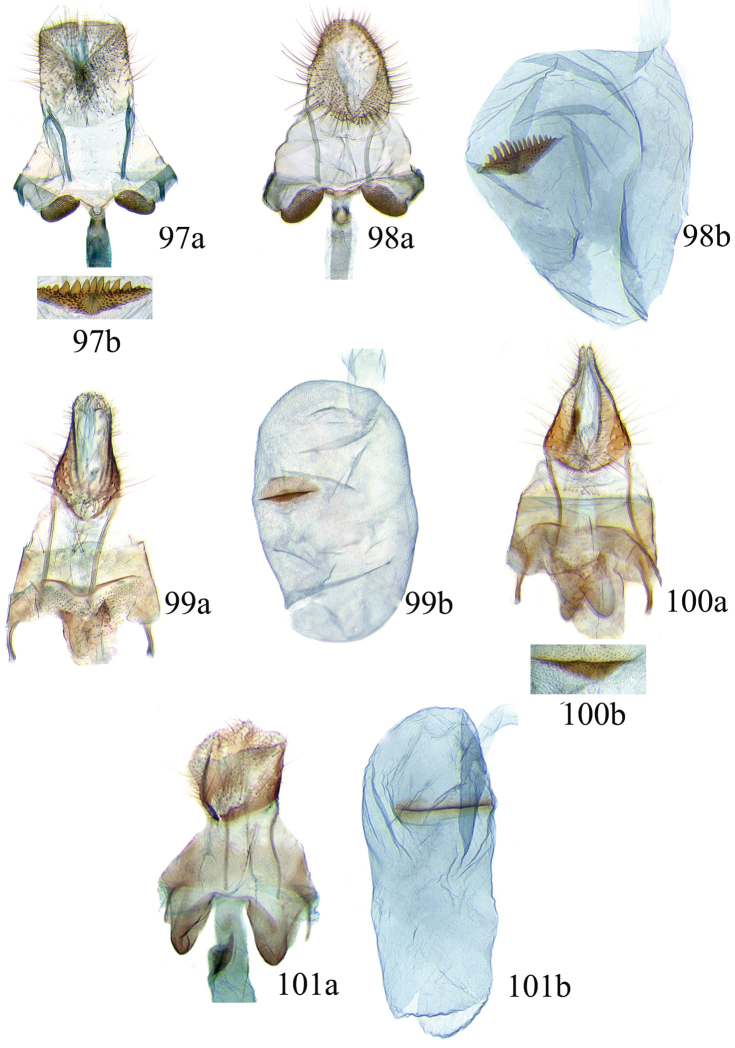
Female genitalia of *Ethmia*: **97** *Ethmia terpnota*, INBIOCRI001054759: **97a** sterigma, segments VIII-X and base of ductus **97b** signum detail **98** *Ethmia millerorum*, paratype, 07-SRNP-35770: **98a** sterigma **98b** corpus bursae **99** *Ethmia elutella*, INBIOCRI001409679: **99a** sterigma **99b** corpus bursae **99c** signum detail **100** *Ethmia janzeni*, 11-SRNP-100960: **100a** sterigma **100b** signum detail **101** *Ethmia ungulatella*, INBIOCRI003554528: **101a** sterigma **101b** corpus bursae.

**Figures 102–106.**
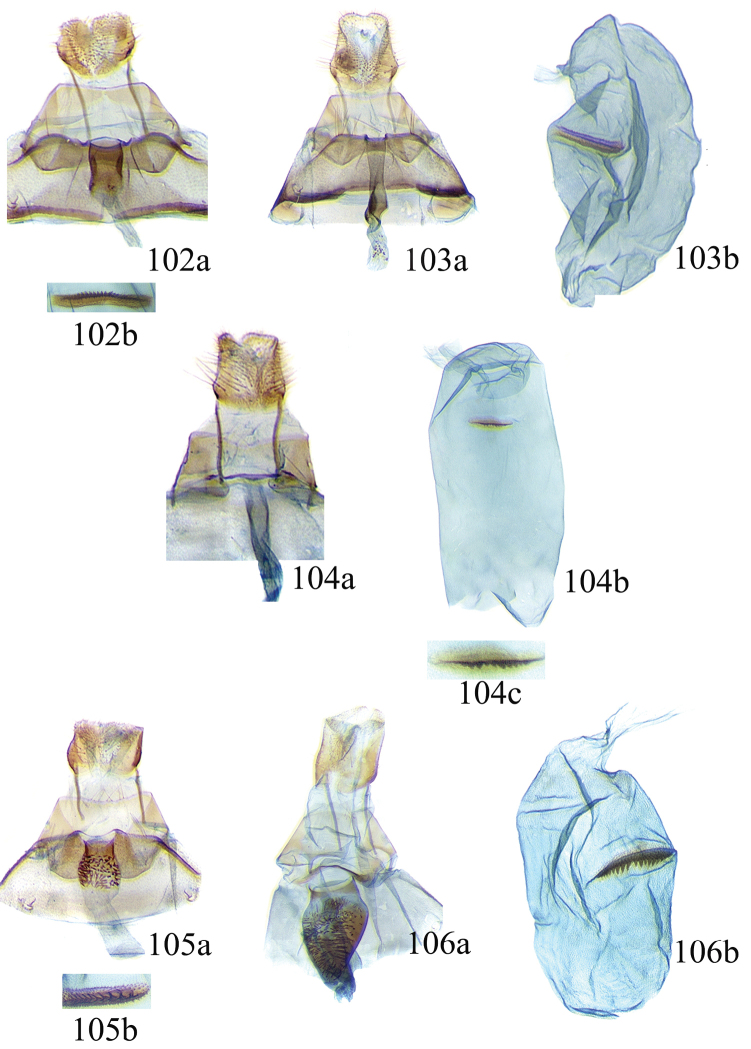
Female genitalia of *Ethmia*: **102** *Ethmia exornata*, 09-SRNP-105739: **102a** sterigma, segments VIII-X and base of ductus **102b** signum detail **103** *Ethmia dianemillerae*, paratype, 08-SRNP-5574: **103a** sterigma **103b** corpus bursae **104** *Ethmia adrianforsythi*, paratype,10-SRNP-106552: **104a** sterigma **104b** corpus bursae **104c** signum detail **105** *Ethmia phylacis*, 09-SRNP-104703: **105a** sterigma **105b** corpus bursae **105b** signum detail **106** *Ethmia mnesicosma*, 06-SRNP-16295: **106a** sterigma **106b** corpus bursae.

**Figures 107–109.**
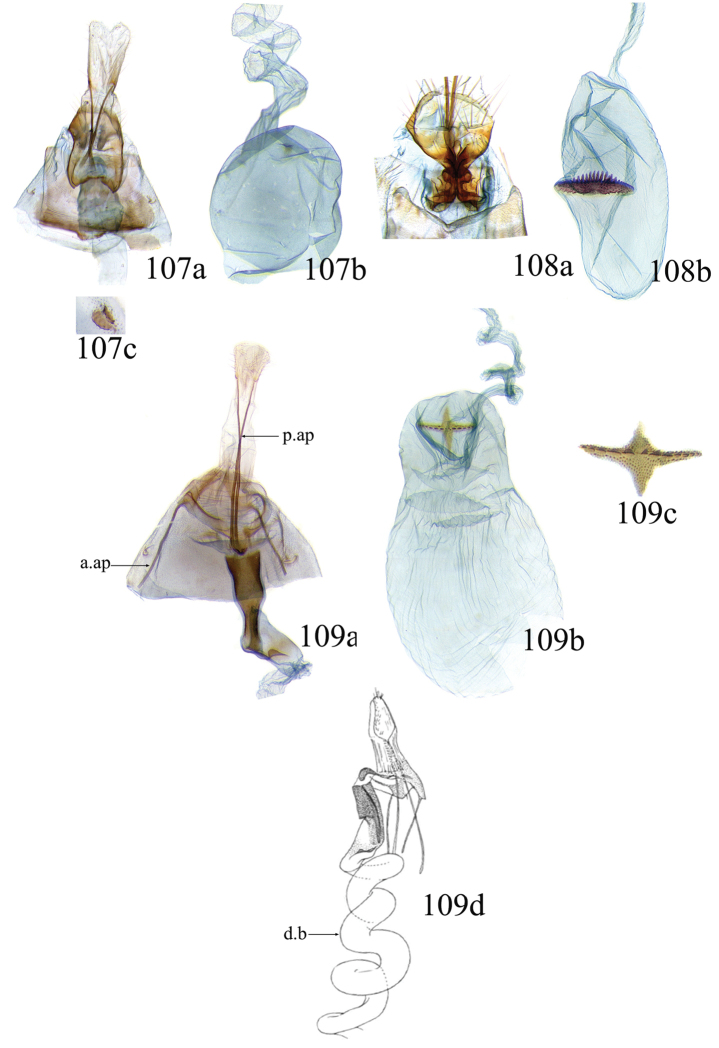
Female genitalia of *Ethmia*: **107** *Ethmia chemsaki*, 09-SRNP-104271: **107a** sterigma, segments VIII-X and base of ductus **107b** corpus bursae **107c** signum detail **108** *Ethmia stephenrumseyi*, paratype, INBIOCRI000487869: **108a** sterigma **108b** corpus bursae **109** *Ethmia baliostola*, 10-SRNP-41346: **109a** sterigma **109b** corpus bursae **109c** signum detail **109d** VIII segment lateral view and ductus bursae (d.b) as in Powell 1973.

**Figures 110–111.**
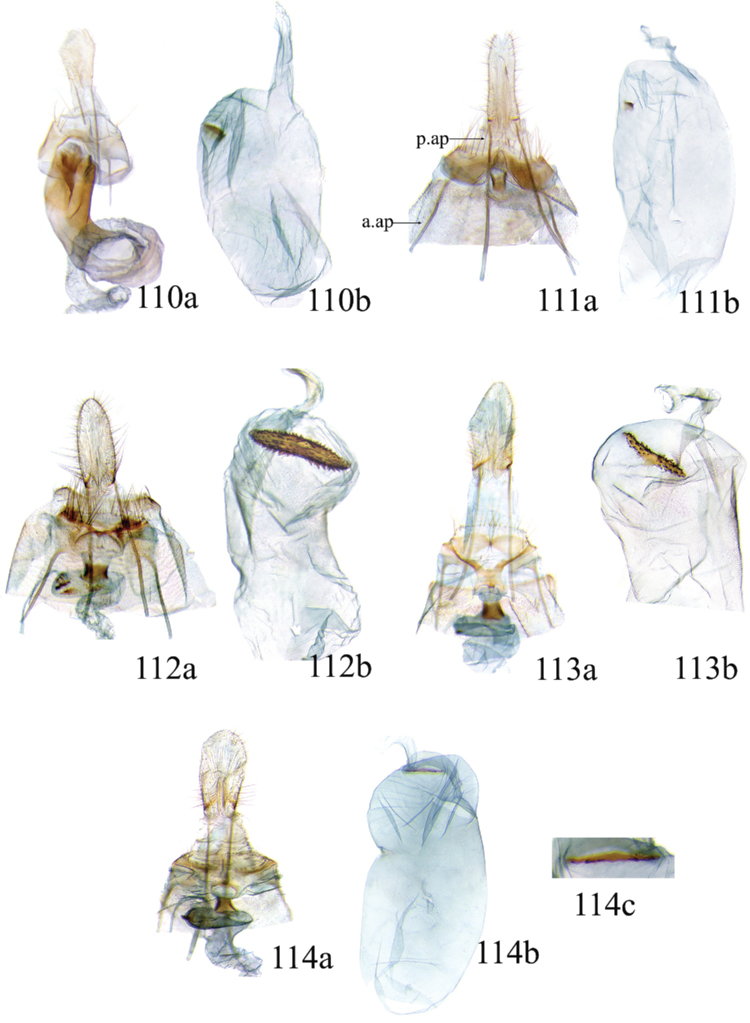
Female genitalia of *Ethmia*: **110** *Ethmia berndkerni*, paratype, INBIOCRI001143934: **110a** sterigma, segments VIII-X and base of ductus (p.ap= posterior apophyses, a.ap= anterior apophyses) **110b** corpus bursae **110c** signum detail **111** *Ethmia dimauraorum*, paratype, 09-SRNP-103903: **111a** sterigma **111b** corpus bursae **112** *Ethmia duckworthi*, 09-SRNP-111486: **112a** sterigma **112b** corpus bursae **113** *Ethmia billalleni*, paratype, INB0003702102: **113a** sterigma **113b** corpus bursae **114** *Ethmia ehakernae*, 10-SRNP-102922: **114a** sterigma **114b** corpus bursae **114c** signum detail.

**Figures 115–119.**
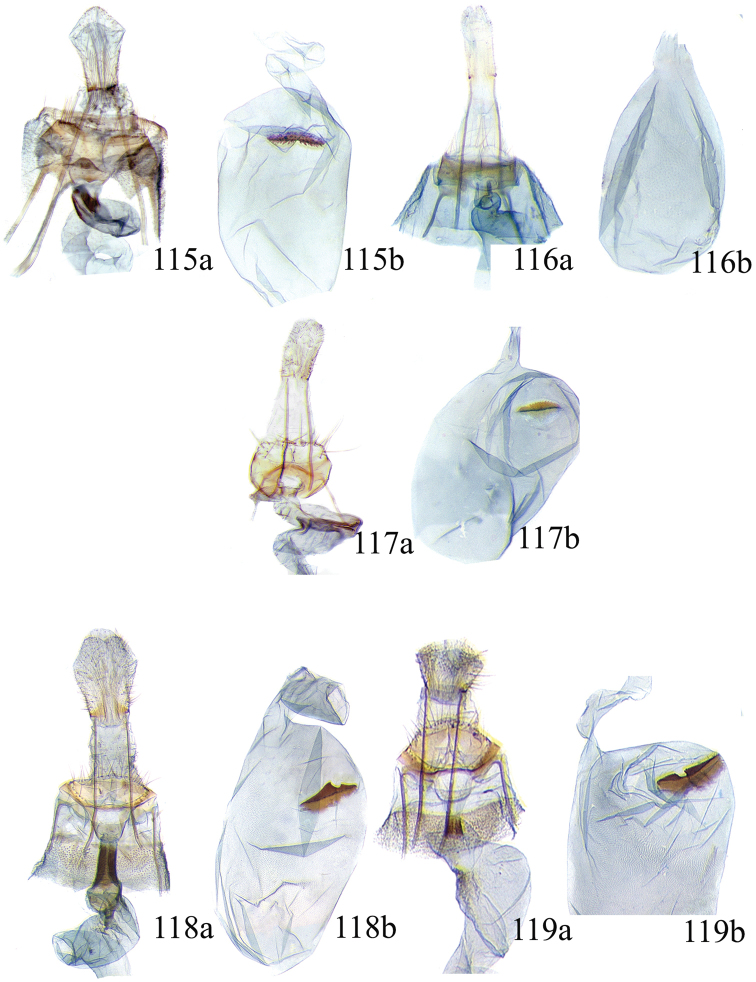
Female genitalia of *Ethmia*: **115** *Ethmia sandra*, EPR129: **115a** sterigma, segments VIII-X and base of ductus **115b** corpus bursae **116** *Ethmia helenmillerae*, paratype, INBIOCRI000306907: **116a** sterigma **116b** corpus bursae **117** *Ethmia johnpringlei*, paratype, INBIOCRI000180868: **117a** sterigma **117b** corpus bursae **118** *Ethmia nigritaenia*, INBIOCRI000152883: **118a** sterigma **118b** corpus bursae **119** *Ethmia laphamorum*, paratype, 06-SRNP-104471: **119a** sterigma **119b** corpus bursae.

**Figures 120–125.**
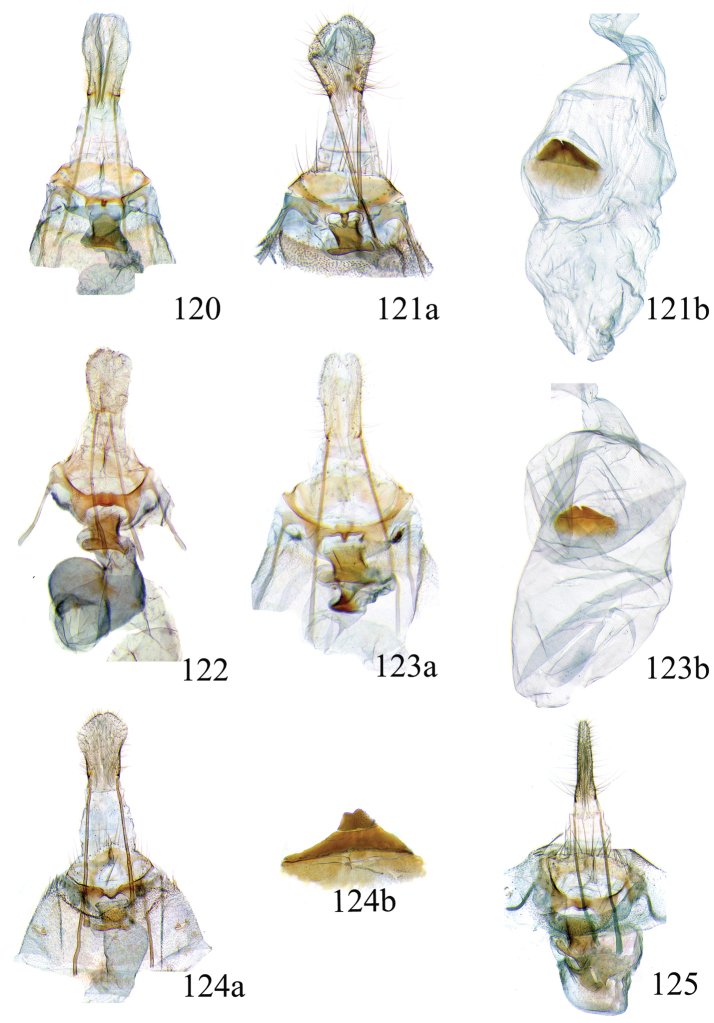
Female genitalia of *Ethmia*: **120** *Ethmia catapeltica*, INBIOCRI000343362: **120** sterigma, segments VIII-X and base of ductus **121** *Ethmia petersterlingi*, paratype, 10-SRNP-55705: **121a** sterigma **121b** corpus bursae **122** *Ethmia lesliesaulae*, paratype, 04-SRNP-56092: **122** sterigma, segments VIII-X and base of ductus **123** *Ethmia turnerorum*, paratype, JAP5578: **123a** sterigma **123b** corpus bursae **124** *Ethmia normgershenzi*, paratype, 09-SRNP-108130: **124a** sterigma **124b** signum detail **125** *Ethmia nicholsonorum*, paratype, 09-SRNP-1052: **125a** sterigma, segments VIII–X and base of ductus.

**Figures 126–130.**
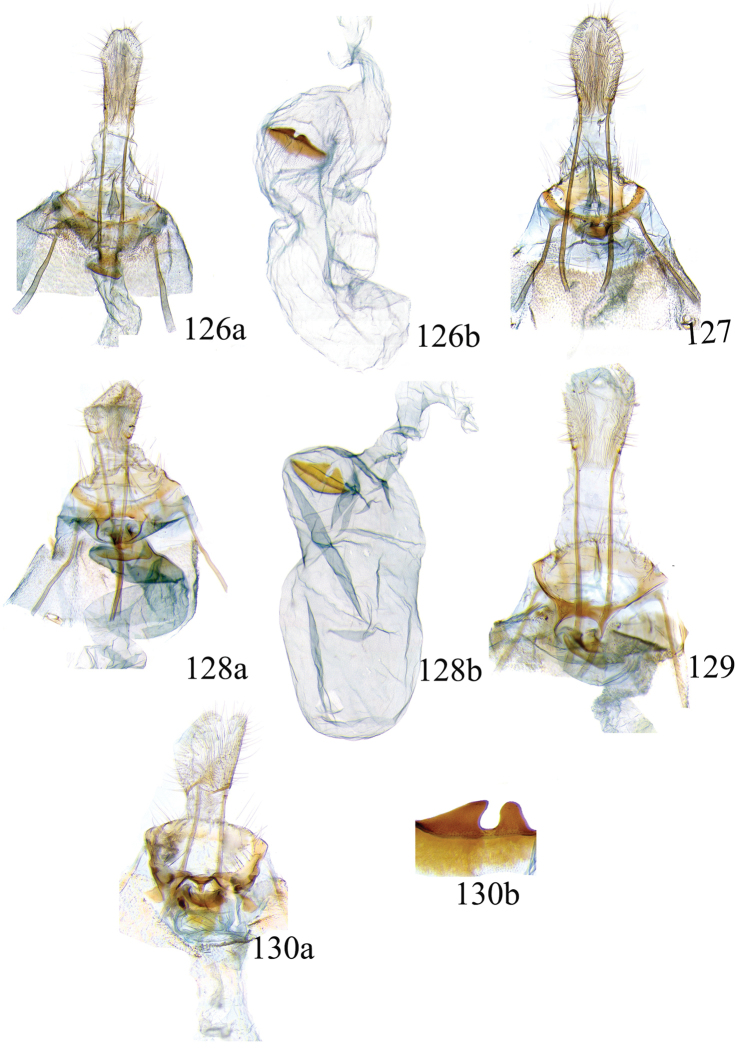
Female genitalia of *Ethmia*: **126** *Ethmia lichyi*, INBIOCRI000359212: **126a** sterigma, segments VIII-X and base of ductus **126b** corpus bursae **127** *Ethmia hendersonorum*, paratype, INB0003230788: **127** sterigma, segments VIII-X and base of ductus **128** *Ethmia transversella*, INB0004310870: **128a** sterigma **128b** corpus bursae **129** *Ethmia randyjonesi*, paratype, INBIOCRI000180836: **129** sterigma, segments VIII–X and base of ductus **130** *Ethmia randycurtisi*, paratype, INB0003141341: **130a** sterigma **130b** signum detail.

**Figures 131–135.**
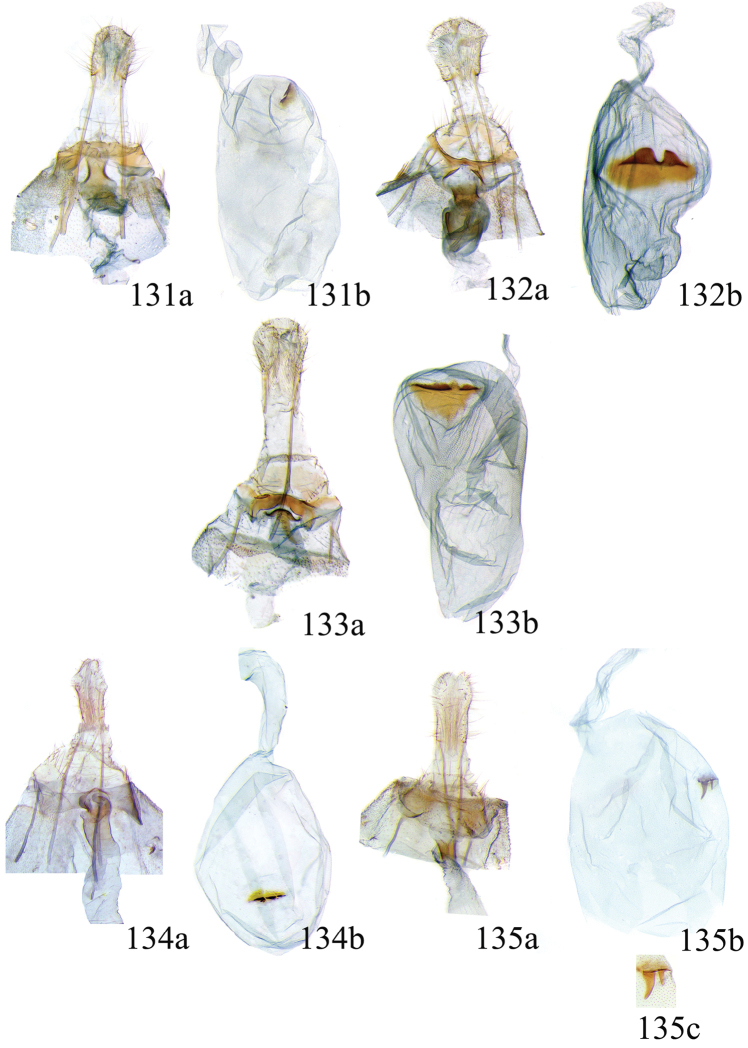
Female genitalia of *Ethmia*: **131** *Ethmia miriamschulmanae*, paratype, INBIOCRI000026254: **131a** sterigma, segments VIII-X and base of ductus **131b** corpus bursae **132** *Ethmia similatella*, 93-SRNP-3097: **132a** sterigma **132b** corpus bursae **133** *Ethmia tilneyorum*, paratype, INB0004336362: **133a** sterigma **133b** corpus bursae **134** *Ethmia hammella*, INBIOCRI000627721: **134a** sterigma **134b** corpus bursae **135** *Ethmia linda*, INBIOCRI000668063: **135a** sterigma **135b** corpus bursae **135c** signum detail.

**Figures 136–140.**
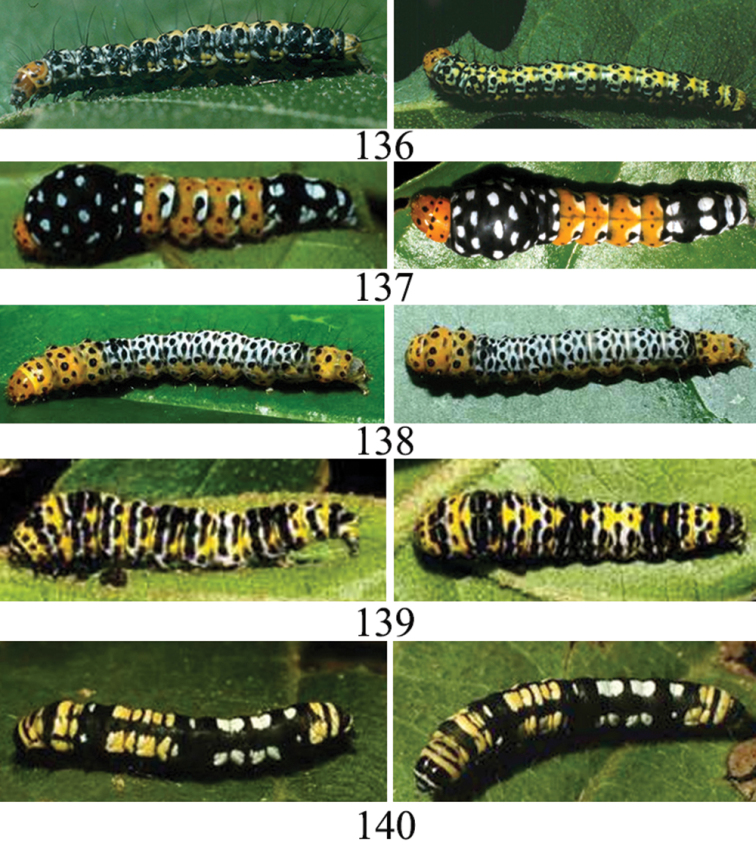
*Ethmia* last instar caterpillars, lateral and dorsal view: **136** *Ethmia delliella*, (87-SRNP-561) **137** *Ethmia scythropa*, (05-SRNP-22066) **138** *Ethmia millerorum*, (09-SRNP-36150) **139** *Ethmia dianemillerae*, (08-SRNP-5574) **140** *Ethmia mnesicosma* (98-SRNP-9666).

**Figures 141–146.**
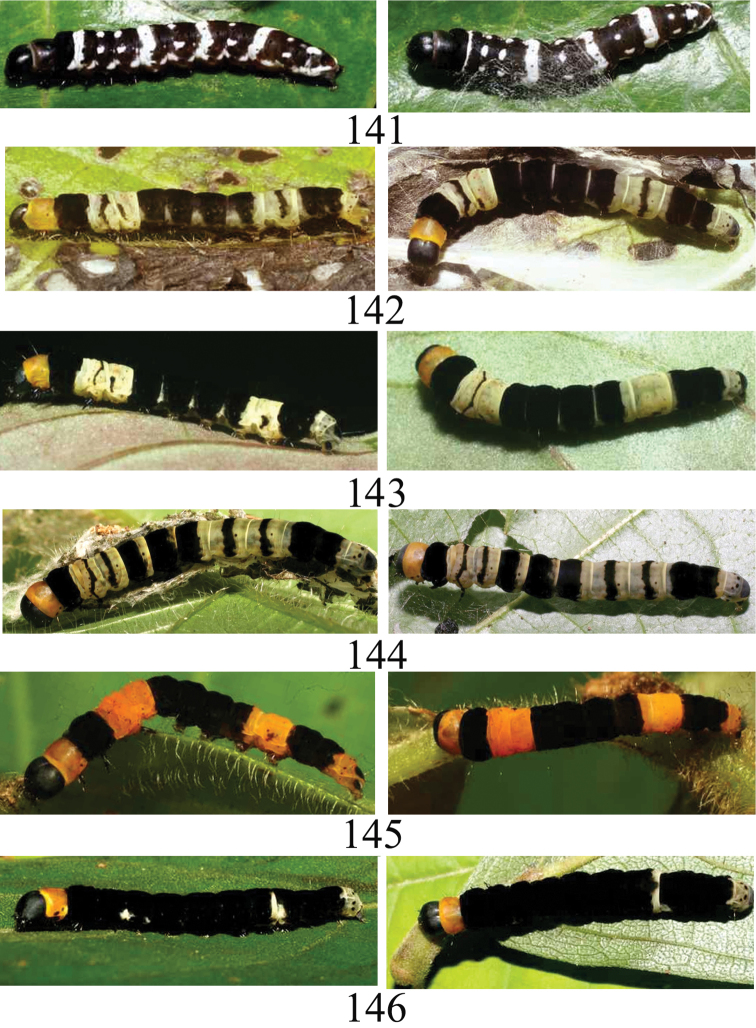
*Ethmia* last instar caterpillar, lateral and dorsal view: **141** *Ethmia baliostola* (10-SRNP-55253) **142** *Ethmia catapeltica* (09-SRNP-41280) **143** *Ethmia lesliesaulae* (09-SRNP-71951) **144** *Ethmia turnerorum* (06-SRNP-23316) **145** *Ethmia normgershenzi* (11-SRNP-30760) **146** *Ethmia lichyi* (08-SRNP-71629).

**Figures 147–152.**
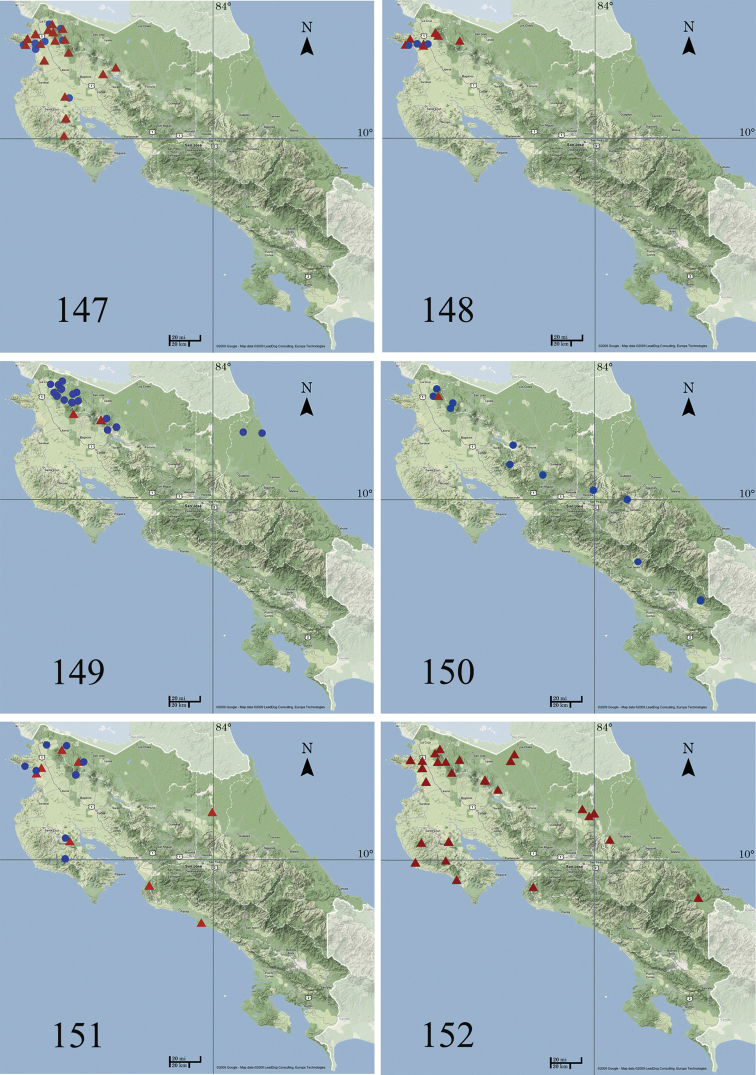
*Ethmia* collection localities in Costa Rica: **147** *Ethmia delliella*: red triangles; *Ethmia bittenella*: blue dots **148** *Ethmia festiva*: red triangles; *Ethmia blaineorum*: blue dots **149** *Ethmia perpulchra*: red triangles; *Ethmia scythropa*: blue dots **150** *Ethmia millerorum*: red triangles; *Ethmia terpnota*: blue dots **151** *Ethmia elutella*: red triangles; *Ethmia janzeni*: blue dots **152** *Ethmia ungulatella*: red triangles.

**Figures 153–158.**
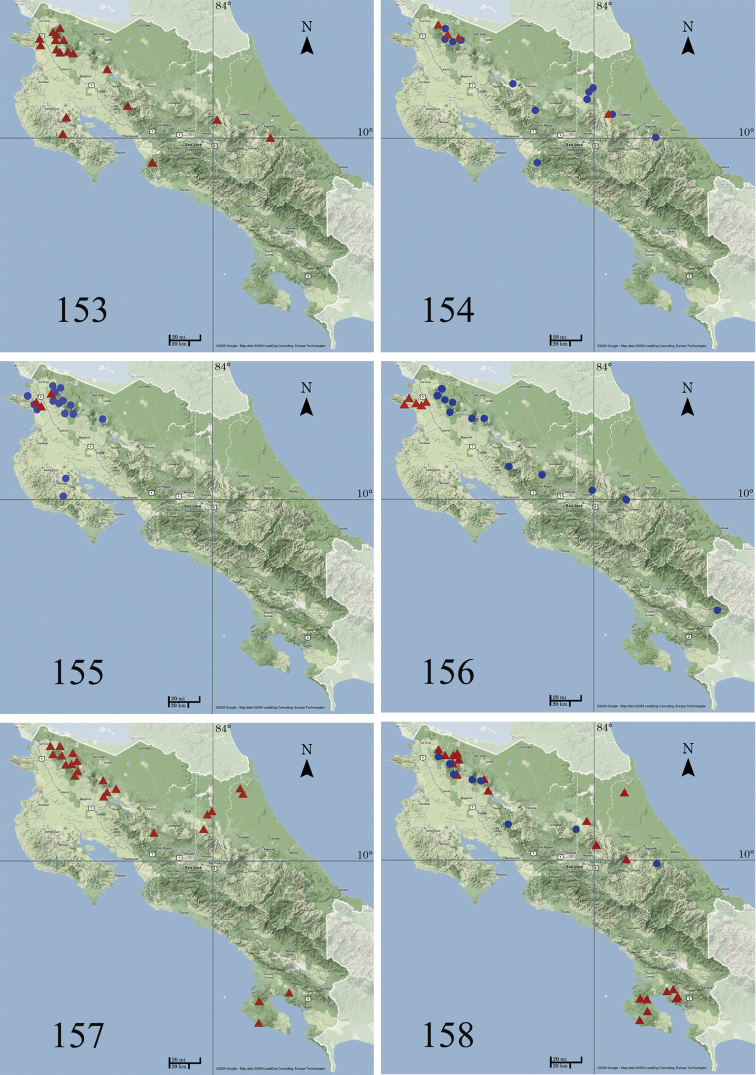
*Ethmia* collection localities in Costa Rica: **153** *Ethmia exornata*: red triangles **154** *Ethmia dianemillerae*: red triangles; *Ethmia adrianforsythi*: blue dots **155** *Ethmia phylacis*: red triangles; *Ethmia mnesicosma*: blue dots **156** *Ethmia chemsaki*: red triangles; *Ethmia stephenrumseyi*: blue dots **157** *Ethmia baliostola*: red triangles **158** *Ethmia berndkerni*: red triangles; *Ethmia dimauraorum*: blue dots.

**Figures 159–164.**
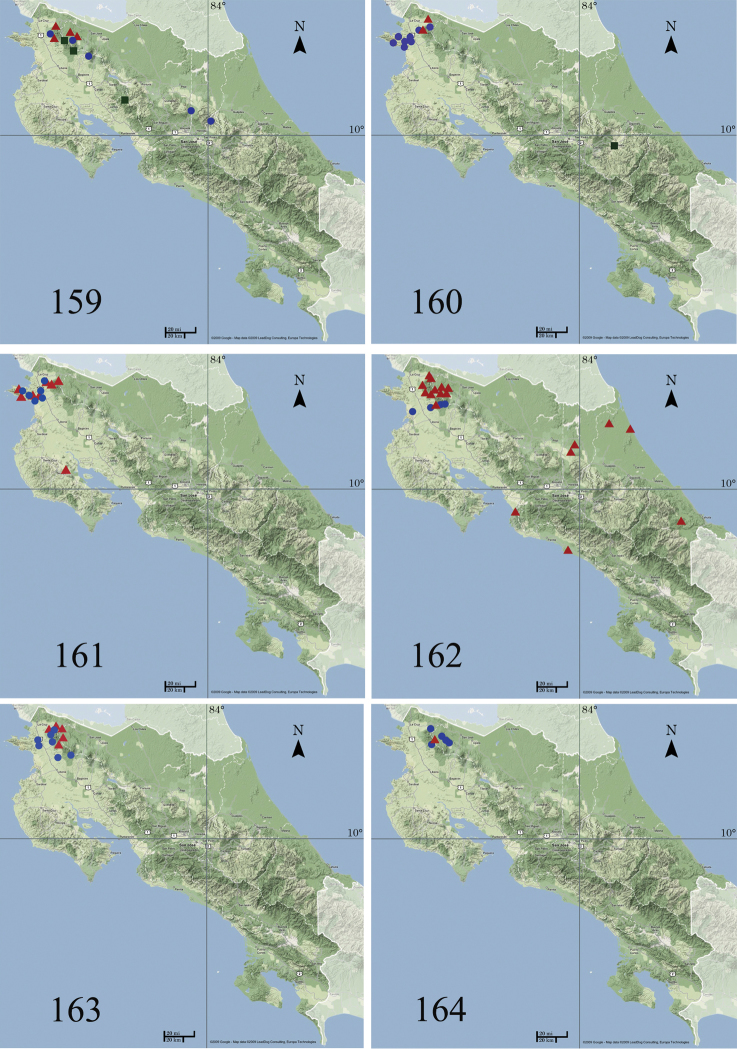
*Ethmia* collection localities in Costa Rica: **159** *Ethmia duckworthi*: red triangles; *Ethmia billalleni*: blue dots; *Ethmia ehakernae*: green squares **160** *Ethmia johnpringlei*: red triangles; *Ethmia helenmillerae*: blue dots; *Ethmia sandra*: green square **161** *Ethmia laphamorum*: red triangles; *Ethmia nigritaenia*: blue dots **162** *Ethmia catapeltica*: red triangles; *Ethmia petersterlingi*: blue dots **163** *Ethmia lesliesaulae*: red triangles; *Ethmia turnerorum*: blue dots **164** *Ethmia nicholsonorum*: red triangles; *Ethmia normgershenzi*: blue dots.

**Figures 165–168.**
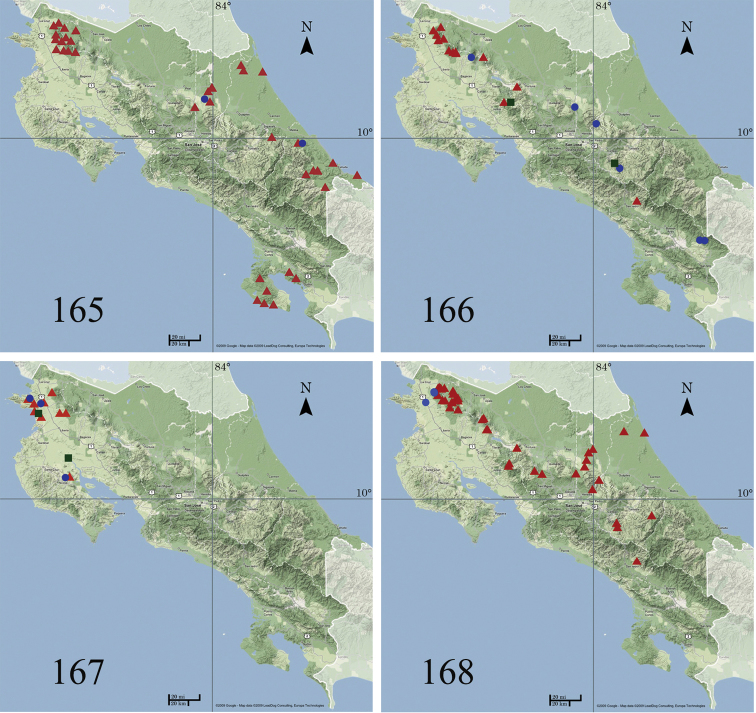
*Ethmia* collection localities in Costa Rica: **165** *Ethmia lichyi*: red triangles; *Ethmia hendersonorum*: blue dots **166** *Ethmia randyjonesi*: red triangles; *Ethmia transversella*: blue dots; *Ethmia randycurtisi*: green squares **167** *Ethmia miriamschulmanae*: red triangles; *Ethmia similatella*: blue dots; *Ethmia tilneyorum*: green squares **168** *Ethmia hammella*: red triangles; *Ethmia linda*: blue dots.

**Figure 169.**
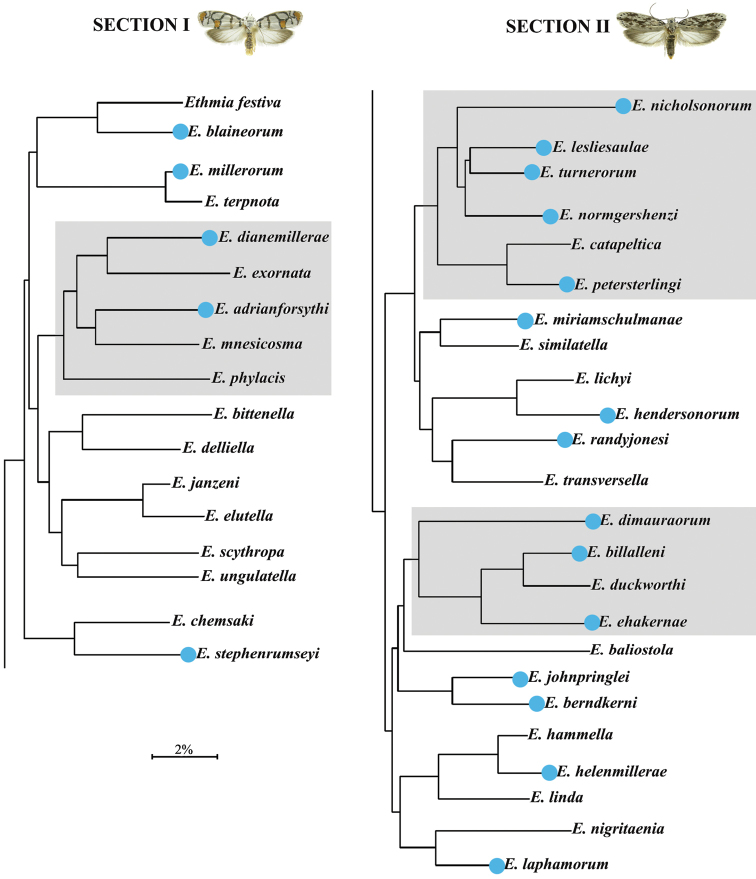
*Ethmia* from Costa Rica. Diagram based on a Neighbor-joining tree with a sample size of a randomly selected set of 4 specimens for each species. The tree is divided into Ethmia Section I and II following Powell’s division of the genus. Blue dots show new species described here. Shaded branches depict species complexes. See Suppl. material 3 for the NJ tree containing all barcoded samples (n=1122).

### DNA barcode sequences and divergence

Interspecific divergence in barcode sequences among these species of *Ethmia* was 1.75% or greater (Table 3), which approximates the 2% (or greater) divergence among previously recognized species that was encountered by Hebert et al. (2003) to delimit species and has been found to be commonplace in later years in ACG analyses (e.g., Janzen et al 2011). Intraspecific divergences for the same species were less than 1.6%. These values were used in combination with food plants, larval coloration, and adult morphology to determine what is a species of *Ethmia* in Costa Rica. By this method we encountered five new species within the Catalpeltica Species Complex (barcode divergence from the Nearest Neighbor species greater than 3.97%), two new species within the Exornata Species Complex (divergence greater than 6%) and 3 new species within the Duckworthi Species Complex (divergence greater than 4.1%).

From the 45 species found in Costa Rica, 42 occur in the ACG. Exceptions include: *Ethmia hendersonorum*, a species only recorded in the Caribbean lowlands (which are only very marginally present in ACG on its far northeastern corner), *Ethmia randycurtisi*, found above 1500 m in Cartago Province, and *Ethmia sandra* recorded only from the mid-elevation Caribbean rain forest near Turrialba, Cartago Province.

Within ACG the genus *Ethmia* is well amply present in dry and rain forest (Table 2). However, some species are usually found in dry forest: *Ethmia delliella*, *Ethmia bittenella*, *Ethmia festiva*, *Ethmia blaineorum*, *Ethmia elutella*, *Ethmia chemsaki*, *Ethmia helenmillerae*, *Ethmia johnpringlei*, *Ethmia nigritaenia*, *Ethmia laphamorum*, *Ethmia phylacis*, *Ethmia similatella* and *Ethmia tilneyorum*. A few occur in both rain and dry forest: *Ethmia exornata*, *Ethmia miriamschulmanae*, *Ethmia mnesicosma*, *Ethmia turnerorum* and *Ethmia ungulatella*, and 24 species (57%) have been found just in the rain forest. Thorough inventory of the southern half of Costa Rica is likely to reveal yet more Costa Rican species of *Ethmia* beyond the 45 now known.

## Supplementary Material

XML Treatment for Ethmia delliella

XML Treatment for Ethmia bittenella

XML Treatment for Ethmia festiva

XML Treatment for Ethmia blaineorum

XML Treatment for Ethmia scythropa

XML Treatment for Ethmia perpulchra

XML Treatment for Ethmia terpnota

XML Treatment for Ethmia millerorum

XML Treatment for Ethmia elutella

XML Treatment for Ethmia janzeni

XML Treatment for Ethmia ungulatella

XML Treatment for Ethmia exornata

XML Treatment for Ethmia dianemillerae

XML Treatment for Ethmia adrianforsythi

XML Treatment for Ethmia phylacis

XML Treatment for Ethmia mnesicosma

XML Treatment for Ethmia chemsaki

XML Treatment for Ethmia stephenrumseyi

XML Treatment for Ethmia baliostola

XML Treatment for Ethmia berndkerni

XML Treatment for Ethmia dimauraorum

XML Treatment for Ethmia duckworthi

XML Treatment for Ethmia billalleni

XML Treatment for Ethmia ehakernae

XML Treatment for Ethmia sandra

XML Treatment for Ethmia helenmillerae

XML Treatment for Ethmia johnpringlei

XML Treatment for Ethmia nigritaenia

XML Treatment for Ethmia laphamorum

XML Treatment for Ethmia catapeltica

XML Treatment for Ethmia petersterlingi

XML Treatment for Ethmia lesliesaulae

XML Treatment for Ethmia turnerorum

XML Treatment for Ethmia normgershenzi

XML Treatment for Ethmia nicholsonorum

XML Treatment for Ethmia lichyi

XML Treatment for Ethmia hendersonorum

XML Treatment for Ethmia transversella

XML Treatment for Ethmia randyjonesi

XML Treatment for Ethmia randycurtisi

XML Treatment for Ethmia miriamschulmanae

XML Treatment for Ethmia similatella

XML Treatment for Ethmia tilneyorum

XML Treatment for Ethmia hammella

XML Treatment for Ethmia linda

## Supplementary materials

Supplementary material 1 *Ethmia* from Costa Rica Data type: Collection localities data. Explanation note: Collection localities data. GenBank or BOLD accession numbers for CO1 sequences. DNA Barcoded specimens from Janzen & Hallwachs, and INBio. This dataset is made available under the Open Database License (http://opendatacommons.org/licenses/odbl/1.0/). The Open Database License (ODbL) is a license agreement intended to allow users to freely share, modify, and use this Dataset while maintaining this same freedom for others, provided that the original source and author(s) are credited.Eugenie Phillips-Rodríguez, Jerry A. Powell, Winnie Hallwachs, Daniel H. Janzen

Supplementary material 2 *Ethmia* from Costa Rica Data type: Collection localities data. Explanation note: Collection localities data. Additional material, INBio. This dataset is made available under the Open Database License (http://opendatacommons.org/licenses/odbl/1.0/). The Open Database License (ODbL) is a license agreement intended to allow users to freely share, modify, and use this Dataset while maintaining this same freedom for others, provided that the original source and author(s) are credited.Eugenie Phillips-Rodríguez, Jerry A. Powell, Winnie Hallwachs, Daniel H. Janzen

Supplementary material 3 *Ethmia* from Costa Rica Data type: NJtree (BOLD TaxonID Tree). This dataset is made available under the Open Database License (http://opendatacommons.org/licenses/odbl/1.0/). The Open Database License (ODbL) is a license agreement intended to allow users to freely share, modify, and use this Dataset while maintaining this same freedom for others, provided that the original source and author(s) are credited.Eugenie Phillips-Rodríguez, Jerry A. Powell, Winnie Hallwachs, Daniel H. Janzen
