# Supplementary material for: A synopsis of the genus Ethmia Hübner in Costa Rica: biology, distribution, and description of 22 new species (Lepidoptera, Gelechioidea, Depressariidae, Ethmiinae), with emphasis on the 42 species known from Área de Conservación Guanacaste
Source: Zookeys. 2014 Dec 9;(461):1–86. doi: 10.3897/zookeys.461.8377 (PMC4283717; doi:10.3897/zookeys.461.8377)
Supplement: Supplementary material 3 — Ethmia from Costa Rica [file zookeys-461-001-s003.pdf]

BOLD TaxonID Tree

```
Title            : SEARCH: Tax(Ethmia), Geo(Costa Rica) [SEARCH1]
Date             : 30-July-2014
Data Type        : Nucleotide
Distance Model   : Kimura 2 Parameter
Marker           : COI-5P
Codon Positions  : 1st, 2nd, 3rd
Labels           : SampleID, ProcessID
Filters          : Length > 200
Colorization     : [blue]=Stop Codons [red]=Contamination or misidentification
Attachment       : Photographs & Spreadsheet

Sequence Count   : 1122
Species count    : 41
Genus count      : 1
Family count     : 1
Unidentified     : 0
```

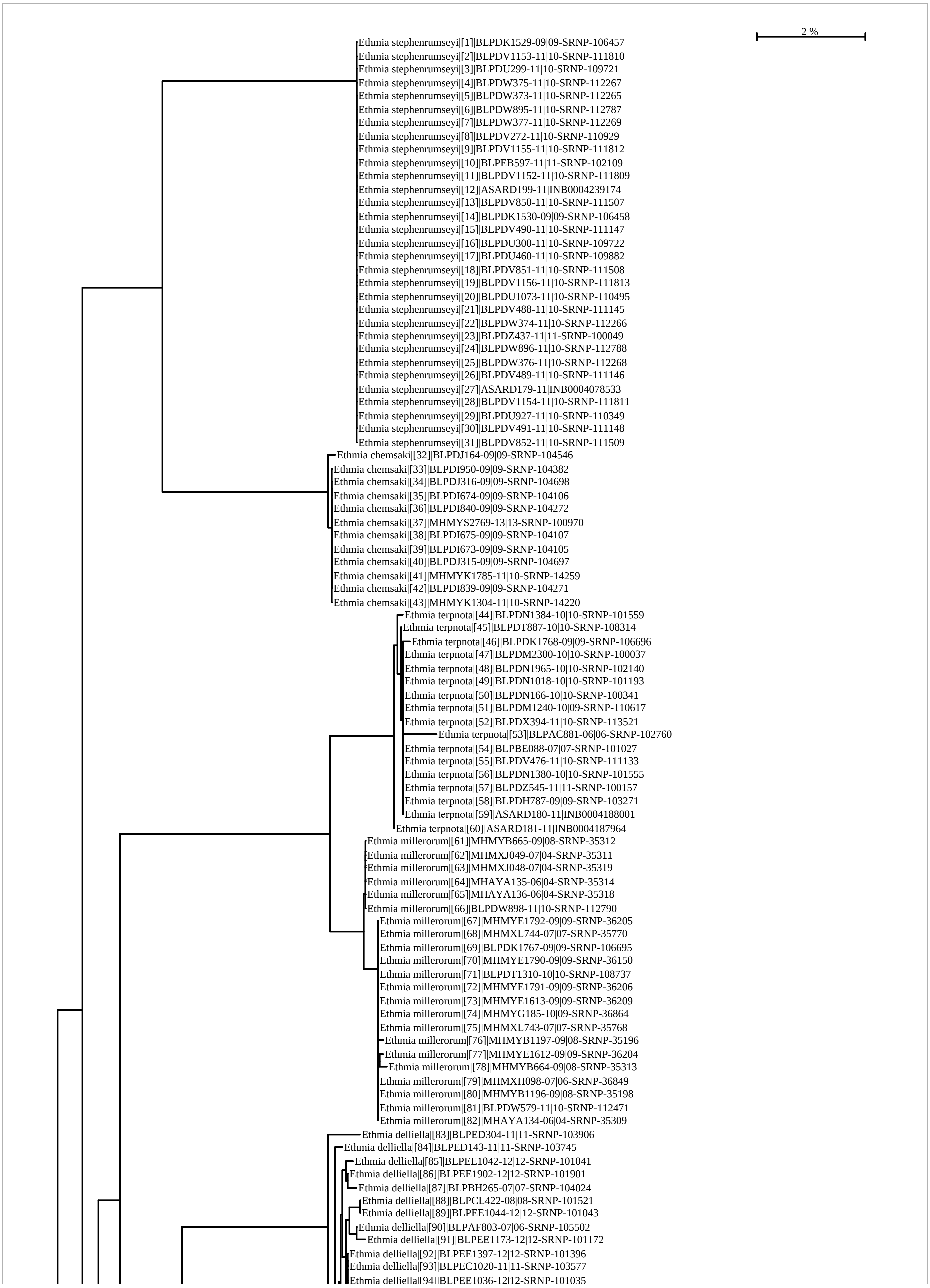
2 %
Ethmia stephenrumseyi|[1]|BLPDK1529-09|09-SRNP-106457
Ethmia stephenrumseyi|[2]|BLPDV1153-11|10-SRNP-111810
Ethmia stephenrumseyi|[3]|BLPDU299-11|10-SRNP-109721
Ethmia stephenrumseyi|[4]|BLPDW375-11|10-SRNP-112267
Ethmia stephenrumseyi|[5]|BLPDW373-11|10-SRNP-112265
Ethmia stephenrumseyi|[6]|BLPDW895-11|10-SRNP-112787
Ethmia stephenrumseyi|[7]|BLPDW377-11|10-SRNP-112269
Ethmia stephenrumseyi|[8]|BLPDV272-11|10-SRNP-110929
Ethmia stephenrumseyi|[9]|BLPDV1155-11|10-SRNP-111812
Ethmia stephenrumseyi|[10]|BLPEB597-11|11-SRNP-102109
Ethmia stephenrumseyi|[11]|BLPDV1152-11|10-SRNP-111809
Ethmia stephenrumseyi|[12]|ASARD199-11|INB0004239174
Ethmia stephenrumseyi|[13]|BLPDV850-11|10-SRNP-111507
Ethmia stephenrumseyi|[14]|BLPDK1530-09|09-SRNP-106458
Ethmia stephenrumseyi|[15]|BLPDV490-11|10-SRNP-111147
Ethmia stephenrumseyi|[16]|BLPDU300-11|10-SRNP-109722
Ethmia stephenrumseyi|[17]|BLPDU460-11|10-SRNP-109882
Ethmia stephenrumseyi|[18]|BLPDV851-11|10-SRNP-111508
Ethmia stephenrumseyi|[19]|BLPDV1156-11|10-SRNP-111813
Ethmia stephenrumseyi|[20]|BLPDU1073-11|10-SRNP-110495
Ethmia stephenrumseyi|[21]|BLPDV488-11|10-SRNP-111145
Ethmia stephenrumseyi|[22]|BLPDW374-11|10-SRNP-112266
Ethmia stephenrumseyi|[23]|BLPDZ437-11|11-SRNP-100049
Ethmia stephenrumseyi|[24]|BLPDW896-11|10-SRNP-112788
Ethmia stephenrumseyi|[25]|BLPDW376-11|10-SRNP-112268
Ethmia stephenrumseyi|[26]|BLPDV489-11|10-SRNP-111146
Ethmia stephenrumseyi|[27]|ASARD179-11|INB0004078533
Ethmia stephenrumseyi|[28]|BLPDV1154-11|10-SRNP-111811
Ethmia stephenrumseyi|[29]|BLPDU927-11|10-SRNP-110349
Ethmia stephenrumseyi|[30]|BLPDV491-11|10-SRNP-111148
Ethmia stephenrumseyi|[31]|BLPDV852-11|10-SRNP-111509
Ethmia chemsaki|[32]|BLPDJ164-09|09-SRNP-104546
Ethmia chemsaki|[33]|BLPDI950-09|09-SRNP-104382
Ethmia chemsaki|[34]|BLPDJ316-09|09-SRNP-104698
Ethmia chemsaki|[35]|BLPDI674-09|09-SRNP-104106
Ethmia chemsaki|[36]|BLPDI840-09|09-SRNP-104272
Ethmia chemsaki|[37]|MHMYS2769-13|13-SRNP-100970
Ethmia chemsaki|[38]|BLPDI675-09|09-SRNP-104107
Ethmia chemsaki|[39]|BLPDI673-09|09-SRNP-104105
Ethmia chemsaki|[40]|BLPDJ315-09|09-SRNP-104697
Ethmia chemsaki|[41]|MHMYK1785-11|10-SRNP-14259
Ethmia chemsaki|[42]|BLPDI839-09|09-SRNP-104271
Ethmia chemsaki|[43]|MHMYK1304-11|10-SRNP-14220
Ethmia terpnota|[44]|BLPDN1384-10|10-SRNP-101559
Ethmia terpnota|[45]|BLPDT887-10|10-SRNP-108314
Ethmia terpnota|[46]|BLPDK1768-09|09-SRNP-106696
Ethmia terpnota|[47]|BLPDM2300-10|10-SRNP-100037
Ethmia terpnota|[48]|BLPDN1965-10|10-SRNP-102140
Ethmia terpnota|[49]|BLPDN1018-10|10-SRNP-101193
Ethmia terpnota|[50]|BLPDN166-10|10-SRNP-100341
Ethmia terpnota|[51]|BLPDM1240-10|09-SRNP-110617
Ethmia terpnota|[52]|BLPDX394-11|10-SRNP-113521
Ethmia terpnota|[53]|BLPAC881-06|06-SRNP-102760
Ethmia terpnota|[54]|BLPBE088-07|07-SRNP-101027
Ethmia terpnota|[55]|BLPDV476-11|10-SRNP-111133
Ethmia terpnota|[56]|BLPDN1380-10|10-SRNP-101555
Ethmia terpnota|[57]|BLPDZ545-11|11-SRNP-100157
Ethmia terpnota|[58]|BLPDH787-09|09-SRNP-103271
Ethmia terpnota|[59]|ASARD180-11|INB0004188001
Ethmia terpnota|[60]|ASARD181-11|INB0004187964
Ethmia millerorum|[61]|MHMYB665-09|08-SRNP-35312
Ethmia millerorum|[62]|MHMXJ049-07|04-SRNP-35311
Ethmia millerorum|[63]|MHMXJ048-07|04-SRNP-35319
Ethmia millerorum|[64]|MHAYA135-06|04-SRNP-35314
Ethmia millerorum|[65]|MHAYA136-06|04-SRNP-35318
Ethmia millerorum|[66]|BLPDW898-11|10-SRNP-112790
Ethmia millerorum|[67]|MHMYE1792-09|09-SRNP-36205
Ethmia millerorum|[68]|MHMXL744-07|07-SRNP-35770
Ethmia millerorum|[69]|BLPDK1767-09|09-SRNP-106695
Ethmia millerorum|[70]|MHMYE1790-09|09-SRNP-36150
Ethmia millerorum|[71]|BLPDT1310-10|10-SRNP-108737
Ethmia millerorum|[72]|MHMYE1791-09|09-SRNP-36206
Ethmia millerorum|[73]|MHMYE1613-09|09-SRNP-36209
Ethmia millerorum|[74]|MHMYG185-10|09-SRNP-36864
Ethmia millerorum|[75]|MHMXL743-07|07-SRNP-35768
Ethmia millerorum|[76]|MHMYB1197-09|08-SRNP-35196
Ethmia millerorum|[77]|MHMYE1612-09|09-SRNP-36204
Ethmia millerorum|[78]|MHMYB664-09|08-SRNP-35313
Ethmia millerorum|[79]|MHMXH098-07|06-SRNP-36849
Ethmia millerorum|[80]|MHMYB1196-09|08-SRNP-35198
Ethmia millerorum|[81]|BLPDW579-11|10-SRNP-112471
Ethmia millerorum|[82]|MHAYA134-06|04-SRNP-35309
Ethmia delliella|[83]|BLPED304-11|11-SRNP-103906
Ethmia delliella|[84]|BLPED143-11|11-SRNP-103745
Ethmia delliella|[85]|BLPEE1042-12|12-SRNP-101041
Ethmia delliella|[86]|BLPEE1902-12|12-SRNP-101901
Ethmia delliella|[87]|BLPBH265-07|07-SRNP-104024
Ethmia delliella|[88]|BLPCL422-08|08-SRNP-101521
Ethmia delliella|[89]|BLPEE1044-12|12-SRNP-101043
Ethmia delliella|[90]|BLPAF803-07|06-SRNP-105502
Ethmia delliella|[91]|BLPEE1173-12|12-SRNP-101172
Ethmia delliella|[92]|BLPEE1397-12|12-SRNP-101396
Ethmia delliella|[93]|BLPEC1020-11|11-SRNP-103577
Ethmia delliella|[94]|BLPEE1036-12|12-SRNP-101035

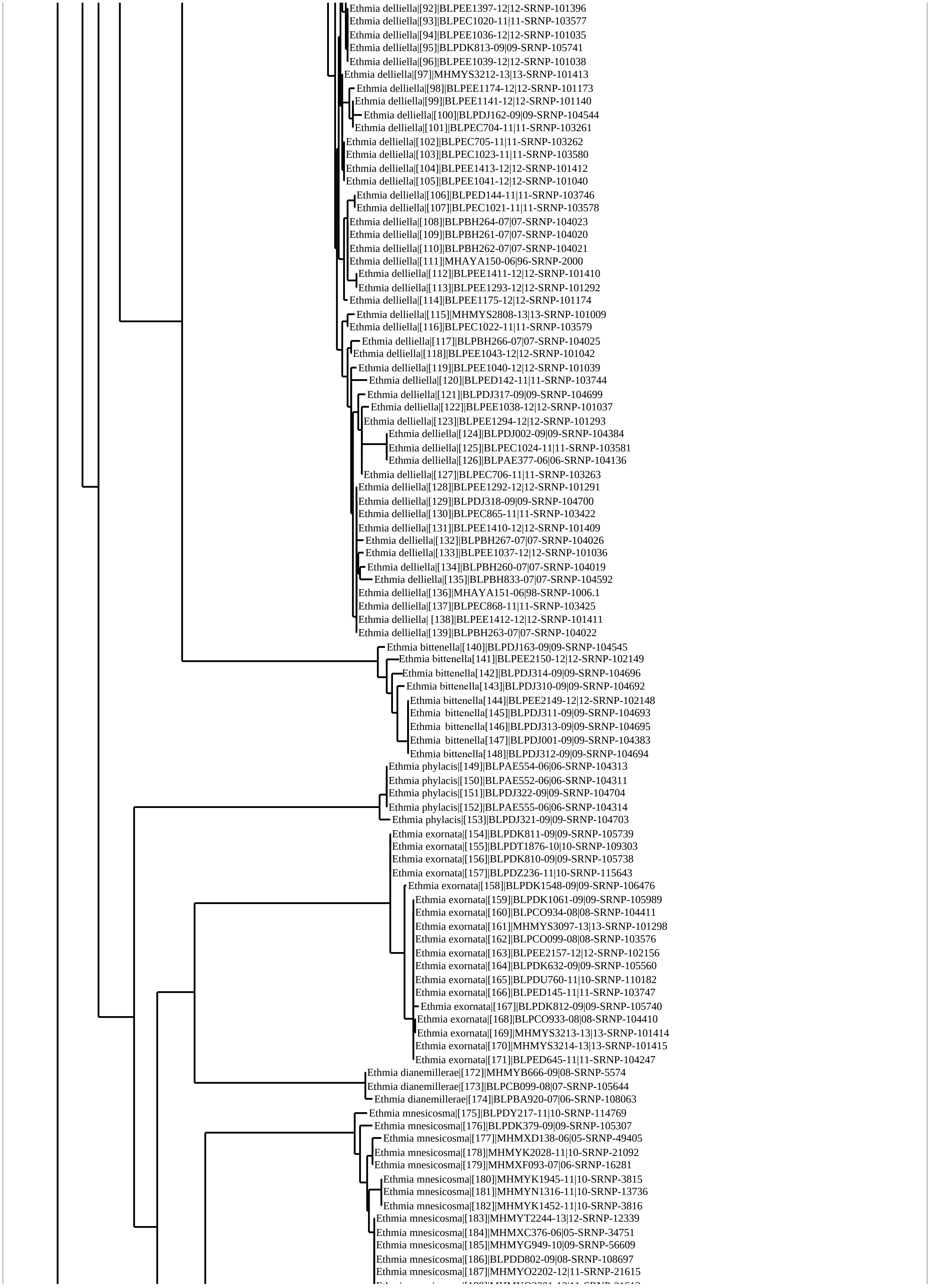
Ethmia delliella|[92]|BLPEE1397-12|12-SRNP-101396
Ethmia delliella|[93]|BLPEC1020-11|11-SRNP-103577
Ethmia delliella|[94]|BLPEE1036-12|12-SRNP-101035
Ethmia delliella|[95]|BLPDK813-09|09-SRNP-105741
Ethmia delliella|[96]|BLPEE1039-12|12-SRNP-101038
Ethmia delliella|[97]|MHMYS3212-13|13-SRNP-101413
Ethmia delliella|[98]|BLPEE1174-12|12-SRNP-101173
Ethmia delliella|[99]|BLPEE1141-12|12-SRNP-101140
Ethmia delliella|[100]|BLPDJ162-09|09-SRNP-104544
Ethmia delliella|[101]|BLPEC704-11|11-SRNP-103261
Ethmia delliella|[102]|BLPEC705-11|11-SRNP-103262
Ethmia delliella|[103]|BLPEC1023-11|11-SRNP-103580
Ethmia delliella|[104]|BLPEE1413-12|12-SRNP-101412
Ethmia delliella|[105]|BLPEE1041-12|12-SRNP-101040
Ethmia delliella|[106]|BLPED144-11|11-SRNP-103746
Ethmia delliella|[107]|BLPEC1021-11|11-SRNP-103578
Ethmia delliella|[108]|BLPBH264-07|07-SRNP-104023
Ethmia delliella|[109]|BLPBH261-07|07-SRNP-104020
Ethmia delliella|[110]|BLPBH262-07|07-SRNP-104021
Ethmia delliella|[111]|MHAYA150-06|96-SRNP-2000
Ethmia delliella|[112]|BLPEE1411-12|12-SRNP-101410
Ethmia delliella|[113]|BLPEE1293-12|12-SRNP-101292
Ethmia delliella|[114]|BLPEE1175-12|12-SRNP-101174
Ethmia delliella|[115]|MHMYS2808-13|13-SRNP-101009
Ethmia delliella|[116]|BLPEC1022-11|11-SRNP-103579
Ethmia delliella|[117]|BLPBH266-07|07-SRNP-104025
Ethmia delliella|[118]|BLPEE1043-12|12-SRNP-101042
Ethmia delliella|[119]|BLPEE1040-12|12-SRNP-101039
Ethmia delliella|[120]|BLPED142-11|11-SRNP-103744
Ethmia delliella|[121]|BLPDJ317-09|09-SRNP-104699
Ethmia delliella|[122]|BLPEE1038-12|12-SRNP-101037
Ethmia delliella|[123]|BLPEE1294-12|12-SRNP-101293
Ethmia delliella|[124]|BLPDJ002-09|09-SRNP-104384
Ethmia delliella|[125]|BLPEC1024-11|11-SRNP-103581
Ethmia delliella|[126]|BLPAE377-06|06-SRNP-104136
Ethmia delliella|[127]|BLPEC706-11|11-SRNP-103263
Ethmia delliella|[128]|BLPEE1292-12|12-SRNP-101291
Ethmia delliella|[129]|BLPDJ318-09|09-SRNP-104700
Ethmia delliella|[130]|BLPEC865-11|11-SRNP-103422
Ethmia delliella|[131]|BLPEE1410-12|12-SRNP-101409
Ethmia delliella|[132]|BLPBH267-07|07-SRNP-104026
Ethmia delliella|[133]|BLPEE1037-12|12-SRNP-101036
Ethmia delliella|[134]|BLPBH260-07|07-SRNP-104019
Ethmia delliella|[135]|BLPBH833-07|07-SRNP-104592
Ethmia delliella|[136]|MHAYA151-06|98-SRNP-1006.1
Ethmia delliella|[137]|BLPEC868-11|11-SRNP-103425
Ethmia delliella| [138]|BLPEE1412-12|12-SRNP-101411
Ethmia delliella|[139]|BLPBH263-07|07-SRNP-104022
Ethmia bittenella|[140]|BLPDJ163-09|09-SRNP-104545
Ethmia bittenella[141]|BLPEE2150-12|12-SRNP-102149
Ethmia bittenella|[142]|BLPDJ314-09|09-SRNP-104696
Ethmia bittenella[143]|BLPDJ310-09|09-SRNP-104692
Ethmia bittenella[144]|BLPEE2149-12|12-SRNP-102148
Ethmia bittenella[145]|BLPDJ311-09|09-SRNP-104693
Ethmia bittenella[146]|BLPDJ313-09|09-SRNP-104695
Ethmia bittenella[147]|BLPDJ001-09|09-SRNP-104383
Ethmia bittenella[148]|BLPDJ312-09|09-SRNP-104694
Ethmia phylacis|[149]|BLPAE554-06|06-SRNP-104313
Ethmia phylacis|[150]|BLPAE552-06|06-SRNP-104311
Ethmia phylacis|[151]|BLPDJ322-09|09-SRNP-104704
Ethmia phylacis|[152]|BLPAE555-06|06-SRNP-104314
Ethmia phylacis|[153]|BLPDJ321-09|09-SRNP-104703
Ethmia exornata|[154]|BLPDK811-09|09-SRNP-105739
Ethmia exornata|[155]|BLPDT1876-10|10-SRNP-109303
Ethmia exornata|[156]|BLPDK810-09|09-SRNP-105738
Ethmia exornata|[157]|BLPDZ236-11|10-SRNP-115643
Ethmia exornata|[158]|BLPDK1548-09|09-SRNP-106476
Ethmia exornata|[159]|BLPDK1061-09|09-SRNP-105989
Ethmia exornata|[160]|BLPCO934-08|08-SRNP-104411
Ethmia exornata|[161]|MHMYS3097-13|13-SRNP-101298
Ethmia exornata|[162]|BLPCO099-08|08-SRNP-103576
Ethmia exornata|[163]|BLPEE2157-12|12-SRNP-102156
Ethmia exornata|[164]|BLPDK632-09|09-SRNP-105560
Ethmia exornata|[165]|BLPDU760-11|10-SRNP-110182
Ethmia exornata|[166]|BLPED145-11|11-SRNP-103747
Ethmia exornata|[167]|BLPDK812-09|09-SRNP-105740
Ethmia exornata|[168]|BLPCO933-08|08-SRNP-104410
Ethmia exornata|[169]|MHMYS3213-13|13-SRNP-101414
Ethmia exornata|[170]|MHMYS3214-13|13-SRNP-101415
Ethmia exornata|[171]|BLPED645-11|11-SRNP-104247
Ethmia dianemillerae|[172]|MHMYB666-09|08-SRNP-5574
Ethmia dianemillerae|[173]|BLPCB099-08|07-SRNP-105644
Ethmia dianemillerae|[174]|BLPBA920-07|06-SRNP-108063
Ethmia mnesicosma|[175]|BLPDY217-11|10-SRNP-114769
Ethmia mnesicosma|[176]|BLPDK379-09|09-SRNP-105307
Ethmia mnesicosma|[177]|MHMXD138-06|05-SRNP-49405
Ethmia mnesicosma|[178]|MHMYK2028-11|10-SRNP-21092
Ethmia mnesicosma|[179]|MHMXF093-07|06-SRNP-16281
Ethmia mnesicosma|[180]|MHMYK1945-11|10-SRNP-3815
Ethmia mnesicosma|[181]|MHMYN1316-11|10-SRNP-13736
Ethmia mnesicosma|[182]|MHMYK1452-11|10-SRNP-3816
Ethmia mnesicosma|[183]|MHMYT2244-13|12-SRNP-12339
Ethmia mnesicosma|[184]|MHMXC376-06|05-SRNP-34751
Ethmia mnesicosma|[185]|MHMYG949-10|09-SRNP-56609
Ethmia mnesicosma|[186]|BLPDD802-09|08-SRNP-108697
Ethmia mnesicosma|[187]|MHMYO2202-12|11-SRNP-21615

Ethmia mnesicosma|[186]|BLPDD802-09|08-SRNP-108697
Ethmia mnesicosma|[187]|MHMYO2202-12|11-SRNP-21615
Ethmia mnesicosma|[188]|MHMYO2201-12|11-SRNP-21612
Ethmia mnesicosma|[189]|MHMXC370-06|05-SRNP-34752
Ethmia mnesicosma|[190]|MHMYG960-10|09-SRNP-56617
Ethmia mnesicosma|[191]|MHMYG958-10|09-SRNP-56618
Ethmia mnesicosma|[192]|MHMYG959-10|09-SRNP-56610
Ethmia mnesicosma|[193]|BLPED1049-12|11-SRNP-104651
Ethmia mnesicosma|[194]|MHMYK339-11|10-SRNP-3842
Ethmia mnesicosma|[195]|MHMYK341-11|10-SRNP-3851
Ethmia mnesicosma|[196]|MHMYK697-11|10-SRNP-3888
Ethmia mnesicosma|[197]|MHMYK1947-11|10-SRNP-3856
Ethmia mnesicosma|[198]|MHMYK1944-11|10-SRNP-3873
Ethmia mnesicosma|[199]|MHMYK529-11|10-SRNP-3869
Ethmia mnesicosma|[200]|MHMYK340-11|10-SRNP-3866
Ethmia mnesicosma|[201]|BLPDG379-09|09-SRNP-101923
Ethmia mnesicosma|[202]|BLPDZ113-11|10-SRNP-115520
Ethmia mnesicosma|[203]|BLPDK631-09|09-SRNP-105559
Ethmia mnesicosma|[204]|MHMYK338-11|10-SRNP-3698
Ethmia mnesicosma|[205]|MHMYS241-12|11-SRNP-56081
Ethmia mnesicosma|[206]|MHMXF091-07|06-SRNP-16295
Ethmia mnesicosma|[207]|MHMXF095-07|06-SRNP-16314
Ethmia mnesicosma|[208]|BLPCG716-08|07-SRNP-110961
Ethmia mnesicosma|[209]|MHMXF092-07|06-SRNP-16307
Ethmia mnesicosma|[210]|BLPDL1862-10|09-SRNP-109244
Ethmia mnesicosma|[211]|BLPAF804-07|06-SRNP-105503
Ethmia mnesicosma|[212]|MHMYT2243-13|12-SRNP-12299
Ethmia mnesicosma|[213]|MHMYK1273-11|10-SRNP-22094
Ethmia mnesicosma|[214]|BLPEA500-11|11-SRNP-100967
Ethmia mnesicosma|[215]|MHMYB1198-09|07-SRNP-22083
Ethmia mnesicosma|[216]|BLPDZ235-11|10-SRNP-115642
Ethmia mnesicosma|[217]|MHAYA149-06|04-SRNP-45698
Ethmia mnesicosma|[218]|MHAYA147-06|04-SRNP-45695
Ethmia mnesicosma|[219]|MHMYB1199-09|07-SRNP-22080
Ethmia mnesicosma|[220]|MHAYA148-06|04-SRNP-45699
Ethmia mnesicosma|[221]|MHMXF094-07|06-SRNP-16419
Ethmia adrianforsythi|[222]|ASARD183-11|INB0003230733
Ethmia adrianforsythi|[223]|ASARD182-11|INB0003231101
Ethmia adrianforsythi|[224]|ASARD184-11|INB0003230742
Ethmia adrianforsythi|[225]|BLPEB1014-11|11-SRNP-102526
Ethmia adrianforsythi|[226]|BLPDQ395-10|10-SRNP-105021
Ethmia adrianforsythi|[227]|BLPDR927-10|10-SRNP-106552
Ethmia ungulatella|[228]|MHMYS3250-13|13-SRNP-101451
Ethmia ungulatella|[229]|MHMYS3372-13|13-SRNP-101573
Ethmia ungulatella|[230]|MHMYS3078-13|13-SRNP-101279
Ethmia ungulatella|[231]|BLPDM1357-10|09-SRNP-110734
Ethmia ungulatella|[232]|MHMYS2868-13|13-SRNP-101069
Ethmia ungulatella|[233]|MHMYS3252-13|13-SRNP-101453
Ethmia ungulatella|[234]|BLPDM2195-10|09-SRNP-111572
Ethmia ungulatella|[235]|BLPDZ348-11|10-SRNP-115755
Ethmia ungulatella|[236]|MHMYS3251-13|13-SRNP-101452
Ethmia ungulatella|[237]|BLPCC242-08|07-SRNP-106727
Ethmia ungulatella|[238]|BLPCF910-08|07-SRNP-110215
Ethmia ungulatella|[239]|BLPDK1084-09|09-SRNP-106012
Ethmia ungulatella|[240]|LTOL1261-12|DA-03-3337
Ethmia ungulatella|[241]|MHMYS3313-13|13-SRNP-101514
Ethmia ungulatella|[242]|BLPCG349-08|07-SRNP-110594
Ethmia ungulatella|[243]|BLPDU007-11|10-SRNP-109429
Ethmia scythropa|[244]|BLPCM163-08|08-SRNP-101760
Ethmia scythropa|[245]|BLPAD182-06|06-SRNP-103001
Ethmia scythropa|[246]|MHAYA143-06|05-SRNP-22159
Ethmia scythropa|[247]|BLPDB220-09|08-SRNP-106235
Ethmia scythropa|[248]|BLPEB1016-11|11-SRNP-102528
Ethmia scythropa|[249]|BLPCD760-08|07-SRNP-108185
Ethmia scythropa|[250]|BLPDF015-09|09-SRNP-100619
Ethmia scythropa|[251]|BLPCD759-08|07-SRNP-108184
Ethmia scythropa|[252]|MHAYA137-06|05-SRNP-22148
Ethmia scythropa|[253]|MHAYA139-06|02-SRNP-16140
Ethmia scythropa|[254]|BLPCF307-08|07-SRNP-109612
Ethmia scythropa|[255]|BLPDS582-10|10-SRNP-107206
Ethmia scythropa|[256]|BLPDF013-09|09-SRNP-100617
Ethmia scythropa|[257]|MHAYA138-06|05-SRNP-22150
Ethmia scythropa|[258]|BLPBC007-07|06-SRNP-109030
Ethmia scythropa|[259]|BLPDS213-10|10-SRNP-106837
Ethmia scythropa|[260]|BLPBE086-07|07-SRNP-101025
Ethmia scythropa|[261]|BLPDU645-11|10-SRNP-110067
Ethmia scythropa|[262]|BLPDK2155-09|09-SRNP-107083
Ethmia scythropa|[263]|BLPDM1397-10|09-SRNP-110774
Ethmia scythropa|[264]|BLPED564-11|11-SRNP-104166
Ethmia scythropa|[265]|BLPCJ938-08|07-SRNP-114003
Ethmia scythropa|[266]|BLPBD481-07|07-SRNP-100480
Ethmia scythropa|[267]|BLPDS219-10|10-SRNP-106843
Ethmia scythropa|[268]|BLPDR564-10|10-SRNP-106189
Ethmia scythropa|[269]|BLPDC804-09|08-SRNP-107759
Ethmia scythropa|[270]|BLPAG532-07|06-SRNP-106171
Ethmia scythropa|[271]|BLPDM769-10|09-SRNP-110146
Ethmia scythropa|[272]|BLPDZ436-11|11-SRNP-100048
Ethmia scythropa|[273]|BLPDM979-10|09-SRNP-110356
Ethmia scythropa|[274]|BLPDS583-10|10-SRNP-107207
Ethmia scythropa|[275]|BLPDU667-11|10-SRNP-110089
Ethmia scythropa|[276]|BLPDW897-11|10-SRNP-112789
Ethmia scythropa|[277]|BLPCO541-08|08-SRNP-104018
Ethmia scythropa|[278]|BLPDS581-10|10-SRNP-107205
Ethmia scythropa|[279]|BLPDM1148-10|09-SRNP-110525
Ethmia scythropa|[280]|MHAYA141-06|05-SRNP-22123
Ethmia scythropa|[281]|BLPDV1158-11|10-SRNP-111815

Ethmia scythropa|[279]|BLPDM1148-10|09-SRNP-110525
Ethmia scythropa|[280]|MHAYA141-06|05-SRNP-22123
Ethmia scythropa|[281]|BLPDV1158-11|10-SRNP-111815
Ethmia scythropa|[282]|BLPBE087-07|07-SRNP-101026
Ethmia scythropa|[283]|BLPAD266-06|06-SRNP-103085
Ethmia scythropa|[284]|BLPDG053-09|09-SRNP-101597
Ethmia scythropa|[285]|BLPDU298-11|10-SRNP-109720
Ethmia scythropa|[286]|BLPED646-11|11-SRNP-104248
Ethmia scythropa|[287]|BLPCF306-08|07-SRNP-109611
Ethmia scythropa|[288]|BLPDS659-10|10-SRNP-107283
Ethmia scythropa|[289]|MHMXF072-07|06-SRNP-31571
Ethmia scythropa|[290]|MHMXF071-07|06-SRNP-31481
Ethmia scythropa|[291]|BLPCP260-08|08-SRNP-104677
Ethmia scythropa|[292]|BLPDM1147-10|09-SRNP-110524
Ethmia scythropa|[293]|BLPEB755-11|11-SRNP-102267
Ethmia scythropa|[294]|BLPDN986-10|10-SRNP-101161
Ethmia scythropa|[295]|BLPDM768-10|09-SRNP-110145
Ethmia scythropa|[296]|BLPCK141-08|08-SRNP-100234
Ethmia scythropa|[297]|BLPEC103-11|11-SRNP-102660
Ethmia scythropa|[298]|BLPEC100-11|11-SRNP-102657
Ethmia scythropa|[299]|BLPAC880-06|06-SRNP-102759
Ethmia scythropa|[300]|MHMXF074-07|06-SRNP-31486
Ethmia scythropa|[301]|MHAYA140-06|02-SRNP-16119
Ethmia scythropa|[302]|BLPED563-11|11-SRNP-104165
Ethmia scythropa|[303]|BLPDN2289-10|10-SRNP-102464
Ethmia scythropa|[304]|BLPCF305-08|07-SRNP-109610
Ethmia scythropa|[305]|BLPCG645-08|07-SRNP-110890
Ethmia scythropa|[306]|MHAYA146-06|03-SRNP-15570
Ethmia scythropa|[307]|BLPCD761-08|07-SRNP-108186
Ethmia scythropa|[308]|BLPEB045-11|11-SRNP-101557
Ethmia scythropa|[309]|BLPDN1378-10|10-SRNP-101553
Ethmia scythropa|[310]|BLPDM1012-10|09-SRNP-110389
Ethmia scythropa|[311]|BLPDU761-11|10-SRNP-110183
Ethmia scythropa|[312]|MHMXF073-07|06-SRNP-31484
Ethmia scythropa|[313]|BLPDC140-09|08-SRNP-107095
Ethmia scythropa|[314]|MHAYA145-06|05-SRNP-22416.1
Ethmia scythropa|[315]|MHAYA142-06|05-SRNP-22638
Ethmia scythropa|[316]|BLPDU358-11|10-SRNP-109780
Ethmia scythropa|[317]|BLPBD482-07|07-SRNP-100481
Ethmia scythropa|[318]|MHMYB1194-09|08-SRNP-21493
Ethmia scythropa|[319]|BLPAD183-06|06-SRNP-103002
Ethmia scythropa|[320]|MHAYA144-06|05-SRNP-22063
Ethmia festiva|[321]|BLPDI841-09|09-SRNP-104273
Ethmia festiva|[322]|BLPED1171-12|11-SRNP-104773
Ethmia festiva|[323]|BLPED687-11|11-SRNP-104289
Ethmia festiva|[324]|BLPDV1210-11|10-SRNP-111867
Ethmia festiva|[325]|BLPDJ004-09|09-SRNP-104386
Ethmia festiva|[326]|BLPEC866-11|11-SRNP-103423
Ethmia festiva|[327]|BLPDL610-10|09-SRNP-107992
Ethmia festiva|[328]|BLPED141-11|11-SRNP-103743
Ethmia festiva|[329]|BLPEC867-11|11-SRNP-103424
Ethmia festiva|[330]|BLPEC869-11|11-SRNP-103426
Ethmia festiva|[331]|BLPED2315-12|11-SRNP-105917
Ethmia festiva|[332]|BLPEC1019-11|11-SRNP-103576
Ethmia festiva|[333]|BLPDR780-10|10-SRNP-106405
Ethmia festiva|[334]|BLPDV1194-11|10-SRNP-111851
Ethmia festiva|[335]|BLPED609-11|11-SRNP-104211
Ethmia festiva|[336]|BLPED340-11|11-SRNP-103942
Ethmia festiva|[337]|BLPED1172-12|11-SRNP-104774
Ethmia festiva|[338]|BLPDT256-10|10-SRNP-107683
Ethmia blaineorum|[339]|BLPED140-11|11-SRNP-103742
Ethmia blaineorum|[340]|BLPAE546-06|06-SRNP-104305
Ethmia blaineorum|[341]|BLPDJ320-09|09-SRNP-104702
Ethmia blaineorum|[342]|BLPAE544-06|06-SRNP-104303
Ethmia blaineorum|[343]|BLPDJ005-09|09-SRNP-104387
Ethmia blaineorum|[344]|BLPDJ044-09|09-SRNP-104426
Ethmia blaineorum|[345]|BLPAE548-06|06-SRNP-104307
Ethmia blaineorum|[346]|BLPAE545-06|06-SRNP-104304
Ethmia blaineorum|[347]|BLPAE547-06|06-SRNP-104306
Ethmia blaineorum|[348]|BLPCA747-08|07-SRNP-105352
Ethmia blaineorum|[349]|BLPDJ319-09|09-SRNP-104701
Ethmia blaineorum|[350]|BLPDJ460-09|09-SRNP-104842
Ethmia blaineorum|[351]|BLPDI873-09|09-SRNP-104305
Ethmia elutella|[352]|BLPDS249-10|10-SRNP-106873
Ethmia elutella|[353]|BLPEB240-11|11-SRNP-101752
Ethmia elutella|[354]|BLPDS687-10|10-SRNP-107311
Ethmia elutella|[355]|BLPDF847-09|09-SRNP-101451
Ethmia elutella|[356]|BLPDG291-09|09-SRNP-101835
Ethmia elutella|[357]|BLPDS191-10|10-SRNP-106815
Ethmia elutella|[358]|BLPEB242-11|11-SRNP-101754
Ethmia elutella|[359]|BLPEB139-11|11-SRNP-101651
Ethmia elutella|[360]|BLPEB647-11|11-SRNP-102159
Ethmia elutella|[361]|BLPDG549-09|09-SRNP-102093
Ethmia elutella|[362]|BLPDR932-10|10-SRNP-106557
Ethmia janzeni|[363]|BLPEA494-11|11-SRNP-100961
Ethmia janzeni|[364]|BLPEB241-11|11-SRNP-101753
Ethmia janzeni|[365]|BLPEE245-12|12-SRNP-100244
Ethmia janzeni|[366]|BLPEB164-11|11-SRNP-101676
Ethmia janzeni|[367]|BLPEA496-11|11-SRNP-100963
Ethmia janzeni|[368]|BLPEA498-11|11-SRNP-100965
Ethmia janzeni|[369]|BLPEE247-12|12-SRNP-100246
Ethmia janzeni|[370]|BLPDR841-10|10-SRNP-106466
Ethmia janzeni|[371]|BLPEA497-11|11-SRNP-100964
Ethmia janzeni|[372]|BLPEA492-11|11-SRNP-100959
Ethmia janzeni|[373]|BLPDF781-09|09-SRNP-101385
Ethmia janzeni|[374]|BLPDR689-10|10-SRNP-106314
Ethmia janzeni|[375]|BLPDH087-09|09-SRNP-102571

Ethmia janzeni|[372]|BLPEA492-11|11-SRNP-100959
Ethmia janzeni|[373]|BLPDF781-09|09-SRNP-101385
Ethmia janzeni|[374]|BLPDR689-10|10-SRNP-106314
Ethmia janzeni|[375]|BLPDH087-09|09-SRNP-102571
Ethmia janzeni|[376]|BLPEE407-12|12-SRNP-100406
Ethmia janzeni|[377]|BLPDR883-10|10-SRNP-106508
Ethmia janzeni|[378]|BLPEE409-12|12-SRNP-100408
Ethmia janzeni|[379]|BLPDR787-10|10-SRNP-106412
Ethmia janzeni|[380]|BLPDG100-09|09-SRNP-101644
Ethmia janzeni|[381]|BLPEE406-12|12-SRNP-100405
Ethmia janzeni|[382]|BLPEA493-11|11-SRNP-100960
Ethmia janzeni|[383]|BLPEB239-11|11-SRNP-101751
Ethmia janzeni|[384]|BLPEE248-12|12-SRNP-100247
Ethmia janzeni|[385]|BLPEE408-12|12-SRNP-100407
Ethmia janzeni|[386]|BLPDR902-10|10-SRNP-106527
Ethmia janzeni|[387]|BLPEE246-12|12-SRNP-100245
Ethmia janzeni|[388]|BLPEE244-12|12-SRNP-100243
Ethmia janzeni|[389]|BLPEA495-11|11-SRNP-100962
Ethmia hammella|[390]|BLPDO542-10|10-SRNP-103124
Ethmia hammella|[391]|BLPDM896-10|09-SRNP-110273
Ethmia hammella|[392]|BLPBD484-07|07-SRNP-100483
Ethmia hammella|[393]|BLPDC139-09|08-SRNP-107094
Ethmia hammella|[394]|BLPDX393-11|10-SRNP-113520
Ethmia hammella|[395]|BLPBD485-07|07-SRNP-100484
Ethmia hammella|[396]|BLPDM1437-10|09-SRNP-110814
Ethmia hammella|[397]|BLPDM1428-10|09-SRNP-110805
Ethmia hammella|[398]|BLPDM1254-10|09-SRNP-110631
Ethmia hammella|[399]|BLPDO298-10|10-SRNP-102880
Ethmia hammella|[400]|BLPDN2198-10|10-SRNP-102373
Ethmia hammella|[401]|BLPBG882-07|07-SRNP-103701
Ethmia hammella|[402]|BLPDS221-10|10-SRNP-106845
Ethmia hammella|[403]|BLPBE069-07|07-SRNP-101008
Ethmia hammella|[404]|ASARD373-11|INB0004263680
Ethmia hammella|[405]|ASARD427-11|INB0004269506
Ethmia hammella|[406]|BLPDK251-09|09-SRNP-105179
Ethmia hammella|[407]|BLPED610-11|11-SRNP-104212
Ethmia hammella|[408]|BLPDD179-09|08-SRNP-108074
Ethmia hammella|[409]|BLPDN1945-10|10-SRNP-102120
Ethmia hammella|[410]|BLPEB051-11|11-SRNP-101563
Ethmia hammella|[411]|BLPDM1078-10|09-SRNP-110455
Ethmia hammella|[412]|BLPDM2267-10|10-SRNP-100004
Ethmia hammella|[413]|ASARD144-11|INB0004238428
Ethmia hammella|[414]|BLPDN226-10|10-SRNP-100401
Ethmia hammella|[415]|ASARD177-11|INB0003230769
Ethmia hammella|[416]|BLPDD180-09|08-SRNP-108075
Ethmia hammella|[417]|BLPDN1933-10|10-SRNP-102108
Ethmia hammella|[418]|BLPDS679-10|10-SRNP-107303
Ethmia hammella|[419]|BLPDW478-11|10-SRNP-112370
Ethmia hammella|[420]|BLPDK1612-09|09-SRNP-106540
Ethmia hammella|[421]|BLPDO175-10|10-SRNP-102757
Ethmia hammella|[422]|BLPEB1032-11|11-SRNP-102544
Ethmia hammella|[423]|BLPDK1656-09|09-SRNP-106584
Ethmia hammella|[424]|BLPDM840-10|09-SRNP-110217
Ethmia hammella|[425]|BLPAD333-06|06-SRNP-103152
Ethmia hammella|[426]|BLPEB603-11|11-SRNP-102115
Ethmia hammella|[427]|ASARD140-11|INB0004238424
Ethmia hammella|[428]|BLPDM976-10|09-SRNP-110353
Ethmia hammella|[429]|BLPDK1625-09|09-SRNP-106553
Ethmia hammella|[430]|BLPDK1645-09|09-SRNP-106573
Ethmia hammella|[431]|ASARD175-11|INB0004166236
Ethmia hammella|[432]|BLPDW012-11|10-SRNP-111904
Ethmia hammella|[433]|BLPDK1753-09|09-SRNP-106681
Ethmia hammella|[434]|BLPBA768-07|06-SRNP-107911
Ethmia hammella|[435]|BLPDN1990-10|10-SRNP-102165
Ethmia hammella|[436]|ASARD371-11|INB0004263678
Ethmia hammella|[437]|BLPDL969-10|09-SRNP-108351
Ethmia hammella|[438]|BLPDN2116-10|10-SRNP-102291
Ethmia hammella|[439]|BLPCK522-08|08-SRNP-100615
Ethmia hammella|[440]|BLPDW472-11|10-SRNP-112364
Ethmia hammella|[441]|ASARD142-11|INB0004238426
Ethmia hammella|[442]|BLPDB213-09|08-SRNP-106228
Ethmia hammella|[443]|BLPDK1545-09|09-SRNP-106473
Ethmia hammella|[444]|BLPDM1415-10|09-SRNP-110792
Ethmia hammella|[445]|BLPBD486-07|07-SRNP-100485
Ethmia hammella|[446]|BLPDC321-09|08-SRNP-107276
Ethmia hammella|[447]|BLPDM1426-10|09-SRNP-110803
Ethmia hammella|[448]|BLPDS224-10|10-SRNP-106848
Ethmia hammella|[449]|BLPDO449-10|10-SRNP-103031
Ethmia hammella|[450]|BLPDN2120-10|10-SRNP-102295
Ethmia hammella|[451]|BLPDK164-09|09-SRNP-105092
Ethmia hammella|[452]|ASARD176-11|INB0003231100
Ethmia hammella|[453]|BLPDN1946-10|10-SRNP-102121
Ethmia hammella|[454]|BLPDM1352-10|09-SRNP-110729
Ethmia hammella|[455]|BLPCG345-08|07-SRNP-110590
Ethmia hammella|[456]|ASARD375-11|INB0004263682
Ethmia hammella|[457]|BLPDN1308-10|10-SRNP-101483
Ethmia hammella|[458]|BLPDS736-10|10-SRNP-107360
Ethmia hammella|[459]|BLPDB214-09|08-SRNP-106229
Ethmia hammella|[460]|BLPDM2281-10|10-SRNP-100018
Ethmia hammella|[461]|BLPEB1031-11|11-SRNP-102543
Ethmia hammella|[462]|BLPDL970-10|09-SRNP-108352
Ethmia hammella|[463]|BLPDU605-11|10-SRNP-110027
Ethmia hammella|[464]|BLPDQ380-10|10-SRNP-105006
Ethmia hammella|[465]|BLPEC417-11|11-SRNP-102974
Ethmia hammella|[466]|BLPDV550-11|10-SRNP-111207
Ethmia hammella|[467]|BLPAC887-06|06-SRNP-102766
Ethmia hammella|[468]|BLPED644-11|11-SRNP-104246
[illegible]

Ethmia hammella|[466]|BLPDV330-11|10-SRNP-111207
Ethmia hammella|[467]|BLPAC887-06|06-SRNP-102766
Ethmia hammella|[468]|BLPED644-11|11-SRNP-104246
Ethmia hammella|[469]|BLPEB217-11|11-SRNP-101729
Ethmia hammella|[470]|BLPDS632-10|10-SRNP-107256
Ethmia hammella|[471]|BLPDK1613-09|09-SRNP-106541
Ethmia hammella|[472]|BLPED686-11|11-SRNP-104288
Ethmia hammella|[473]|BLPDS131-10|10-SRNP-106755
Ethmia hammella|[474]|BLPDM848-10|09-SRNP-110225
Ethmia hammella|[475]|BLPCG644-08|07-SRNP-110889
Ethmia hammella|[476]|BLPDK241-09|09-SRNP-105169
Ethmia hammella|[477]|ASARD141-11|INB0004238425
Ethmia hammella|[478]|BLPDK250-09|09-SRNP-105178
Ethmia hammella|[479]|BLPDZ782-11|11-SRNP-100394
Ethmia hammella|[480]|BLPDV1159-11|10-SRNP-111816
Ethmia hammella|[481]|ASARD372-11|INB0004263679
Ethmia hammella|[482]|BLPDK1653-09|09-SRNP-106581
Ethmia hammella|[483]|BLPDX473-11|10-SRNP-113600
Ethmia hammella|[484]|BLPDN2301-10|10-SRNP-102476
Ethmia hammella|[485]|BLPDN1948-10|10-SRNP-102123
Ethmia hammella|[486]|ASARD374-11|INB0004263681
Ethmia hammella|[487]|BLPDN1022-10|10-SRNP-101197
Ethmia hammella|[488]|BLPDU331-11|10-SRNP-109753
Ethmia hammella|[489]|BLPDN491-10|10-SRNP-100666
Ethmia hammella|[490]|BLPDC322-09|08-SRNP-107277
Ethmia hammella|[491]|BLPDB212-09|08-SRNP-106227
Ethmia hammella|[492]|BLPDM794-10|09-SRNP-110171
Ethmia hammella|[493]|BLPDN2184-10|10-SRNP-102359
Ethmia helenmillerae|[494]|BLPED137-11|11-SRNP-103739
Ethmia helenmillerae|[495]|BLPDJ337-09|09-SRNP-104719
Ethmia helenmillerae|[496]|BLPED286-11|11-SRNP-103888
Ethmia helenmillerae|[497]|BLPED136-11|11-SRNP-103738
Ethmia helenmillerae|[498]|BLPED300-11|11-SRNP-103902
Ethmia helenmillerae|[499]|BLPEC709-11|11-SRNP-103266
Ethmia helenmillerae|[500]|BLPDI857-09|09-SRNP-104289
Ethmia helenmillerae|[501]|BLPEC708-11|11-SRNP-103265
Ethmia helenmillerae|[502]|BLPEC953-11|11-SRNP-103510
Ethmia helenmillerae|[503]|BLPDJ172-09|09-SRNP-104554
Ethmia helenmillerae|[504]|BLPED405-11|11-SRNP-104007
Ethmia helenmillerae|[505]|BLPDK867-09|09-SRNP-105795
Ethmia helenmillerae|[506]|BLPED293-11|11-SRNP-103895
Ethmia helenmillerae|[507]|BLPED308-11|11-SRNP-103910
Ethmia helenmillerae|[508]|BLPEC707-11|11-SRNP-103264
Ethmia helenmillerae|[509]|BLPEC952-11|11-SRNP-103509
Ethmia helenmillerae|[510]|BLPED132-11|11-SRNP-103734
Ethmia helenmillerae|[511]|BLPDL581-10|09-SRNP-107963
Ethmia helenmillerae|[512]|MHMYS3365-13|13-SRNP-101566
Ethmia helenmillerae|[513]|BLPDK862-09|09-SRNP-105790
Ethmia helenmillerae|[514]|BLPDK1010-09|09-SRNP-105938
Ethmia helenmillerae|[515]|BLPDL486-10|09-SRNP-107868
Ethmia helenmillerae|[516]|BLPDK881-09|09-SRNP-105809
Ethmia helenmillerae|[517]|BLPED284-11|11-SRNP-103886
Ethmia helenmillerae|[518]|BLPDJ336-09|09-SRNP-104718
Ethmia helenmillerae|[519]|BLPDL482-10|09-SRNP-107864
Ethmia helenmillerae|[520]|BLPDL593-10|09-SRNP-107975
Ethmia helenmillerae|[521]|BLPED135-11|11-SRNP-103737
Ethmia helenmillerae|[522]|BLPED325-11|11-SRNP-103927
Ethmia helenmillerae|[523]|BLPED133-11|11-SRNP-103735
Ethmia helenmillerae|[524]|BLPDK888-09|09-SRNP-105816
Ethmia helenmillerae|[525]|BLPED134-11|11-SRNP-103736
Ethmia linda|[526]|BLPED1179-12|11-SRNP-104781
Ethmia linda|[527]|BLPED1082-12|11-SRNP-104684
Ethmia lichyi|[528]|BLPDT1619-10|10-SRNP-109046
Ethmia lichyi|[529]|BLPEA339-11|11-SRNP-100806
Ethmia lichyi|[530]|MHMYK1493-11|10-SRNP-71337
Ethmia lichyi|[531]|MHMYK1483-11|10-SRNP-71376
Ethmia lichyi|[532]|MHMYK1488-11|10-SRNP-71375
Ethmia lichyi|[533]|MHMYO1175-11|11-SRNP-71375
Ethmia lichyi|[534]|MHAYA003-06|06-SRNP-30720
Ethmia lichyi|[535]|MHAYA001-06|06-SRNP-30714
Ethmia lichyi|[536]|MHMYG1677-10|09-SRNP-20517
Ethmia lichyi|[537]|MHMXD1156-06|06-SRNP-31744
Ethmia lichyi|[538]|MHAYA002-06|06-SRNP-30523
Ethmia lichyi|[539]|MHAYA163-06|03-SRNP-29318
Ethmia lichyi|[540]|MHMYK1522-11|10-SRNP-22372
Ethmia lichyi|[541]|BLPBD483-07|07-SRNP-100482
Ethmia lichyi|[542]|MHMYG133-10|09-SRNP-70588
Ethmia lichyi|[543]|MHMXR163-08|07-SRNP-32627
Ethmia lichyi|[544]|MHMYK1496-11|10-SRNP-31048
Ethmia lichyi|[545]|BLPDM766-10|09-SRNP-110143
Ethmia lichyi|[546]|MHMYG1317-10|09-SRNP-22510
Ethmia lichyi|[547]|MHMYG2175-10|09-SRNP-23036
Ethmia lichyi|[548]|MHMYR279-12|11-SRNP-72108
Ethmia lichyi|[549]|ASARD185-11|INB0003230743
Ethmia lichyi|[550]|MHMXL756-07|07-SRNP-31215
Ethmia lichyi|[551]|MHMYB667-09|08-SRNP-71629
Ethmia lichyi|[552]|MHAYA162-06|04-SRNP-27125
Ethmia lichyi|[553]|MHMYK1512-11|10-SRNP-30359
Ethmia lichyi|[554]|BLPCF308-08|07-SRNP-109613
Ethmia lichyi|[555]|MHMXI897-07|07-SRNP-31106
Ethmia lichyi|[556]|MHMYK1514-11|10-SRNP-30361
Ethmia lichyi|[557]|MHMYK1521-11|10-SRNP-22438
Ethmia lichyi|[558]|ASARD992-11|INB0004222455
Ethmia lichyi|[559]|MHMXI896-07|07-SRNP-30826
Ethmia lichyi|[560]|MHMYG1318-10|09-SRNP-22511
Ethmia lichyi|[561]|BLPDY739-11|10-SRNP-115291
Ethmia lichyi|[562]|MHMYG134-10|09-SRNP-56762

Ethmia lichyi|[560]|MHMYG1318-10|09-SRNP-22511
Ethmia lichyi|[561]|BLPDY739-11|10-SRNP-115291
Ethmia lichyi|[562]|MHMYG134-10|09-SRNP-56762
Ethmia lichyi|[563]|MHMYK1495-11|10-SRNP-31046
Ethmia lichyi|[564]|MHMYO1176-11|11-SRNP-71576
Ethmia lichyi|[565]|MHAYA157-06|05-SRNP-22361
Ethmia lichyi|[566]|MHAYA164-06|05-SRNP-22648
Ethmia lichyi|[567]|MHMXF080-07|06-SRNP-34073
Ethmia lichyi|[568]|MHMXL748-07|07-SRNP-31216
Ethmia lichyi|[569]|MHAYA158-06|04-SRNP-23311
Ethmia lichyi|[570]|MHMYG1319-10|09-SRNP-22513
Ethmia lichyi|[571]|MHMYK1489-11|10-SRNP-31053
Ethmia lichyi|[572]|MHMYG2174-10|09-SRNP-23038
Ethmia lichyi|[573]|MHMYG1315-10|09-SRNP-22512
Ethmia lichyi|[574]|MHAYA004-06|06-SRNP-30526
Ethmia lichyi|[575]|MHMXL755-07|07-SRNP-31214
Ethmia lichyi|[576]|MHMYK1492-11|10-SRNP-31328
Ethmia lichyi|[577]|MHMYK1485-11|10-SRNP-71398
Ethmia lichyi|[578]|MHMXD1155-06|06-SRNP-30891
Ethmia lichyi|[579]|MHMXL746-07|07-SRNP-1750
Ethmia lichyi|[580]|MHMYN2236-13|12-SRNP-5402
Ethmia lichyi|[581]|BLPDZ141-11|10-SRNP-115548
Ethmia lichyi|[582]|BLPDT1878-10|10-SRNP-109305
Ethmia lichyi|[583]|MHMYO1171-11|10-SRNP-71060
Ethmia lichyi|[584]|BLPDK633-09|09-SRNP-105561
Ethmia lichyi|[585]|BLPDT1721-10|10-SRNP-109148
Ethmia lichyi|[586]|BLPDO194-10|10-SRNP-102776
Ethmia lichyi|[587]|MHMYT1563-13|12-SRNP-3090
Ethmia lichyi|[588]|MHMXF090-07|06-SRNP-6458
Ethmia lichyi|[589]|MHMYE1621-09|09-SRNP-4051
Ethmia lichyi|[590]|BLPDY740-11|10-SRNP-115292
Ethmia lichyi|[591]|MHAYA159-06|05-SRNP-45034
Ethmia lichyi|[592]|MHAYA161-06|05-SRNP-3596
Ethmia lichyi|[593]|MHAYA160-06|05-SRNP-3595
Ethmia lichyi|[594]|MHMXD140-06|05-SRNP-46875
Ethmia lichyi|[595]|MHMYK1525-11|10-SRNP-6706
Ethmia lichyi|[596]|MHMYK1523-11|10-SRNP-6712
Ethmia lichyi|[597]|MHMYK1516-11|10-SRNP-3636
Ethmia lichyi|[598]|MHMYO1172-11|11-SRNP-3084
Ethmia lichyi|[599]|MHMYK1497-11|10-SRNP-3005
Ethmia lichyi|[600]|MHMYK1517-11|10-SRNP-3641
Ethmia lichyi|[601]|MHMYK1520-11|10-SRNP-3637
Ethmia lichyi|[602]|MHMYO1168-11|11-SRNP-1897
Ethmia lichyi|[603]|BLPDM2110-10|09-SRNP-111487
Ethmia lichyi|[604]|MHMXF081-07|06-SRNP-5348
Ethmia lichyi|[605]|MHMYR278-12|11-SRNP-3444
Ethmia lichyi|[606]|MHMXF078-07|06-SRNP-5344
Ethmia lichyi|[607]|MHMXF082-07|06-SRNP-5347
Ethmia lichyi|[608]|MHMYK1498-11|10-SRNP-3006
Ethmia lichyi|[609]|MHMYK1527-11|10-SRNP-6708
Ethmia lichyi|[610]|MHMXD139-06|05-SRNP-46876
Ethmia lichyi|[611]|MHMXF084-07|06-SRNP-5349
Ethmia lichyi|[612]|MHMYK1524-11|10-SRNP-6709
Ethmia lichyi|[613]|MHMXF076-07|06-SRNP-5342
Ethmia lichyi|[614]|MHMXF083-07|06-SRNP-5343
Ethmia lichyi|[615]|MHMYK1510-11|10-SRNP-2212
Ethmia hendersonorum|[616]|ASARD993-11|INB0004222359
Ethmia hendersonorum|[617]|ASARD991-11|INB0004222481
Ethmia hendersonorum|[618]|ASARD990-11|INB0004222358
Ethmia hendersonorum|[619]|ASARD187-11|INB0003230938
Ethmia hendersonorum|[620]|ASARD186-11|INB0003230788
Ethmia similatella|[621]|BLPEC726-11|11-SRNP-103283
Ethmia similatella|[622]|MHMYG2253-10|09-SRNP-14032
Ethmia similatella|[623]|BLPED126-11|11-SRNP-103728
Ethmia miriamschulmanae[624]|BLPEC882-11|11-SRNP-103439
Ethmia miriamschulmanae[625]|BLPED480-11|11-SRNP-104082
Ethmiamiriamschulmanae[626]|BLPDK971-09|09-SRNP-105899
Ethmia miriamschulmanae[627]|BLPEE2148-12|12-SRNP-102147
Ethmia miriamschulmanae[628]|BLPEE2026-12|12-SRNP-102025
Ethmia miriamschulmanae[629]|BLPDL1695-10|09-SRNP-109077
Ethmia miriamschulmanae[630]|BLPDL1919-10|09-SRNP-109301
Ethmia miriamschulmanae|[631]|BLPDM141-10|09-SRNP-109518
Ethmia miriamschulmanae|[632]|MHMYS239-12|11-SRNP-56581
Ethmia miriamschulmanae[633]|BLPDL1740-10|09-SRNP-109122
Ethmia miriamschulmanae|[634]|MHMYS240-12|11-SRNP-56582
Ethmia miriamschulmanae[635]|BLPCJ344-08|07-SRNP-113409
Ethmia miriamschulmanae[636]|BLPED129-11|11-SRNP-103731
Ethmia miriamschulmanae[637]|BLPED396-11|11-SRNP-103998
Ethmia miriamschulmanae[638]|MHMYC1764-09|09-SRNP-14180
Ethmia miriamschulmanae[639]|BLPED2608-12|11-SRNP-106210
Ethmia miriamschulmanae[640]|BLPED2167-12|11-SRNP-105769
Ethmia miriamschulmanae[641]|BLPED127-11|11-SRNP-103729
Ethmia miriamschulmanae[642]|BLPED1680-12|11-SRNP-105282
Ethmia miriamschulmanae[643]|BLPED1967-12|11-SRNP-105569
Ethmia miriamschulmanae[644]|BLPDJ507-09|09-SRNP-104889
Ethmia miriamschulmanae[645]|BLPED2513-12|11-SRNP-106115
Ethmia miriamschulmanae[646]|BLPDL1557-10|09-SRNP-108939
Ethmia miriamschulmanae[647]|BLPDJ506-09|09-SRNP-104888
Ethmia miriamschulmanae[648]|BLPED648-11|11-SRNP-104250
Ethmia transversella|[649]|ASARD370-11|INB0004263677
Ethmia transversella|[650]|ASARD449-11|INB0004265045
Ethmia transversella|[651]|ASARD346-11|INB0004265028
Ethmia transversella|[652]|BLPEA237-11|11-SRNP-100704
Ethmia transversella|[653]|BLPDV854-11|10-SRNP-111511
Ethmia transversella|[654]|ASARD147-11|INB0004238431
Ethmia transversella|[655]|BLPEA238-11|11-SRNP-100705
Ethmia randyjonesi|[656]|BLPDY1338-11|10-SRNP-114465

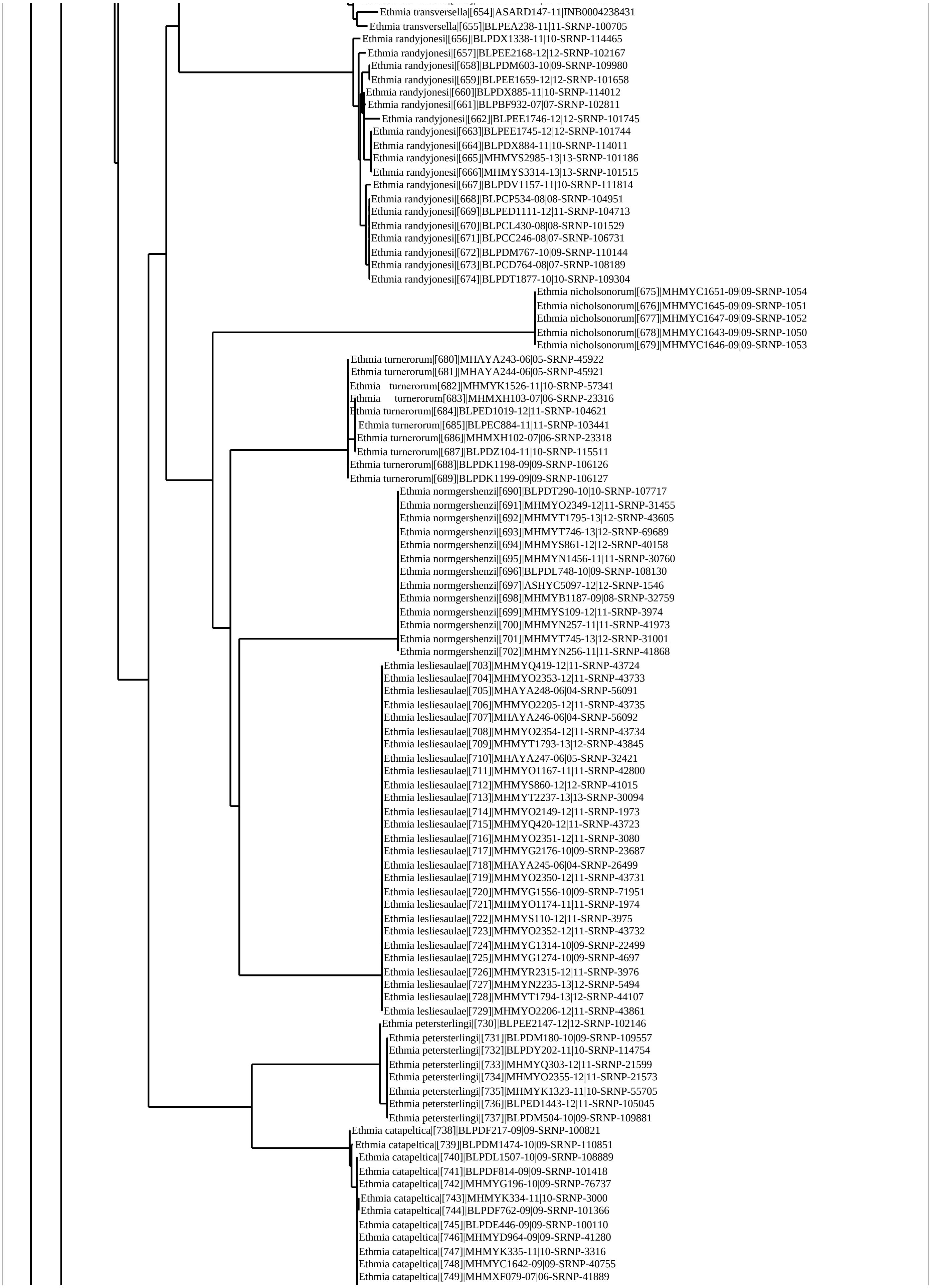
Ethmia transversella|[654]|ASARD147-11|INB0004238431
Ethmia transversella|[655]|BLPEA238-11|11-SRNP-100705
Ethmia randyjonesi|[656]|BLPDX1338-11|10-SRNP-114465
Ethmia randyjonesi|[657]|BLPEE2168-12|12-SRNP-102167
Ethmia randyjonesi|[658]|BLPDM603-10|09-SRNP-109980
Ethmia randyjonesi|[659]|BLPEE1659-12|12-SRNP-101658
Ethmia randyjonesi|[660]|BLPDX885-11|10-SRNP-114012
Ethmia randyjonesi|[661]|BLPBF932-07|07-SRNP-102811
Ethmia randyjonesi|[662]|BLPEE1746-12|12-SRNP-101745
Ethmia randyjonesi|[663]|BLPEE1745-12|12-SRNP-101744
Ethmia randyjonesi|[664]|BLPDX884-11|10-SRNP-114011
Ethmia randyjonesi|[665]|MHMYS2985-13|13-SRNP-101186
Ethmia randyjonesi|[666]|MHMYS3314-13|13-SRNP-101515
Ethmia randyjonesi|[667]|BLPDV1157-11|10-SRNP-111814
Ethmia randyjonesi|[668]|BLPCP534-08|08-SRNP-104951
Ethmia randyjonesi|[669]|BLPED1111-12|11-SRNP-104713
Ethmia randyjonesi|[670]|BLPCL430-08|08-SRNP-101529
Ethmia randyjonesi|[671]|BLPCC246-08|07-SRNP-106731
Ethmia randyjonesi|[672]|BLPDM767-10|09-SRNP-110144
Ethmia randyjonesi|[673]|BLPCD764-08|07-SRNP-108189
Ethmia randyjonesi|[674]|BLPDT1877-10|10-SRNP-109304
Ethmia nicholsonorum|[675]|MHMYC1651-09|09-SRNP-1054
Ethmia nicholsonorum|[676]|MHMYC1645-09|09-SRNP-1051
Ethmia nicholsonorum|[677]|MHMYC1647-09|09-SRNP-1052
Ethmia nicholsonorum|[678]|MHMYC1643-09|09-SRNP-1050
Ethmia nicholsonorum|[679]|MHMYC1646-09|09-SRNP-1053
Ethmia turnerorum|[680]|MHAYA243-06|05-SRNP-45922
Ethmia turnerorum|[681]|MHAYA244-06|05-SRNP-45921
Ethmia turnerorum|[682]|MHMYK1526-11|10-SRNP-57341
Ethmia turnerorum|[683]|MHMXH103-07|06-SRNP-23316
Ethmia turnerorum|[684]|BLPED1019-12|11-SRNP-104621
Ethmia turnerorum|[685]|BLPEC884-11|11-SRNP-103441
Ethmia turnerorum|[686]|MHMXH102-07|06-SRNP-23318
Ethmia turnerorum|[687]|BLPDZ104-11|10-SRNP-115511
Ethmia turnerorum|[688]|BLPDK1198-09|09-SRNP-106126
Ethmia turnerorum|[689]|BLPDK1199-09|09-SRNP-106127
Ethmia normgershenzi|[690]|BLPDT290-10|10-SRNP-107717
Ethmia normgershenzi|[691]|MHMYO2349-12|11-SRNP-31455
Ethmia normgershenzi|[692]|MHMYT1795-13|12-SRNP-43605
Ethmia normgershenzi|[693]|MHMYT746-13|12-SRNP-69689
Ethmia normgershenzi|[694]|MHMYS861-12|12-SRNP-40158
Ethmia normgershenzi|[695]|MHMYN1456-11|11-SRNP-30760
Ethmia normgershenzi|[696]|BLPDL748-10|09-SRNP-108130
Ethmia normgershenzi|[697]|ASHYC5097-12|12-SRNP-1546
Ethmia normgershenzi|[698]|MHMYB1187-09|08-SRNP-32759
Ethmia normgershenzi|[699]|MHMYS109-12|11-SRNP-3974
Ethmia normgershenzi|[700]|MHMYN257-11|11-SRNP-41973
Ethmia normgershenzi|[701]|MHMYT745-13|12-SRNP-31001
Ethmia normgershenzi|[702]|MHMYN256-11|11-SRNP-41868
Ethmia lesliesaulae|[703]|MHMYQ419-12|11-SRNP-43724
Ethmia lesliesaulae|[704]|MHMYO2353-12|11-SRNP-43733
Ethmia lesliesaulae|[705]|MHAYA248-06|04-SRNP-56091
Ethmia lesliesaulae|[706]|MHMYO2205-12|11-SRNP-43735
Ethmia lesliesaulae|[707]|MHAYA246-06|04-SRNP-56092
Ethmia lesliesaulae|[708]|MHMYO2354-12|11-SRNP-43734
Ethmia lesliesaulae|[709]|MHMYT1793-13|12-SRNP-43845
Ethmia lesliesaulae|[710]|MHAYA247-06|05-SRNP-32421
Ethmia lesliesaulae|[711]|MHMYO1167-11|11-SRNP-42800
Ethmia lesliesaulae|[712]|MHMYS860-12|12-SRNP-41015
Ethmia lesliesaulae|[713]|MHMYT2237-13|13-SRNP-30094
Ethmia lesliesaulae|[714]|MHMYO2149-12|11-SRNP-1973
Ethmia lesliesaulae|[715]|MHMYQ420-12|11-SRNP-43723
Ethmia lesliesaulae|[716]|MHMYO2351-12|11-SRNP-3080
Ethmia lesliesaulae|[717]|MHMYG2176-10|09-SRNP-23687
Ethmia lesliesaulae|[718]|MHAYA245-06|04-SRNP-26499
Ethmia lesliesaulae|[719]|MHMYO2350-12|11-SRNP-43731
Ethmia lesliesaulae|[720]|MHMYG1556-10|09-SRNP-71951
Ethmia lesliesaulae|[721]|MHMYO1174-11|11-SRNP-1974
Ethmia lesliesaulae|[722]|MHMYS110-12|11-SRNP-3975
Ethmia lesliesaulae|[723]|MHMYO2352-12|11-SRNP-43732
Ethmia lesliesaulae|[724]|MHMYG1314-10|09-SRNP-22499
Ethmia lesliesaulae|[725]|MHMYG1274-10|09-SRNP-4697
Ethmia lesliesaulae|[726]|MHMYR2315-12|11-SRNP-3976
Ethmia lesliesaulae|[727]|MHMYN2235-13|12-SRNP-5494
Ethmia lesliesaulae|[728]|MHMYT1794-13|12-SRNP-44107
Ethmia lesliesaulae|[729]|MHMYO2206-12|11-SRNP-43861
Ethmia petersterlingi|[730]|BLPEE2147-12|12-SRNP-102146
Ethmia petersterlingi|[731]|BLPDM180-10|09-SRNP-109557
Ethmia petersterlingi|[732]|BLPDY202-11|10-SRNP-114754
Ethmia petersterlingi|[733]|MHMYQ303-12|11-SRNP-21599
Ethmia petersterlingi|[734]|MHMYO2355-12|11-SRNP-21573
Ethmia petersterlingi|[735]|MHMYK1323-11|10-SRNP-55705
Ethmia petersterlingi|[736]|BLPED1443-12|11-SRNP-105045
Ethmia petersterlingi|[737]|BLPDM504-10|09-SRNP-109881
Ethmia catapeltica|[738]|BLPDF217-09|09-SRNP-100821
Ethmia catapeltica|[739]|BLPDM1474-10|09-SRNP-110851
Ethmia catapeltica|[740]|BLPDL1507-10|09-SRNP-108889
Ethmia catapeltica|[741]|BLPDF814-09|09-SRNP-101418
Ethmia catapeltica|[742]|MHMYG196-10|09-SRNP-76737
Ethmia catapeltica|[743]|MHMYK334-11|10-SRNP-3000
Ethmia catapeltica|[744]|BLPDF762-09|09-SRNP-101366
Ethmia catapeltica|[745]|BLPDE446-09|09-SRNP-100110
Ethmia catapeltica|[746]|MHMYD964-09|09-SRNP-41280
Ethmia catapeltica|[747]|MHMYK335-11|10-SRNP-3316
Ethmia catapeltica|[748]|MHMYC1642-09|09-SRNP-40755
Ethmia catapeltica|[749]|MHMXF079-07|06-SRNP-41889

Ethmia catapeltica|[747]|MHMYK335-11|10-SRNP-3316
Ethmia catapeltica|[748]|MHMYC1642-09|09-SRNP-40755
Ethmia catapeltica|[749]|MHMXF079-07|06-SRNP-41889
Ethmia catapeltica|[750]|MHMYD978-09|09-SRNP-2831
Ethmia catapeltica|[751]|MHMYG1555-10|09-SRNP-76736
Ethmia catapeltica|[752]|ASHYC5207-12|12-SRNP-1699
Ethmia catapeltica|[753]|MHMXP812-08|07-SRNP-41707
Ethmia catapeltica|[754]|MHMXH100-07|06-SRNP-43051
Ethmia catapeltica|[755]|MHMYO2204-12|11-SRNP-43753
Ethmia catapeltica|[756]|MHMYB1192-09|08-SRNP-42442
Ethmia catapeltica|[757]|MHMYB1191-09|08-SRNP-42447
Ethmia catapeltica|[758]|MHMYO2203-12|11-SRNP-43742
Ethmia catapeltica|[759]|ASHYC5208-12|12-SRNP-1700
Ethmia catapeltica|[760]|MHMYT743-13|12-SRNP-43974
Ethmia catapeltica|[761]|MHMXJ051-07|06-SRNP-44099
Ethmia catapeltica|[762]|MHMYG195-10|09-SRNP-76735
Ethmia catapeltica|[763]|BLPED1018-12|11-SRNP-104620
Ethmia catapeltica|[764]|MHMYR2199-12|11-SRNP-76291
Ethmia catapeltica|[765]|BLPDL1236-10|09-SRNP-108618
Ethmia catapeltica|[766]|MHMYD897-09|09-SRNP-2180
Ethmia catapeltica|[767]|MHMYT1796-13|12-SRNP-43130
Ethmia catapeltica|[768]|MHMYT2290-13|12-SRNP-4120
Ethmia catapeltica|[769]|MHMYT2313-13|13-SRNP-75302
Ethmia catapeltica|[770]|MHMYB1193-09|08-SRNP-42446
Ethmia catapeltica|[771]|MHMYT2312-13|13-SRNP-75299
Ethmia catapeltica|[772]|MHMYN258-11|11-SRNP-41880
Ethmia catapeltica|[773]|MHMXF075-07|06-SRNP-43050
Ethmia catapeltica|[774]|MHMYK681-11|10-SRNP-3435
Ethmia catapeltica|[775]|MHMYT747-13|12-SRNP-43344
Ethmia catapeltica|[776]|MHMYB1188-09|09-SRNP-410
Ethmia catapeltica|[777]|MHMYB1186-09|08-SRNP-4960
Ethmia catapeltica|[778]|MHMYK535-11|10-SRNP-4226
Ethmia catapeltica|[779]|MHMYT744-13|12-SRNP-43969
Ethmia catapeltica|[780]|MHMYT1931-13|12-SRNP-3105
Ethmia catapeltica|[781]|MHMYK333-11|10-SRNP-42304
Ethmia catapeltica|[782]|MHMXH099-07|06-SRNP-43052
Ethmia catapeltica|[783]|MHMYB1190-09|08-SRNP-42445
Ethmia catapeltica|[784]|MHMYK1491-11|10-SRNP-42305
Ethmia catapeltica|[785]|MHMXH101-07|06-SRNP-43542
Ethmia catapeltica|[786]|MHMYC1655-09|09-SRNP-40756
Ethmia catapeltica|[787]|MHMYJ1256-11|10-SRNP-4552
Ethmia catapeltica|[788]|MHMYG2358-10|10-SRNP-40184
Ethmia catapeltica|[789]|MHMYG2298-10|10-SRNP-40183
Ethmia catapeltica|[790]|MHMYT2289-13|12-SRNP-4178
Ethmia catapeltica|[791]|MHMXF088-07|06-SRNP-43133
Ethmia catapeltica|[792]|MHMYS052-12|11-SRNP-44543
Ethmia catapeltica|[793]|MHMXJ052-07|06-SRNP-43792
Ethmia catapeltica|[794]|MHMYG1456-10|09-SRNP-42501
Ethmia catapeltica|[795]|MHMYR2314-12|11-SRNP-44541
Ethmia catapeltica|[796]|MHMXP813-08|07-SRNP-41700
Ethmia catapeltica|[797]|MHMXF077-07|06-SRNP-43541
Ethmia catapeltica|[798]|BLPDM2040-10|09-SRNP-111417
Ethmia catapeltica|[799]|MHMXD1157-06|06-SRNP-41888
Ethmia catapeltica|[800]|MHMYE1835-09|09-SRNP-75407
Ethmia catapeltica|[801]|MHMYK1487-11|10-SRNP-42303
Ethmia catapeltica|[802]|MHMYG334-10|09-SRNP-6587
Ethmia catapeltica|[803]|MHMYB1185-09|08-SRNP-4959
Ethmia catapeltica|[804]|MHMYG2317-10|10-SRNP-484
Ethmia catapeltica|[805]|MHMYT1797-13|12-SRNP-43131
Ethmia catapeltica|[806]|MHMXD1158-06|06-SRNP-41887
Ethmia catapeltica|[807]|BLPDM1253-10|09-SRNP-110630
Ethmia catapeltica|[808]|MHMYT2210-13|12-SRNP-5442
Ethmia catapeltica|[809]|MHMYC1644-09|09-SRNP-409
Ethmia catapeltica|[810]|MHMYE1623-09|09-SRNP-75501
Ethmia catapeltica|[811]|MHMYT1932-13|12-SRNP-3106
Ethmia catapeltica|[812]|BLPDK2282-09|09-SRNP-107210
Ethmia catapeltica|[813]|BLPDF812-09|09-SRNP-101416
Ethmia catapeltica|[814]|BLPDF811-09|09-SRNP-101415
Ethmia catapeltica|[815]|MHMYK281-11|10-SRNP-2910
Ethmia catapeltica|[816]|MHMYK282-11|10-SRNP-2994
Ethmia catapeltica|[817]|BLPDF810-09|09-SRNP-101414
Ethmia catapeltica|[818]|BLPDF042-09|09-SRNP-100646
Ethmia catapeltica|[819]|MHMYD1127-09|09-SRNP-2065
Ethmia catapeltica|[820]|BLPDE401-09|09-SRNP-100065
Ethmia catapeltica|[821]|MHMYS334-12|11-SRNP-44538
Ethmia catapeltica|[822]|MHMYG194-10|09-SRNP-6589
Ethmia catapeltica|[823]|MHMYB1189-09|08-SRNP-42443
Ethmia catapeltica|[824]|MHMYG197-10|09-SRNP-5705
Ethmia catapeltica|[825]|MHMYE1622-09|09-SRNP-75396
Ethmia catapeltica|[826]|BLPDM648-10|09-SRNP-110025
Ethmia catapeltica|[827]|MHMYK1370-11|10-SRNP-300
Ethmia laphamorum|[828]|BLPED870-11|11-SRNP-104472
Ethmia laphamorum|[829]|BLPED2585-12|11-SRNP-106187
Ethmia laphamorum|[830]|BLPDI870-09|09-SRNP-104302
Ethmia laphamorum|[831]|BLPDJ277-09|09-SRNP-104659
Ethmia laphamorum|[832]|MHMYS2809-13|13-SRNP-101010
Ethmia laphamorum|[833]|BLPAA142-06|06-SRNP-100141
Ethmia laphamorum|[834]|BLPED2128-12|11-SRNP-105730
Ethmia laphamorum|[835]|MHMYS2810-13|13-SRNP-101011
Ethmia laphamorum|[836]|BLPED2065-12|11-SRNP-105667
Ethmia laphamorum|[837]|BLPED054-11|11-SRNP-103656
Ethmia laphamorum|[838]|BLPDD752-09|08-SRNP-108647
Ethmia laphamorum|[839]|BLPBH826-07|07-SRNP-104585
Ethmia laphamorum|[840]|BLPED2064-12|11-SRNP-105666
Ethmia laphamorum|[841]|BLPDK825-09|09-SRNP-105753
Ethmia laphamorum|[842]|BLPDM1910-10|09-SRNP-111287
Ethmia laphamorum|[843]|BLPED2068-12|11-SRNP-105670

Ethmia laphamorum|[841]|BLPDK825-09|09-SRNP-105753
Ethmia laphamorum|[842]|BLPDM1910-10|09-SRNP-111287
Ethmia laphamorum|[843]|BLPED2068-12|11-SRNP-105670
Ethmia laphamorum|[844]|BLPED2067-12|11-SRNP-105669
Ethmia laphamorum|[845]|BLPDM1735-10|09-SRNP-111112
Ethmia laphamorum|[846]|BLPED508-11|11-SRNP-104110
Ethmia laphamorum|[847]|MHMYK1494-11|10-SRNP-13173
Ethmia laphamorum|[848]|BLPEC883-11|11-SRNP-103440
Ethmia laphamorum|[849]|BLPAE714-06|06-SRNP-104473
Ethmia laphamorum|[850]|BLPDJ045-09|09-SRNP-104427
Ethmia laphamorum|[851]|BLPCI505-08|07-SRNP-112630
Ethmia laphamorum|[852]|BLPDJ281-09|09-SRNP-104663
Ethmia laphamorum|[853]|BLPED2233-12|11-SRNP-105835
Ethmialaphamorum|[854]|BLPCI234-08|07-SRNP-112359
Ethmialaphamorum|[855]|BLPAE709-06|06-SRNP-104468
Ethmia laphamorum|[856]|BLPCI503-08|07-SRNP-112628
Ethmia laphamorum|[857]|BLPED2129-12|11-SRNP-105731
Ethmia laphamorum|[858]|BLPDJ461-09|09-SRNP-104843
Ethmia laphamorum|[859]|BLPCI507-08|07-SRNP-112632
Ethmia laphamorum|[860]|BLPED2069-12|11-SRNP-105671
Ethmia laphamorum|[861]|BLPED055-11|11-SRNP-103657
Ethmia laphamorum|[862]|BLPDJ278-09|09-SRNP-104660
Ethmialaphamorum|[863]|BLPEC1007-11|11-SRNP-103564
Ethmialaphamorum|[864]|BLPDM1895-10|09-SRNP-111272
Ethmia laphamorum|[865]|BLPAA150-06|06-SRNP-100149
Ethmia laphamorum|[866]|BLPBH824-07|07-SRNP-104583
Ethmia laphamorum|[867]|BLPED2584-12|11-SRNP-106186
Ethmia laphamorum|[868]|BLPEC820-11|11-SRNP-103377
Ethmia laphamorum|[869]|BLPCI508-08|07-SRNP-112633
Ethmia laphamorum|[870]|BLPED2063-12|11-SRNP-105665
Ethmia laphamorum|[871]|BLPED2062-12|11-SRNP-105664
Ethmia laphamorum|[872]|BLPED2131-12|11-SRNP-105733
Ethmia laphamorum|[873]|BLPCI233-08|07-SRNP-112358
Ethmia laphamorum|[874]|BLPED053-11|11-SRNP-103655
Ethmialaphamorum|[875]|BLPAE523-06|06-SRNP-104282
Ethmialaphamorum|[876]|BLPED649-11|11-SRNP-104251
Ethmialaphamorum|[877]|BLPED2327-12|11-SRNP-105929
Ethmia laphamorum|[878]|BLPAE711-06|06-SRNP-104470
Ethmia laphamorum|[879]|BLPED2234-12|11-SRNP-105836
Ethmia laphamorum|[880]|BLPDM711-10|09-SRNP-110088
Ethmia laphamorum|[881]|BLPDJ134-09|09-SRNP-104516
Ethmia laphamorum|[882]|BLPDM712-10|09-SRNP-110089
Ethmia laphamorum|[883]|BLPED2236-12|11-SRNP-105838
Ethmia laphamorum|[884]|BLPDI869-09|09-SRNP-104301
Ethmia laphamorum|[885]|BLPED2130-12|11-SRNP-105732
Ethmia laphamorum|[886]|BLPDI871-09|09-SRNP-104303
Ethmia laphamorum|[887]|BLPDM1734-10|09-SRNP-111111
Ethmia laphamorum|[888]|BLPED2402-12|11-SRNP-106004
Ethmia laphamorum|[889]|BLPCA749-08|07-SRNP-105354
Ethmia laphamorum|[890]|BLPED2159-12|11-SRNP-105761
Ethmia laphamorum|[891]|BLPED052-11|11-SRNP-103654
Ethmia laphamorum|[892]|BLPAE710-06|06-SRNP-104469
Ethmia laphamorum|[893]|BLPED056-11|11-SRNP-103658
Ethmia laphamorum|[894]|MHMYS2811-13|13-SRNP-101012
Ethmia laphamorum|[895]|BLPED650-11|11-SRNP-104252
Ethmia laphamorum|[896]|BLPDM1911-10|09-SRNP-111288
Ethmia laphamorum|[897]|BLPEC1008-11|11-SRNP-103565
Ethmialaphamorum|[898]|BLPDM1896-10|09-SRNP-111273
Ethmialaphamorum|[899]|BLPEC818-11|11-SRNP-103375
Ethmia laphamorum|[900]|BLPDE037-09|08-SRNP-108873
Ethmialaphamorum|[901]|BLPDE214-09|08-SRNP-109049
Ethmialaphamorum|[902]|BLPDJ280-09|09-SRNP-104662
Ethmia laphamorum|[903]|BLPDM713-10|09-SRNP-110090
Ethmia laphamorum|[904]|BLPED051-11|11-SRNP-103653
Ethmia laphamorum|[905]|BLPAF802-07|06-SRNP-105501
Ethmia laphamorum|[906]|BLPAE712-06|06-SRNP-104471
Ethmia laphamorum|[907]|BLPCI504-08|07-SRNP-112629
Ethmia laphamorum|[908]|BLPEC819-11|11-SRNP-103376
Ethmia laphamorum|[909]|BLPED2066-12|11-SRNP-105668
Ethmia laphamorum|[910]|BLPDE213-09|08-SRNP-109048
Ethmia laphamorum|[911]|BLPDJ135-09|09-SRNP-104517
Ethmia laphamorum|[912]|BLPCI506-08|07-SRNP-112631
Ethmia nigritaenia|[913]|BLPDE036-09|08-SRNP-108872
Ethmia nigritaenia|[914]|BLPEC1006-11|11-SRNP-103563
Ethmia nigritaenia|[915]|BLPAG263-07|06-SRNP-105902
Ethmia nigritaenia|[916]|BLPCA748-08|07-SRNP-105353
Ethmia nigritaenia|[917]|BLPDJ279-09|09-SRNP-104661
Ethmia nigritaenia|[918]|BLPAE713-06|06-SRNP-104472
Ethmia nigritaenia|[919]|BLPAA564-06|06-SRNP-100563
Ethmia nigritaenia|[920]|BLPED869-11|11-SRNP-104471
Ethmia nigritaenia|[921]|BLPED050-11|11-SRNP-103652
Ethmia nigritaenia|[922]|BLPED929-11|11-SRNP-104531
Ethmia nigritaenia|[923]|BLPEC1005-11|11-SRNP-103562
Ethmia nigritaenia|[924]|BLPDM1897-10|09-SRNP-111274
Ethmia nigritaenia|[925]|MHAYA156-06|93-SRNP-4250
Ethmia nigritaenia|[926]|BLPED2235-12|11-SRNP-105837
Ethmia nigritaenia|[927]|BLPED745-11|11-SRNP-104347
Ethmia nigritaenia|[928]|BLPEC737-11|11-SRNP-103294
Ethmia baliostola|[929]|BLPDR571-10|10-SRNP-106196
Ethmia baliostola|[930]|BLPDI310-09|09-SRNP-103742
Ethmia baliostola|[931]|MHMYD939-09|09-SRNP-40650
Ethmia baliostola|[932]|MHMYK1504-11|10-SRNP-55253
Ethmia baliostola|[933]|BLPBF144-07|07-SRNP-102023
Ethmia baliostola|[934]|BLPAC882-06|06-SRNP-102761
Ethmia baliostola|[935]|BLPAC883-06|06-SRNP-102762
Ethmia baliostola|[936]|MHMYK1490-11|10-SRNP-42326
Ethmia baliostola|[937]|[illegible]

Ethmia baliostola|[934]|BLPAC882-06|06-SRNP-102761
Ethmia baliostola|[935]|BLPAC883-06|06-SRNP-102762
Ethmia baliostola|[936]|MHMYK1490-11|10-SRNP-42326
Ethmia baliostola|[937]|MHMYG1678-10|09-SRNP-23045
Ethmia baliostola|[938]|MHMYC1652-09|09-SRNP-40621
Ethmia baliostola|[939]|BLPEB596-11|11-SRNP-102108
Ethmia baliostola|[940]|BLPCM736-08|08-SRNP-102333
Ethmia baliostola|[941]|BLPCP631-08|08-SRNP-105048
Ethmia baliostola|[942]|MHMYK1505-11|10-SRNP-55291
Ethmia baliostola|[943]|MHMYK1513-11|10-SRNP-55277
Ethmia baliostola|[944]|BLPDS074-10|10-SRNP-106698
Ethmia baliostola|[945]|MHMYC1654-09|09-SRNP-40635
Ethmia baliostola|[946]|MHMYC1649-09|09-SRNP-40644
Ethmia baliostola|[947]|MHMXF085-07|06-SRNP-4332
Ethmia baliostola|[948]|BLPDH789-09|09-SRNP-103273
Ethmia baliostola|[949]|BLPAD185-06|06-SRNP-103004
Ethmia baliostola|[950]|MHAYA155-06|05-SRNP-22637
Ethmia baliostola|[951]|BLPED507-11|11-SRNP-104109
Ethmia baliostola|[952]|BLPAD186-06|06-SRNP-103005
Ethmia baliostola|[953]|MHMYK1511-11|10-SRNP-41340
Ethmia baliostola|[954]|BLPDS377-10|10-SRNP-107001
Ethmia baliostola|[955]|MHMYG1554-10|09-SRNP-23049
Ethmia baliostola|[956]|MHMYC1650-09|09-SRNP-40633
Ethmia baliostola|[957]|MHMYT1562-13|12-SRNP-41602
Ethmia baliostola|[958]|BLPDL1057-10|09-SRNP-108439
Ethmia baliostola|[959]|MHMYG1316-10|09-SRNP-23044
Ethmia baliostola|[960]|MHMYD1145-09|09-SRNP-40645
Ethmia baliostola|[961]|MHMYC1743-09|09-SRNP-40632
Ethmia baliostola|[962]|MHMYD933-09|09-SRNP-40661
Ethmia baliostola|[963]|BLPDH788-09|09-SRNP-103272
Ethmia baliostola|[964]|MHAYA152-06|05-SRNP-22239
Ethmia baliostola|[965]|BLPAC885-06|06-SRNP-102764
Ethmia baliostola|[966]|MHMXD1159-06|06-SRNP-20084
Ethmia baliostola|[967]|BLPDW379-11|10-SRNP-112271
Ethmia baliostola|[968]|MHMYO1169-11|11-SRNP-69909
Ethmia baliostola|[969]|BLPED689-11|11-SRNP-104291
Ethmia baliostola|[970]|MHMYK1515-11|09-SRNP-22104
Ethmia baliostola|[971]|MHMYO1170-11|11-SRNP-69908
Ethmia baliostola|[972]|MHMXH108-07|06-SRNP-21628
Ethmia baliostola|[973]|MHMYK1518-11|10-SRNP-20938
Ethmia baliostola|[974]|BLPBE085-07|07-SRNP-101024
Ethmia baliostola|[975]|BLPBD660-07|07-SRNP-100659
Ethmia baliostola|[976]|MHMYT1564-13|12-SRNP-41604
Ethmia baliostola|[977]|MHMYC1656-09|09-SRNP-40631
Ethmia baliostola|[978]|BLPCM586-08|08-SRNP-102183
Ethmia baliostola|[979]|BLPED1142-12|11-SRNP-104744
Ethmia baliostola|[980]|BLPDW378-11|10-SRNP-112270
Ethmia baliostola|[981]|MHMYK1502-11|10-SRNP-55292
Ethmia baliostola|[982]|MHMYD1146-09|09-SRNP-40643
Ethmia baliostola|[983]|MHMYD936-09|09-SRNP-40656
Ethmia baliostola|[984]|MHMXD135-06|05-SRNP-22492
Ethmia baliostola|[985]|MHAYA153-06|05-SRNP-22231
Ethmia baliostola|[986]|MHAYA166-06|05-SRNP-22484
Ethmia baliostola|[987]|MHMYK1484-11|10-SRNP-42325
Ethmia baliostola|[988]|BLPDT337-10|10-SRNP-107764
Ethmia baliostola|[989]|BLPAB656-06|06-SRNP-101595
Ethmia baliostola|[990]|MHMXD134-06|05-SRNP-22486
Ethmia baliostola|[991]|BLPAC884-06|06-SRNP-102763
Ethmia baliostola|[992]|BLPED566-11|11-SRNP-104168
Ethmia baliostola|[993]|MHMYK1519-11|10-SRNP-20941
Ethmia baliostola|[994]|BLPAD184-06|06-SRNP-103003
Ethmia baliostola|[995]|MHMYK1508-11|10-SRNP-55250
Ethmia baliostola|[996]|MHMYO1173-11|11-SRNP-69911
Ethmia baliostola|[997]|MHMXD136-06|06-SRNP-20088
Ethmia baliostola|[998]|MHMYC1648-09|09-SRNP-40639
Ethmia baliostola|[999]|MHMYG1679-10|09-SRNP-23047
Ethmia baliostola|[1000]|MHAYA165-06|03-SRNP-29320
Ethmia baliostola|[1001]|BLPED567-11|11-SRNP-104169
Ethmia baliostola|[1002]|MHMYC1653-09|09-SRNP-40638
Ethmia baliostola|[1003]|MHMYK1506-11|10-SRNP-55298
Ethmia baliostola|[1004]|BLPED1143-12|11-SRNP-104745
Ethmia baliostola|[1005]|MHMXH104-07|06-SRNP-21627
Ethmia baliostola|[1006]|BLPDL1058-10|09-SRNP-108440
Ethmia baliostola|[1007]|ASARD200-11|INB0004249211
Ethmia baliostola|[1008]|MHMYG1313-10|09-SRNP-21564
Ethmia baliostola|[1009]|MHMYS3406-13|13-SRNP-101607
Ethmia baliostola|[1010]|MHAYA167-06|05-SRNP-22404
Ethmia baliostola|[1011]|MHMYC1641-09|09-SRNP-40634
Ethmia baliostola|[1012]|BLPDS376-10|10-SRNP-107000
Ethmia baliostola|[1013]|BLPCM053-08|08-SRNP-101650
Ethmia baliostola|[1014]|BLPED1112-12|11-SRNP-104714
Ethmia baliostola|[1015]|MHMYK1503-11|10-SRNP-41358
Ethmia baliostola|[1016]|MHMYK1509-11|10-SRNP-55300
Ethmia baliostola|[1017]|MHAYA169-06|05-SRNP-22238
Ethmia baliostola|[1018]|MHMXH107-07|06-SRNP-21624
Ethmia baliostola|[1019]|MHAYA154-06|05-SRNP-22230
Ethmia baliostola|[1020]|MHMXD137-06|05-SRNP-22489
Ethmia baliostola|[1021]|MHMXH106-07|06-SRNP-21626
Ethmia baliostola|[1022]|BLPAC002-06|06-SRNP-101881
Ethmia baliostola|[1023]|BLPED1141-12|11-SRNP-104743
Ethmia baliostola|[1024]|MHMYG1676-10|09-SRNP-21565
Ethmia baliostola|[1025]|MHMXH105-07|06-SRNP-22854
Ethmia baliostola|[1026]|MHMYK1507-11|10-SRNP-41338
Ethmia baliostola|[1027]|BLPDK165-09|09-SRNP-105093
Ethmia baliostola|[1028]|MHAYA168-06|05-SRNP-22236
Ethmia baliostola|[1029]|BLPAC886-06|06-SRNP-102765
Ethmia baliostola|[1030]|BLPCG646-08|07-SRNP-110891

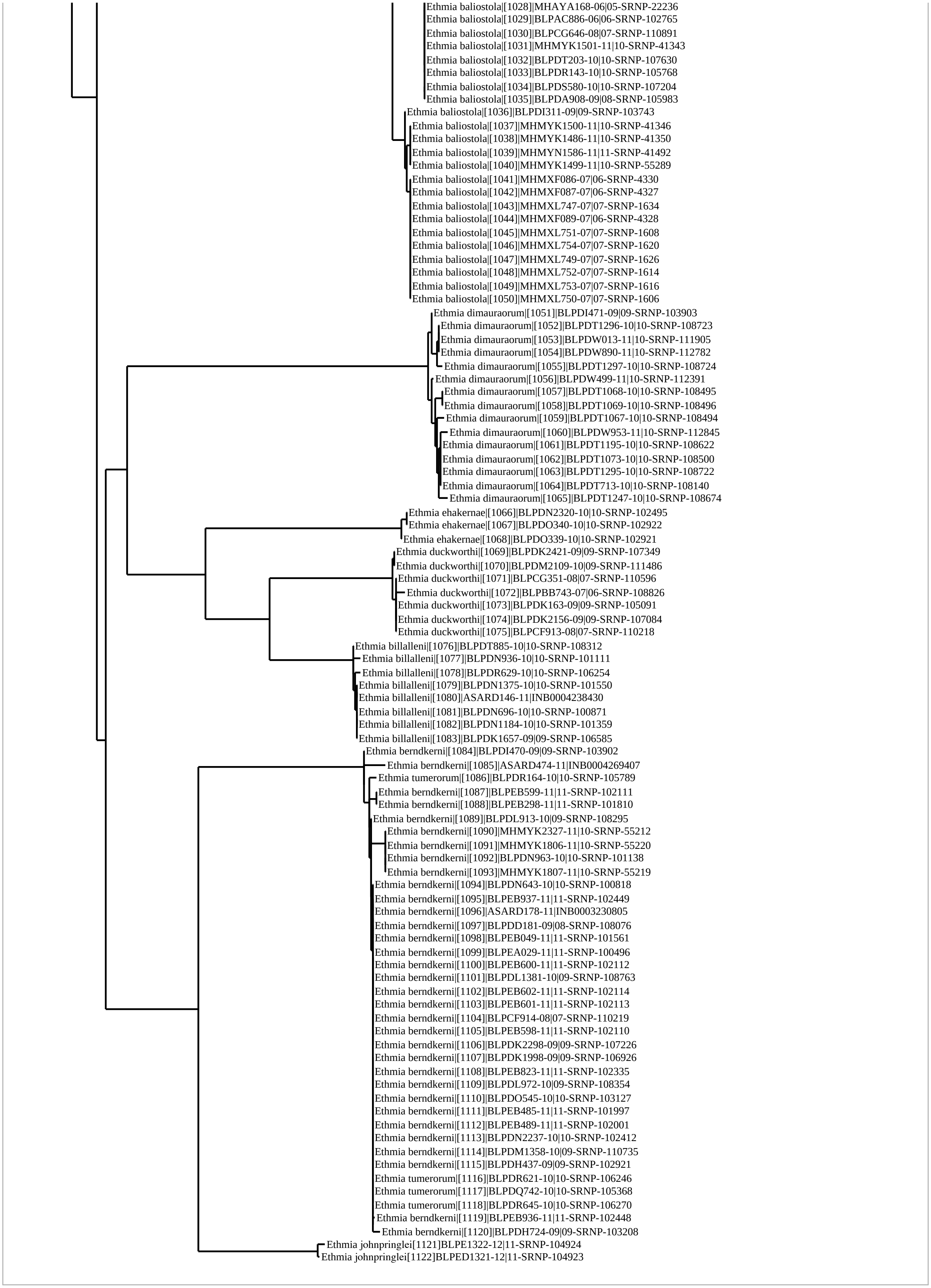
Ethmia baliostola|[1028]|MHAYA168-06|05-SRNP-22236
Ethmia baliostola|[1029]|BLPAC886-06|06-SRNP-102765
Ethmia baliostola|[1030]|BLPCG646-08|07-SRNP-110891
Ethmia baliostola|[1031]|MHMYK1501-11|10-SRNP-41343
Ethmia baliostola|[1032]|BLPDT203-10|10-SRNP-107630
Ethmia baliostola|[1033]|BLPDR143-10|10-SRNP-105768
Ethmia baliostola|[1034]|BLPDS580-10|10-SRNP-107204
Ethmia baliostola|[1035]|BLPDA908-09|08-SRNP-105983
Ethmia baliostola|[1036]|BLPDI311-09|09-SRNP-103743
Ethmia baliostola|[1037]|MHMYK1500-11|10-SRNP-41346
Ethmia baliostola|[1038]|MHMYK1486-11|10-SRNP-41350
Ethmia baliostola|[1039]|MHMYN1586-11|11-SRNP-41492
Ethmia baliostola|[1040]|MHMYK1499-11|10-SRNP-55289
Ethmia baliostola|[1041]|MHMXF086-07|06-SRNP-4330
Ethmia baliostola|[1042]|MHMXF087-07|06-SRNP-4327
Ethmia baliostola|[1043]|MHMXL747-07|07-SRNP-1634
Ethmia baliostola|[1044]|MHMXF089-07|06-SRNP-4328
Ethmia baliostola|[1045]|MHMXL751-07|07-SRNP-1608
Ethmia baliostola|[1046]|MHMXL754-07|07-SRNP-1620
Ethmia baliostola|[1047]|MHMXL749-07|07-SRNP-1626
Ethmia baliostola|[1048]|MHMXL752-07|07-SRNP-1614
Ethmia baliostola|[1049]|MHMXL753-07|07-SRNP-1616
Ethmia baliostola|[1050]|MHMXL750-07|07-SRNP-1606
Ethmia dimauraorum|[1051]|BLPDI471-09|09-SRNP-103903
Ethmia dimauraorum|[1052]|BLPDT1296-10|10-SRNP-108723
Ethmia dimauraorum|[1053]|BLPDW013-11|10-SRNP-111905
Ethmia dimauraorum|[1054]|BLPDW890-11|10-SRNP-112782
Ethmia dimauraorum|[1055]|BLPDT1297-10|10-SRNP-108724
Ethmia dimauraorum|[1056]|BLPDW499-11|10-SRNP-112391
Ethmia dimauraorum|[1057]|BLPDT1068-10|10-SRNP-108495
Ethmia dimauraorum|[1058]|BLPDT1069-10|10-SRNP-108496
Ethmia dimauraorum|[1059]|BLPDT1067-10|10-SRNP-108494
Ethmia dimauraorum|[1060]|BLPDW953-11|10-SRNP-112845
Ethmia dimauraorum|[1061]|BLPDT1195-10|10-SRNP-108622
Ethmia dimauraorum|[1062]|BLPDT1073-10|10-SRNP-108500
Ethmia dimauraorum|[1063]|BLPDT1295-10|10-SRNP-108722
Ethmia dimauraorum|[1064]|BLPDT713-10|10-SRNP-108140
Ethmia dimauraorum|[1065]|BLPDT1247-10|10-SRNP-108674
Ethmia ehakernae|[1066]|BLPDN2320-10|10-SRNP-102495
Ethmia ehakernae|[1067]|BLPDO340-10|10-SRNP-102922
Ethmia ehakernae|[1068]|BLPDO339-10|10-SRNP-102921
Ethmia duckworthi|[1069]|BLPDK2421-09|09-SRNP-107349
Ethmia duckworthi|[1070]|BLPDM2109-10|09-SRNP-111486
Ethmia duckworthi|[1071]|BLPCG351-08|07-SRNP-110596
Ethmia duckworthi|[1072]|BLPBB743-07|06-SRNP-108826
Ethmia duckworthi|[1073]|BLPDK163-09|09-SRNP-105091
Ethmia duckworthi|[1074]|BLPDK2156-09|09-SRNP-107084
Ethmia duckworthi|[1075]|BLPCF913-08|07-SRNP-110218
Ethmia billalleni|[1076]|BLPDT885-10|10-SRNP-108312
Ethmia billalleni|[1077]|BLPDN936-10|10-SRNP-101111
Ethmia billalleni|[1078]|BLPDR629-10|10-SRNP-106254
Ethmia billalleni|[1079]|BLPDN1375-10|10-SRNP-101550
Ethmia billalleni|[1080]|ASARD146-11|INB0004238430
Ethmia billalleni|[1081]|BLPDN696-10|10-SRNP-100871
Ethmia billalleni|[1082]|BLPDN1184-10|10-SRNP-101359
Ethmia billalleni|[1083]|BLPDK1657-09|09-SRNP-106585
Ethmia berndkerni|[1084]|BLPDI470-09|09-SRNP-103902
Ethmia berndkerni|[1085]|ASARD474-11|INB0004269407
Ethmia tumerorum|[1086]|BLPDR164-10|10-SRNP-105789
Ethmia berndkerni|[1087]|BLPEB599-11|11-SRNP-102111
Ethmia berndkerni|[1088]|BLPEB298-11|11-SRNP-101810
Ethmia berndkerni|[1089]|BLPDL913-10|09-SRNP-108295
Ethmia berndkerni|[1090]|MHMYK2327-11|10-SRNP-55212
Ethmia berndkerni|[1091]|MHMYK1806-11|10-SRNP-55220
Ethmia berndkerni|[1092]|BLPDN963-10|10-SRNP-101138
Ethmia berndkerni|[1093]|MHMYK1807-11|10-SRNP-55219
Ethmia berndkerni|[1094]|BLPDN643-10|10-SRNP-100818
Ethmia berndkerni|[1095]|BLPEB937-11|11-SRNP-102449
Ethmia berndkerni|[1096]|ASARD178-11|INB0003230805
Ethmia berndkerni|[1097]|BLPDD181-09|08-SRNP-108076
Ethmia berndkerni|[1098]|BLPEB049-11|11-SRNP-101561
Ethmia berndkerni|[1099]|BLPEA029-11|11-SRNP-100496
Ethmia berndkerni|[1100]|BLPEB600-11|11-SRNP-102112
Ethmia berndkerni|[1101]|BLPDL1381-10|09-SRNP-108763
Ethmia berndkerni|[1102]|BLPEB602-11|11-SRNP-102114
Ethmia berndkerni|[1103]|BLPEB601-11|11-SRNP-102113
Ethmia berndkerni|[1104]|BLPCF914-08|07-SRNP-110219
Ethmia berndkerni|[1105]|BLPEB598-11|11-SRNP-102110
Ethmia berndkerni|[1106]|BLPDK2298-09|09-SRNP-107226
Ethmia berndkerni|[1107]|BLPDK1998-09|09-SRNP-106926
Ethmia berndkerni|[1108]|BLPEB823-11|11-SRNP-102335
Ethmia berndkerni|[1109]|BLPDL972-10|09-SRNP-108354
Ethmia berndkerni|[1110]|BLPDO545-10|10-SRNP-103127
Ethmia berndkerni|[1111]|BLPEB485-11|11-SRNP-101997
Ethmia berndkerni|[1112]|BLPEB489-11|11-SRNP-102001
Ethmia berndkerni|[1113]|BLPDN2237-10|10-SRNP-102412
Ethmia berndkerni|[1114]|BLPDM1358-10|09-SRNP-110735
Ethmia berndkerni|[1115]|BLPDH437-09|09-SRNP-102921
Ethmia tumerorum|[1116]|BLPDR621-10|10-SRNP-106246
Ethmia tumerorum|[1117]|BLPDQ742-10|10-SRNP-105368
Ethmia tumerorum|[1118]|BLPDR645-10|10-SRNP-106270
Ethmia berndkerni|[1119]|BLPEB936-11|11-SRNP-102448
Ethmia berndkerni|[1120]|BLPDH724-09|09-SRNP-103208
Ethmia johnpringlei[1121]BLPE1322-12|11-SRNP-104924
Ethmia johnpringlei[1122]BLPED1321-12|11-SRNP-104923
